# Natural arsenic with a unique order structure: potential for new quantum materials

**DOI:** 10.1038/s41598-019-42561-8

**Published:** 2019-04-18

**Authors:** Akira Yoshiasa, Makoto Tokuda, Masaaki Misawa, Fuyuki Shimojo, Koichi Momma, Ritsuro Miyawaki, Satoshi Matsubara, Akihiko Nakatsuka, Kazumasa Sugiyama

**Affiliations:** 1 Faculty of Advanced Science and Technology, Kumamoto University, Kumamoto, 860-8555 Kumamoto, Japan; 2 Department of Geology, National Science Museum, Tokyo, 169-0073 Japan; 3 Graduate School of Sciences and Technology for Innovation, Yamaguchi University, Ube, 755-8611 Japan; 4 Institute for Materials Research, Tohoku Univ, Sendai, 980-8577 Japan

**Keywords:** Mineralogy, Solid-state chemistry, Electronic properties and materials, Electronic devices

## Abstract

Study of arsenic (As) provides guidelines for the development of next-generation materials. We clarify the unique structure of the third crystalline polymorph of natural As (*Pnm2*_1_-As) by crystallographical experiment and the electronic structure by first-principles computational method. The crystal structure of *Pnm2*_1_-As is a novel structure in which the basic portions of semi-metalic grey-As and semi-conductor black-As are alternately arranged at the atomic level. For both covalent and van der Waals bonding, the contributions of *sd* and *pd* hybridizations are important. Van der Waals bonding characteristics and *d* orbital contributions can be varied by control of layer stacking. Total charges are clearly divided into positive and negative in the same elements for the grey-As and black-As portions, respectively, is of importance. The sequence in which one-dimensional electron donor and acceptor portions alternate in the layer will be the first description.

## Introduction

### Crystalline polymorph of arsenic

A third crystalline polymorph of natural arsenic was discovered in Japan and named pararsenolamprite^1^. Pararsenolamprite is more resistant to alteration by weathering or oxidation than grey arsenic ($R\bar{{3}}m$-As). The possibility for two space groups *(Pnm2*_1_ is one of the proposed) was pointed out^1^ and the crystal structure was not determined. Determination of the unknown structure requires measurement of a wide range of diffraction peak intensities. Here, we clarify the unique structure of this third crystalline mineral phase of arsenic (pararsenolamprite) by superior experimental equipment and its electronic structure by simulation and discuss its possibility in the creation of new functional materials. Pararsenolamprite holds precisely the intermediate structure in which structural $R\bar{{3}}m$-As and *Bmab*-As are regularly arranged.

Electric properties of semiconductor to metal in group-V elements have been extensively studied in the fields of physics, chemistry and materials sciences^1–6^. The series P, As and Sb in group V elements shows a gradation of properties from non-metal to metal. Several phases in P, As and Sb have superconducting properties^7–9^. The physical properties in group-V elements are highly anisotropic due to asymmetric chemical bonding character. Non-simple structures are described as follows: covalently bonded atomic layers are weakly held together by van der Waals’ force, can easily cleave in the corresponding plane, and design a two-dimensional structure. Two-dimensional semiconductors have been proposed due to their promising device characteristics such as black phosphorus consisting of layer of phoshorene^10^. The study of single element materials such as P and As will give guidelines for the development of next-generation materials.

Single element arsenic minerals are well known for two polymorphs of crystalline phases named arsenic (space group $R\bar{{3}}m$) and arsenolamprite (space group *Bmab*). Semi-metallic grey arsenic ($R\bar{{3}}m$-As) is the most common and is believed to be the stable form of arsenic under ambient conditions. It is crystallized in a rhombohedral structure with space group $R\bar{{3}}m$ (A7-type structure). Black arsenic (black to dark-grey, *Bmab*-As) is glassy crystal and is isostructure with black phosphorous^11–13^. It can be prepared by cooling arsenic vapor at 100–220 °C or by heating amorphous As at 100–175 °C in the presence of Hg^14^. Black arsenic converts to semi-metallic grey arsenic at around 520 K. It is reported from theoretical calculations that pure black-arsenic is metastable and is stabilized by impurities^15^. The metastable yellow insulator arsenic consists of As_4_ molecules and has the same configuration as the P_4_ molecule. This allotrope is prepared by condensing the vapour on glass substrates at a very low temperature. This form is destroyed by X-ray radiation and transforms to semi-metallic grey arsenic at temperature above 30 K. No P-T phase diagram with crystalline forms has been proposed in the arsenic system^16^.

Arsenic has five valence electrons. Fundamentally, the number of bonds for covalent substances is 8-*N*, where *N* is the ordinal number of the Periodic Group, e.g. 8-5 = 3 for As. Each atom in arsenic polymorphs is in three-fold coordination as expected for the covalent bonding with electronic configuration *s*^2^*p*^3^. The *s*-orbital is fully occupied with two electrons and does not contribute to cohesion. The cohesion is dominated by the half-filled *p* orbitals. The group-V element As in its crystalline structure can be simply explained as the results of Peierls distortion of *p*-bonded atoms. The six lobes of the *p* orbitals lead to a simple cubic structure. Simple cubic to A7 distortion removes *p* orbital degeneracy and results in the formation of three saturated nearest-neighbor bonds. Doubling of the periodicity in the three principal directions of the simple cubic structure can be achieved by the alternation of short covalent bonds and long van der Waals bonds. This leads to a layer structure of tri-coordinated covalent atoms. The reason group V elements are semiconductors or semimetals is due to a gap at the Fermi level opened by the doubling of periodicity. The electron configuration of As is 4 *s*^2^ *3d*^10^ *4p*^3^, in which there are *d* orbitals in addition to *s* and *p* orbitals. Clarifying the contribution of *d* orbitals is important for understanding physical properties and subsequent material design.

### Crystal structure, morphology, twinning and comparison with other As phases

**Figure 1:**
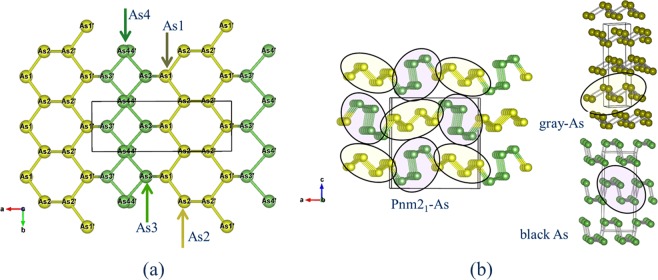
(**a**) Crystal structure of *Pnm2*_1_-type arsenic (pararsenolamprite) projected on (001) and (010). Each rectangle indicates the unit cell. (**b**) Comparison of crystal structures among crystalline arsenic phases. The structure of *Pnm2*_1_-As consists of 1:1 rod packing of grey- and black- arsenic parts. The array represents a herringbone pattern of two pieces. Grey arsenic ($R\bar{{3}}m$-As) has a layer structure and can be obtained by a Peiers-type distortion along [111] direction of a simple cubic structure^32,33^. In the structure, the six nearest neighbours in the original simple cubic structure splits into three equidistant nearest neighbours and three next nearest neighbours. Layered atoms form six-membered rings in chair conformation with intralayer and interlayer bond distances. The atomic arrangement in black arsenic (*Bmab*-As) consists of puckered layers stacked in the *c*-direction^13^.

The crystal structure of *Pnm2*_1_-type As was determined by single crystal X-ray diffraction method using superior experimental equipment. Since good single crystals could be found out and a good diffraction data set was obtained, the structure could be determined by the direct method (details are in the CIF file). The crystals are naturally occurring arsenic containing three percent antimony element of the homologous elements. Figure 1a,b show the crystal structure projected on (010) and (001), respectively. There are eight kinds of crystallographically non-equivalent As sites (Extended Data Table 1). All atoms are on the mirror plane parallel to (010). The As sites repeat as -As1-As2-As2′-As1′-As3′-As4′-As4-As3-As1- along a axis, or in opposite direction. The three-connected (buckled) six-gon layer bends at the As4′-As4 or As4-As4′ positions. Structurally, eight kinds of crystallographically equivalent As sites are divided into four pairs, As1-As1′, As2-As2′, As3-As3′ and As4-As4′. This structure can also be divided into two portions like As1-As2-As2′-As1′ (yellowish green sphere in Fig. 1a) and As3′-As4′-As4-As3 (green sphere).

Figure 1b shows a comparison of crystal structures among the crystalline arsenic phases (*Pnm2*_1_-As (pararsenolamprite), $R\bar{{3}}m$-As (semi-metallic grey native arsenic) and *Bmab*-As (black arsenic, arsenolampite)). It was revealed that the structure of *Pnm2*_1_-As, pararsenolamprite, is a mixed structure in which the structural units of grey- and black-As are regularly arranged. The structure of *Pnm2*_1_-As consists of 1:1 packing of $R\bar{{3}}m$-As and *Bmab*-As basic units. The array represents as a herringbone pattern consisting of two units.

The short covalent bond distances and angles in *Pnm2*_1_-As are presented in Extended Data Table 2. Bonding distances and angles are useful indicators of chemical bonding properties and orbitals of bonding electrons and allow comparisons among crystalline polymorphs. As with four pair positions can be divided into two groups with average bond angles greater (As3 (98.2°) and As4 (96.3°)) or smaller (As1′ (89.4°) and As2 (94.0°)) than the average value of 94.45°. The intralayer and interlayer As-As distances in *Pnm2*_1_-type As are intermediate between $R\bar{{3}}m$-type and *Bmab*-type As and have a spread in value.

**Figure 2:**
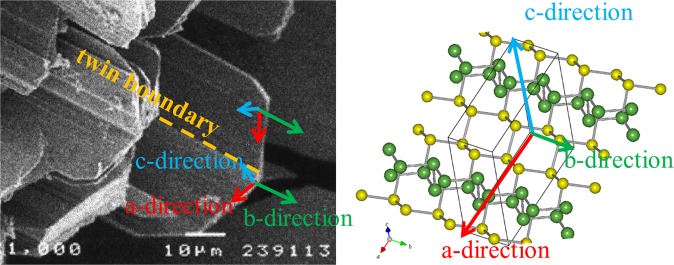
SEM image of crystal aggregate of *Pnm2*_1_-aresenic and the relationship between crystal morphology and lattice axis. Comparison of crystal morphology with unit lattice and atomic arrangement. *Pnm2*_1_-arsenic crystals form as bladed crystals, which show parallel growth along the b-axis. Poor growth in the direction perpendicular to the c-axis results in a thin plate shape. Because periodicity is short in the b-axis direction, *Pnm2*_1_-arsenic crystals tends to elongate in this direction, and the crystals assume a bladed shape.

The determined relationship between crystal morphology and unit cell axis for *Pnm2*_1_-As is shown in Fig. 2. A twin boundary and striations on the crystal surface are observed parallel to the b-axis. The structure of *Pnm2*_1_-As consists of a packing of $R\bar{{3}}m$*-* and *Bmab*-As portions (Fig. 1b). Both portions are parallel to the b-axis, and twinning and striations occur due to the change in the periodicity of the portions. A certain twin crystal will change the ratio of each $R\bar{{3}}m$*-* and *Bmab*-As portion.

### Structural optimization by simulation and electronic structure in *Pnm2*_1_-As

**Figure 3:**
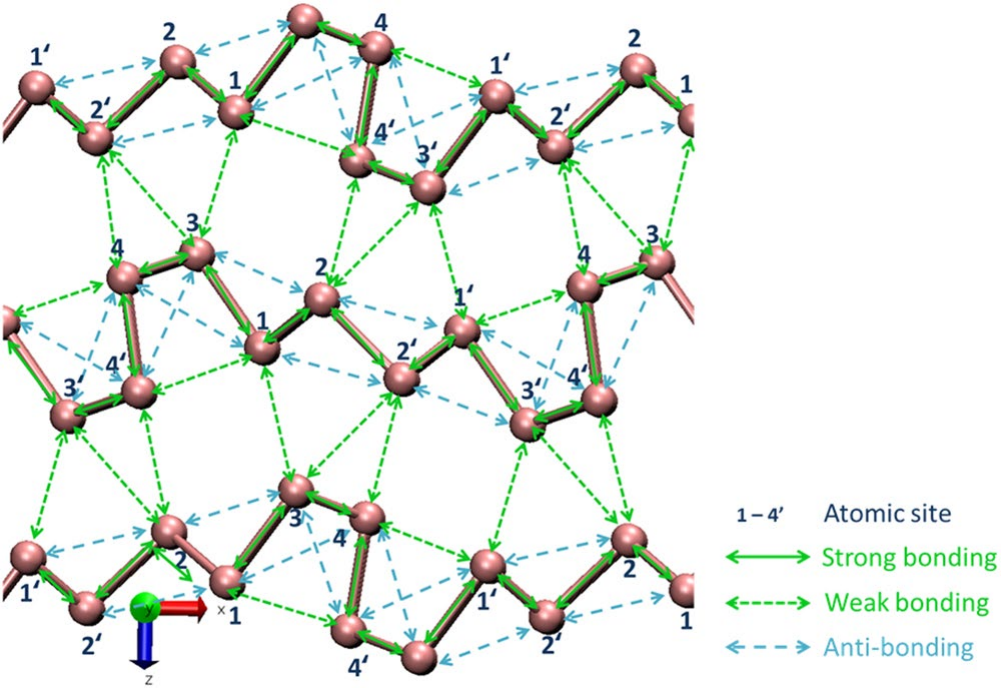
Covalent bonding (strong bonding), van der Waals bonding (weak bonding) and anti-bonding in *Pnm2*_1_-arsenic.

Experimentally determined unit cell constants, cell volume and atomic coordinates for *Pnm2*_1_-As were well optimized by the simulation. Differences between before and after optimization for each parameter were one percent or less. Covalent bonding (strong bonding), van der Waals bonding (weak bonding), and anti-bonding in *Pnm2*_1_-arsenic are shown in Fig. 3. A three-dimensional representation of bonds and anti-bonds around As1 atom in *Pnm2*_1_-As is indicated in Extended Data Fig. 1. Anti-bonding is observed only among atoms in each layer. Van der Waals bonding is observed in intralayer (As1…As4′ and As4…As1′) and the remaining ones are in interlayer (e.g. As1…As3, As2…As4′ and As2…As3′).

Partial orbital-orbital correlations of the bond overlap populations in *Pnm2*_1_-type arsenic crystal are summarized in Extended Data Table 2. For covalent bonding (strong bonding), the contribution of *pp* hybridization is the largest, and *pd* and *sd* have subsequent contributions. Bonding orbitals retain much of the *p*-like character. Contribution of *sp* hybridization was not observed. For van der Waals bonding (weak bonding), contributions of *sd* and *pd* hybridization are important, and *pp* has subsequent contributions. It is shown that *d*-orbitals contribute greatly in covalent bonds and especially in van der Waals bonds. The *d* electrons are also responsible for cohesion. Absolute values of partial orbital-orbital correlations for anti-bonding are usually larger than those for van der Waals bonding. Bond overlaps are particularly small in the As1-As3 and As2-As4 van der Waals bonds.

The electron configuration of As is 4 *s*^2^ *3d*^10^ *4p*^3^. Atomic net charge and contributions of each orbital to atomic charge in *Pnm2*_1_-As crystals are summarized in Table 1. The most important result is that total charges are clearly divided into positive and negative in the As1, As1′, As2, As2′ and As3, As3′, As4, As4′ portions, respectively. The deviation from 5.000 of the average charge of all atoms indicates electron transfer. Total charges of 4.97 for As2 and of 5.02 for As3 correspond to positive and negative charges of +0.03 and −0.02, respectively. The clear division of these net charges is caused by the difference in contribution of *d* orbitals. The contribution of *d* to the total charge is clearly less in As1, As1′, As2, As2′ portion corresponding to the $R\bar{{3}}m$*-*As structure part. The contribution of *s* + *p* in As1, As1′, As2, As2′ portion increases so as to compensate for the decrease in the contribution of *d*. The contribution of the *s* orbital is largest in As1As1′, and the contribution of *p* orbital is largest in As2As2′. Simulation results show unique electronic states in *Pnm2*_1_-As.

**Table 1:** Atomic net charge (Mulliken’s population of electrons) and contribution of each orbital to atomic charge in *Pnm2*_1_-type arsenic crystal.

	*s*	*p*	*d*	total
As1	1.86	2.87	0.26	4.99
As1′	1.86	2.87	0.26	4.99
As2	1.84	2.90	0.24	4.97
As2′	1.83	2.89	0.25	4.97
As3	1.84	2.87	0.31	5.02
As3′	1.84	2.87	0.31	5.02
As4	1.84	2.87	0.31	5.02
As4′	1.84	2.87	0.30	5.01
average	1.84	2.88	0.28	5.00

## Discussion

### Experimental information around Fermi level by XANES

**Figure 4:**
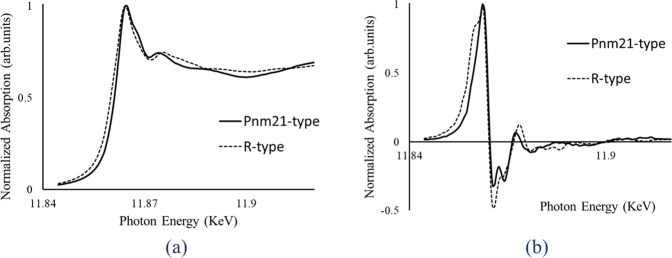
(**a**) Experimental As K-edge XANES spectra of *Pnm2*_1_-As (solid line) and $R\bar{{3}}m$-As (dashed line). (**b**) Differentiation of the XANSE curves (dXANES) is also shown.

Figure 4a compares observed XANES (X-ray absorption near edge structure) spectrum near the As K-edge for the *Pnm2*_1_-type arsenic to that of $R\bar{{3}}m$-type arsenic. These spectra are similar, but clear differences are detected. The energy at half-maximum height position (11.8611 keV) near the absorption edge for *Pnm2*_1_-type arsenic shifts by 1.2 eV to the higher energy side compared with 11.8599 keV for $R\bar{{3}}m$-type arsenic. This appears due to the electronic state change in the vicinity of the Fermi level and the increase in the partial cationic bonding characteristic (donating electrons) in the *Pnm2*_1_-type arsenic. The observed chemical shift of 1.2 eV in the XANES profile for *Pnm2*_1_-type may correspond to the simulation results (Table 1).

Details in XANES spectra can be examined more precisely by differential of XANES profile by photon energy (dXANES). Figure 4b shows dXANES profiles for *Pnm2*_1_-type and $R\bar{{3}}m$*-*type arsenic phases. The threshold energy of absorption edge for each element is usually defined at the maximum peak position in the dXANES profile. This is a position showing the maximum gradient at the absorption edge. Threshold energy (the absorption edge) shifts to the higher energy side with increasing arsenic oxidation state. However, both threshold energies found by the maximum values in dXANES spectrum (11.8623 keV) and the main peak position (11.8645 keV) in XANES spectra (Fig. 4a) show identical values in these phases, indicating that the chemical bonding as the sum of arsenic atoms in the *Pnm2*_1_-type and $R\bar{{3}}m$-type phases is the same.

### Structure control (with layer stacking and twin) and band gap

The discovered third crystalline form of arsenic (*Pnm2*_1_-type As, parasenolamprite) is a novel structure in which basic parts of $R\bar{{3}}m$- and *Bmab*-As are regularly arranged (Fig. 1b). Simulation results shows that the $R\bar{{3}}m$-part (As1As2 portion) is positively charged by releasing electrons and the *Bmab*-As part (As3As4 portion) is charged negatively by receiving electrons (Table 1). Net charges are clearly divided into positive and negative portions, Different characteristic parts coexist in a single element crystal. Each part continues indefinitely in parallel to the *b*-axis. Both parts are regularly arranged in the *a*-axial direction in the layer. Since the twin boundary runs parallel to the *b*-axis (Fig. 2), introduction of twin enables control of various types of basic partial periodicity inter and intra layer without changing the periodicity in the direction parallel to the b axis,.

The van der Waals bond distances between the adjacent layers are rich in variety from 3.12 Å in $R\bar{{3}}m$-As to 3.81 Å in *Bmab*-As. Contributions of *sd* and *pd* hybridization are most important for van der Waals bonding (Table 1). The van der Waals bonding distance and the contribution of the *d* orbitals change along with change in the stacking of layers in c-direction. This indicates that properties can be changed by control of layer stacking. The features of this structure are interesting to both material science and crystallography. Grey semi-metallic arsenic is an electric conductor, while black arsenic is a poor electric conductor. When grey semi-metal arsenic is amorphized, it becomes a semiconductor with a band gap of 1.2 to 1.4 eV^4^. The arsenic system allows creation of amorphous phases and some amorphous forms of arsenic exist. Discussions of amorphous-As have noted that physical properties change due to expansion of the interlayer spacing and additional disordering within the layers^13^. This corresponds well with our results, suggesting the importance of the contribution of *d* orbitals.

### Possibility for quantum materials: positively and negatively charged rods in two-dimensional structure

Several kinds of “organic superconductors” and “synthetic metals” were made by combining organic compounds or organic compounds with inorganic ions. Most were structures in which positively and negatively charged layers were stacked separately. The charge transfer complex called TTF:TCNQ with a column structure of tetrathiafulvalene (TTF) and tetracyanoquinodimethane (TCNQ) portions is known as a synthetic highly conductive donor-acceptor complex^17^. Discovery of the *Pnm2*_1_-As structure seems to be of some importance. Total charges are clearly divided into positive and negative in the same elements for the grey-As and black-As portions and the sequence in which one-dimensional electron donor and electron acceptor rods (parts) alternate in the layer is the first description. The ability to design chemical bonding and atomic arrangement within layers should open up further possibilities for the creation of new devices. Several phases of group-V elements show superconductivity as quantum physical properties^7–9^. Group V elements offer various possibilities to assemble new structures and control chemical bonding states and physical properties.

Unique physical characteristics can be expected not only as a three-dimensional structure but also as a two-dimensional structure. Phospholene as a single layer of layered structure has been proposed as a promising device^10^. Semi-metallic $R\bar{{3}}m$-phosphorous and black *Bmab* (*Cmca*)-phosphorus have structures similar to grey-As and black-As, respectively. Phosphorus, an atom in the third period, also uses the *d*-orbital in some cases depending on interactions with the electron shell of the binding partner. The *3d* orbital in phosphorus, in addition to *3* *s* and *3p* orbitals, may also take part in the bonding of crystalline forms by constructing hybridized orbitals. Regarding phosphorus and antimony, we would like to mention the possibility of controlling arrays in the layer with two kinds of portions similar to the *Pnm2*_1_-As phase. The possibility of two-dimensional materials in which one-dimensional electron donors and electron acceptor rods alternate in a layer may be expected as a quantum material.

## Methods

### Sample and chemical composition

Pararsenolamprite, the third natural crystalline polymorph of As, was found in a dump of hydrothermal ore deposits of the Mukuno mine, Oita, Japan^1^. We found suitable pararsenolamprite crystals for single crystal structure analysis in a specimen of parallel aggregates of bladed crystals from a National Science Museum sample (sample No.NSM-28015). The crystals are lead-like dark grey in colour and opaque with metallic lustre. The crystal samples have clear crystal faces and perfect cleavage. Pararsenolamprite is sectile and brittle with perfect cleavage on [001]. Euhedral crystals elongated on [010] and flattened on (001). A pararsenolamprite single crystal of dimensions 27 × 39 × 17 µm was cut and removed from euhedral crystal aggregate for use with single-crystal structure analysis. Its chemical composition of As_0.97_Sb_0.03_ was determined by JEOL scanning electron microscope SEM JSM-7001F and Oxford energy dispersive X-ray analyser EDS INCA SYSTEM. This value is consistent with those reported by Matsubara *et al*.^1^. The arsenic crystal of this mine is characterized by including small amounts of Sb.

### Crystal structure analysis

Space group orthorhombic *Pnm2*_1_ and lattice constants, a = 10.1193(7), b = 3.6288(2), c = 10.3152 (10) Å were determined by RIGAKU XtaLAB SuperNova using graphite-monochromatized MoKα radiations and a four-circle diffractometer using synchrotron radiation at beamline BL-10A of PF, KEK^18,19^. Each crystallographic datum is shown in Extended data Table 1. A total of 3371 reflections were collected, and the data were corrected for absorption effect and Lorentz and polarization factors. After initial structures were solved by direct method, 742 unique reflections were used for refinement with |*F*_o_| ≥ 4σ(|*F*_o_|) by full matrix-least-square method. The structure was determined with the ShelXT solution program using Intrinsic Phasing and refined with the ShelXL refinement package using least-squares minimisation^20^. After the least-square refinements, each *R* index (=Σ||Fo| - |Fc||/Σ|Fo|) was convergence of 0.0575 using isotropic temperature factors. Positional parameters and isotropic thermal displacement parameters are given in Extended data Table 2. The crystal structure was illustrated using VESTA^21^.

### XANES measurements

X-ray absorption near edge-structure (XANES) spectroscopy is effective for electro structure characterisations. Spectra near the As K-edge for *Pnm2*_1_-type and $R\bar{{3}}m$-type arsenic were collected in fluorescence mode using a Lytle-type detector. Measurements were performed with a Si(111) double crystal monochromater at BL-9C blanch lines of the Photon Factory at the High Energy Accelerator Research Organization (KEK), Tsukuba, Japan. Mirrors were used to eliminate higher harmonics. X-ray energy calibration was performed by setting the copper metal pre-edge absorption peak to 8978.8 eV. Details of the measurements and analyses are given by Yoshiasa *et al*.^22,23^ and Sakai *et al*.^24^. Figure 4a,b compare the observed XANES spectrum near the As K-edge and the differential of XANES profile by photon energy (dXANES) for *Pnm2*_1_-type arsenic with those for $R\bar{{3}}m$-type arsenic.

### First principles calculations

Theoretical works have been performed to understand bonding characteristics of *Pnm2*_1_-type arsenic based on the first-principles computational method^25^. Electronic states were calculated by the projector augmented-wave (PAW) method within the framework of density functional theory (DFT)^26^. The generalized gradient approximation (GGA) functional proposed by Perdew, Burke and Ernzerhof^27^ was employed for the exchange-correlation energy. The DFT-D method was employed for semiempirical correction of the van der Waals interaction^28^. The momentum-space formalism was utilized, where the plane-wave cutoff energies are 11.0 and 70.0 Ry for the electronic pseudo-wave function and the pseudo-charge density, respectively^29^. The energy was minimized with respect to the Kohn-Sham orbitals iteratively using a preconditioned conjugate-gradient method^30^. Projector functions were generated for the 4 *s*, 4*p* and 4*d* states as the valence states for arsenic atoms. Periodic boundary conditions were applied to all Cartesian directions. Structural optimization was performed using the experimentally determined crystallographic data of the orthorhombic unit cell containing 16 atoms, which are listed in Extended Data Table 4. The optimized unit cell constants (Å) were a = 10.1594, b = 3.6649 and c = 10.3882. Monkhorst-Pack grids of 2 × 6 × 2 *k* points were used for Brillouin zone sampling. We obtained the minimum-energy atomic configuration under 0.0 GPa using the quasi-Newton method. Calculated lattice constants and atomic coordinates are in good agreement with our experimental results. The atomic net charge of each atom and bond overlap populations between each atomic pair were calculated based on the population analysis method^31^.

## Supplementary information

Extended Dataset
