# Supplementary material for: Natural arsenic with a unique order structure: potential for new quantum materials
Source: Sci Rep. 2019 Apr 18;9:6275. doi: 10.1038/s41598-019-42561-8 (PMC6472341; doi:10.1038/s41598-019-42561-8)
Supplement: Supplementary file 1 — Extended Dataset [file 41598_2019_42561_MOESM1_ESM.doc]

Natural arsenic with a unique order structure: potential for new quantum materials

Akira Yoshiasa, Makoto Tokuda, Masaaki Misawa, Fuyuki Shimojo, Koichi Momma, Ritsuro Miyawaki, Satoshi Matsubara, Akihiko Nakatsuka and Kazumasa Sugiyama,

*****************************

Extended Data Table 1. X-ray data for pararsenolamprite (*Pnm2_1_*-type arsenic crystal).

a (Å) 10.1193(7)

b (Å) 3.6288(2)

c (Å) 10.3152(10)

V (Å^3^) 378.78(5)

Z 16

d_calc_ (g/cm^3^)　 5.28

Crystal size (mm) 0.027 x 0.039 x 0.017

Diffractometer RIGAKU XtaLAB SuperNova

radiation used MoKα (0.71073 Å)

Scan type ω

2θ range (°) 5.6 - 54.9

Range of hkl -13<h<13, -4<k<4, -13<l<13

No. of reflec. measured 3371

No. of indep. reflec.

used for refinement 743 (|Fo|˃4σ|Fo|)

R1 0.0545

wR2R 0.1459

GooF, S 1.093

μ (cm-1) 347.2

F000 528

Extended Data Table 2. Experimentally determined unit cell constants (Å), atomic coordinates and equivalent temperature factors (Å^2^) on *Pnm2_1_*-As.

( unit cell data a= 10.1193(7) b= 3.6288(2) c= 10.3152 (10) )

atom x y z Ueq

As1 0.4702(5) 0.5 0.2348(7) 0.0164(16)

As1’ 0.0351(6) 0.5 0.2767(7) 0.0192(16)

As2 0.3399(6) 0.0 0.3394(7) 0.0214(17)

As2’ 0.1660(6) 0.0 0.1718(6) 0.0165(15)

As3 0.6140 (6) 0.5 0.4318(6) 0.0228(16)

As3’ 0.8914 (5) 0.5 0.0788 (5) 0.0121(12)

As4 0.7720(6) 0.0 0.3746(7) 0.0236(16)

As4’ 0.7333(5) 0.0 0.1377(6) 0.0130(13)

Extended Data Table 3. Selected strong bonding distances (Å) and angles (°) in *Pnm2_1_-*As *. The As-As interlayer distances between atoms in different layers take 3.329(9) and 3.713(9) Å**.

As1 - As2 x2 2.490(6) As2-As1-As2 93.6(3) As1’ - As2’ x2 2.493(7) As2’-As1’-As2’ 93.4 (3)

- As3 2.500(10) As2-As1-As3 x2 87.5(3) - As3’ 2.506(10) As2’-As1’-As’3x2 87.4 (3)

Av. 2.493 89.5 Av. 2.497 89.4

As2 - As1 x2 2.490(6) As1-As2-As1 93.6(3) As2’ - As1’ x2 2.493(7) As1’-As2’-As1’ 93.4(3)

- As2’ 2.467(5) As1-As2-As2’ x2 94.2 (2) - As2 2.467(5) As1’-As2’-As2’x2 94.3(2)

Av. 2.482 94.00 Av. 2.484 94.00

As3 - As4 x2 2.489(7) As4-As3-As4 93.6(3) As3’ - As4’ x2 2.494(6) As4’-As3’-As4’ 93.3(3)

- As1 2.500(10) As1-As3-As4 x2 100.5(3) - As1’ 2.506(10) As1’-As3’-As4’ x2 100.0(3)

Av, 2.493 98.2 Av, 2.498 97.8

As4 - As3 x2 2.489(7) As3-As4-As3 93.6(3) As4’ - As3’ x2 2.494(6) As3’-As4’-As3’ 93.3(3)

- As4’ 2.475(5) As3-As4-As4’ x2 97.6(2) - As4 2.475(5) As3’-As4’-As4 x2 98.0 (2)

Av. 2.484 96.3 Av. 2.488 96.4

* Each atom in the layer is covalently bonded to three neighbours with one bond length of 2.467(5) - 2.506 (10) Å in the mirror plane (e.g. As1-As3) and the other equivalent two bonds of length 2.489 (7) - 2.494(6) Å (e.g. As1-As2 ×2). The bond angles involving mirror symmetry such as As2-As1-As2 (angles of 93.3(3)° -93.6(3)°) display fewer variations than other inequivalent two bond angles such as As3-As1-As2 ×2 (angles of 87.5(3)° - 100.5(3)°).

** In *Rm*-As, the intralayer distances is 2.517 Å (Schiferl and Barrett, 1969). The angle between covalent bonds in the layer plane is 96.64°. The interlayer distance between atoms is 3.120 Å. In *Bmab*-As, Interatomic distances within layer are 2.48 and 2.49 Å with the bond angles of 94.1° and 98.5°. Those across the adjacent layers are 3.62 and 3.81 Å.

Extended Data Table 4. Partial orbital-orbital correlations of the bond overlap populations of atoms in *Pnm2_1_*-type arsenic crystal.

Mulliken's overlap populations around As1.

|  | *s-s* | *s-p* | *s-d* | *p-s* | *p-p* | *p-d* | *d-s* | *d-p* | *d-d* | total |
| --- | --- | --- | --- | --- | --- | --- | --- | --- | --- | --- |
| As1 | 0.00 | -0.02 | -0.02 | -0.02 | -0.04 | -0.01 | -0.02 | -0.01 | -0.04 | -0.16 |
| As2 | -0.03 | -0.08 | 0.05 | -0.06 | 0.45 | 0.12 | 0.05 | 0.10 | -0.02 | 0.59 |
| As2' | 0.00 | -0.02 | -0.02 | -0.02 | -0.04 | 0.00 | -0.03 | -0.01 | -0.03 | -0.18 |
| As3(1) | -0.02 | -0.07 | 0.04 | -0.10 | 0.42 | 0.10 | 0.05 | 0.08 | 0.00 | 0.49 |
| As3(2) | 0.00 | -0.01 | 0.03 | -0.01 | 0.01 | 0.03 | 0.04 | 0.03 | -0.03 | 0.08 |
| As4 | 0.00 | -0.01 | -0.03 | 0.01 | -0.06 | -0.01 | -0.02 | -0.02 | -0.03 | -0.17 |
| As4' | -0.01 | -0.01 | 0.04 | -0.01 | 0.01 | 0.04 | 0.05 | 0.04 | -0.01 | 0.13 |

Mulliken's overlap populations around As1'.

|  | *s-s* | *s-p* | *s-d* | *p-s* | *p-p* | *p-d* | *d-s* | *d-p* | *d-d* | total |
| --- | --- | --- | --- | --- | --- | --- | --- | --- | --- | --- |
| As1' | 0.00 | -0.02 | -0.02 | -0.02 | -0.04 | -0.01 | -0.02 | -0.01 | -0.04 | -0.16 |
| As2 | 0.00 | -0.02 | -0.02 | -0.02 | -0.04 | 0.00 | -0.03 | -0.01 | -0.03 | -0.18 |
| As2' | -0.03 | -0.08 | 0.05 | -0.06 | 0.45 | 0.12 | 0.05 | 0.10 | -0.02 | 0.59 |
| As3'(1) | -0.02 | -0.07 | 0.03 | -0.10 | 0.42 | 0.10 | 0.05 | 0.08 | -0.01 | 0.49 |
| As3'(2) | 0.00 | -0.01 | 0.03 | -0.01 | 0.01 | 0.03 | 0.04 | 0.03 | -0.03 | 0.09 |
| As4 | -0.01 | -0.01 | 0.04 | -0.01 | 0.01 | 0.04 | 0.05 | 0.04 | 0.00 | 0.13 |
| As4' | 0.00 | -0.01 | -0.03 | 0.01 | -0.06 | -0.01 | -0.02 | -0.02 | -0.03 | -0.17 |

Mulliken's overlap populations around As2.

|  | *s-s* | *s-p* | *s-d* | *p-s* | *p-p* | *p-d* | *d-s* | *d-p* | *d-d* | total |
| --- | --- | --- | --- | --- | --- | --- | --- | --- | --- | --- |
| As1 | -0.03 | -0.06 | 0.05 | -0.08 | 0.45 | 0.10 | 0.05 | 0.12 | -0.02 | 0.59 |
| As1' | 0.00 | -0.02 | -0.03 | -0.02 | -0.04 | -0.01 | -0.02 | 0.00 | -0.03 | -0.18 |
| As2 | 0.00 | -0.02 | -0.02 | -0.02 | -0.04 | -0.01 | -0.02 | -0.01 | -0.03 | -0.16 |
| As2' | -0.02 | -0.07 | 0.06 | -0.07 | 0.46 | 0.10 | 0.06 | 0.10 | -0.02 | 0.61 |
| As3 | 0.00 | -0.03 | -0.02 | -0.03 | -0.03 | 0.01 | -0.01 | 0.00 | -0.04 | -0.14 |
| As3' | -0.01 | -0.01 | 0.08 | -0.02 | 0.01 | 0.04 | 0.01 | 0.03 | 0.01 | 0.13 |
| As4' | 0.00 | -0.01 | 0.03 | -0.01 | 0.01 | 0.02 | 0.01 | 0.03 | -0.01 | 0.05 |

Mulliken's overlap populations around As2'.

|  | *s-s* | *s-p* | *s-d* | *p-s* | *p-p* | *p-d* | *d-s* | *d-p* | *d-d* | total |
| --- | --- | --- | --- | --- | --- | --- | --- | --- | --- | --- |
| As1 | 0.00 | -0.02 | -0.03 | -0.02 | -0.04 | -0.01 | -0.02 | 0.00 | -0.03 | -0.18 |
| As1' | -0.03 | -0.06 | 0.05 | -0.08 | 0.45 | 0.10 | 0.05 | 0.12 | -0.02 | 0.59 |
| As2 | -0.02 | -0.07 | 0.06 | -0.07 | 0.46 | 0.10 | 0.06 | 0.10 | -0.02 | 0.61 |
| As2' | 0.00 | -0.02 | -0.02 | -0.02 | -0.04 | -0.01 | -0.02 | -0.01 | -0.03 | -0.16 |
| As3 | -0.01 | -0.02 | 0.09 | -0.03 | 0.02 | 0.04 | 0.01 | 0.04 | 0.01 | 0.16 |
| As3' | 0.00 | -0.03 | -0.02 | -0.02 | -0.03 | 0.01 | -0.02 | 0.00 | -0.03 | -0.14 |
| As4 | 0.00 | -0.01 | 0.03 | -0.01 | 0.01 | 0.02 | 0.01 | 0.03 | -0.01 | 0.05 |

Mulliken's overlap populations around As3.

|  | *s-s* | *s-p* | *s-d* | *p-s* | *p-p* | *p-d* | *d-s* | *d-p* | *d-d* | total |
| --- | --- | --- | --- | --- | --- | --- | --- | --- | --- | --- |
| As1(1) | -0.02 | -0.10 | 0.05 | -0.07 | 0.42 | 0.08 | 0.04 | 0.10 | 0.00 | 0.49 |
| As1(2) | 0.00 | -0.01 | 0.04 | -0.01 | 0.01 | 0.03 | 0.03 | 0.03 | -0.03 | 0.08 |
| As2 | 0.00 | -0.03 | -0.01 | -0.03 | -0.03 | 0.00 | -0.02 | 0.01 | -0.04 | -0.14 |
| As2' | -0.01 | -0.03 | 0.01 | -0.02 | 0.02 | 0.04 | 0.09 | 0.04 | 0.01 | 0.16 |
| As3 | 0.00 | -0.02 | -0.02 | -0.02 | -0.04 | -0.01 | -0.02 | -0.01 | -0.04 | -0.16 |
| As4 | -0.04 | -0.06 | 0.07 | -0.06 | 0.45 | 0.12 | 0.06 | 0.13 | -0.01 | 0.66 |
| As4' | 0.00 | 0.01 | -0.04 | -0.02 | -0.07 | 0.00 | -0.04 | -0.01 | -0.02 | -0.19 |

Mulliken's overlap populations around As3'.

|  | *s-s* | *s-p* | *s-d* | *p-s* | *p-p* | *p-d* | *d-s* | *d-p* | *d-d* | total |
| --- | --- | --- | --- | --- | --- | --- | --- | --- | --- | --- |
| As1'(1) | -0.02 | -0.10 | 0.05 | -0.07 | 0.42 | 0.08 | 0.03 | 0.10 | -0.01 | 0.49 |
| As1'(2) | 0.00 | -0.01 | 0.04 | -0.01 | 0.01 | 0.03 | 0.03 | 0.03 | -0.03 | 0.09 |
| As2 | -0.01 | -0.02 | 0.01 | -0.01 | 0.01 | 0.03 | 0.08 | 0.04 | 0.01 | 0.13 |
| As2' | 0.00 | -0.02 | -0.02 | -0.03 | -0.03 | 0.00 | -0.02 | 0.01 | -0.03 | -0.14 |
| As3' | 0.00 | -0.02 | -0.02 | -0.02 | -0.04 | -0.01 | -0.02 | -0.01 | -0.04 | -0.16 |
| As4 | 0.00 | 0.01 | -0.04 | -0.02 | -0.07 | 0.00 | -0.04 | -0.01 | -0.02 | -0.18 |
| As4' | -0.04 | -0.05 | 0.07 | -0.06 | 0.45 | 0.12 | 0.05 | 0.13 | -0.01 | 0.66 |

Mulliken's overlap populations around As4.

|  | *s-s* | *s-p* | *s-d* | *p-s* | *p-p* | *p-d* | *d-s* | *d-p* | *d-d* | total |
| --- | --- | --- | --- | --- | --- | --- | --- | --- | --- | --- |
| As1 | 0.00 | 0.01 | -0.02 | -0.01 | -0.06 | -0.02 | -0.03 | -0.01 | -0.03 | -0.17 |
| As1' | -0.01 | -0.01 | 0.05 | -0.01 | 0.01 | 0.04 | 0.04 | 0.04 | 0.00 | 0.13 |
| As2' | 0.00 | -0.01 | 0.01 | -0.01 | 0.01 | 0.03 | 0.03 | 0.02 | -0.01 | 0.05 |
| As3 | -0.04 | -0.06 | 0.06 | -0.06 | 0.45 | 0.13 | 0.07 | 0.12 | -0.01 | 0.66 |
| As3' | 0.00 | -0.02 | -0.04 | 0.01 | -0.07 | -0.01 | -0.04 | 0.00 | -0.02 | -0.18 |
| As4 | 0.00 | -0.02 | -0.02 | -0.02 | -0.04 | -0.01 | -0.02 | -0.01 | -0.03 | -0.15 |
| As4' | -0.02 | -0.07 | 0.06 | -0.07 | 0.44 | 0.10 | 0.06 | 0.10 | -0.03 | 0.56 |

Mulliken's overlap populations around As4'.

|  | *s-s* | *s-p* | *s-d* | *p-s* | *p-p* | *p-d* | *d-s* | *d-p* | *d-d* | total |
| --- | --- | --- | --- | --- | --- | --- | --- | --- | --- | --- |
| As1 | -0.01 | -0.01 | 0.05 | -0.01 | 0.01 | 0.04 | 0.04 | 0.04 | -0.01 | 0.13 |
| As1' | 0.00 | 0.01 | -0.02 | -0.01 | -0.06 | -0.02 | -0.03 | -0.01 | -0.03 | -0.17 |
| As2 | 0.00 | -0.01 | 0.01 | -0.01 | 0.01 | 0.03 | 0.03 | 0.02 | -0.01 | 0.05 |
| As3 | 0.00 | -0.02 | -0.04 | 0.01 | -0.07 | -0.01 | -0.04 | 0.00 | -0.02 | -0.19 |
| As3' | -0.04 | -0.06 | 0.05 | -0.05 | 0.45 | 0.13 | 0.07 | 0.12 | -0.01 | 0.66 |
| As4 | -0.02 | -0.07 | 0.06 | -0.07 | 0.44 | 0.10 | 0.06 | 0.10 | -0.03 | 0.56 |
| As4' | 0.00 | -0.02 | -0.02 | -0.02 | -0.04 | -0.01 | -0.02 | -0.01 | -0.03 | -0.15 |


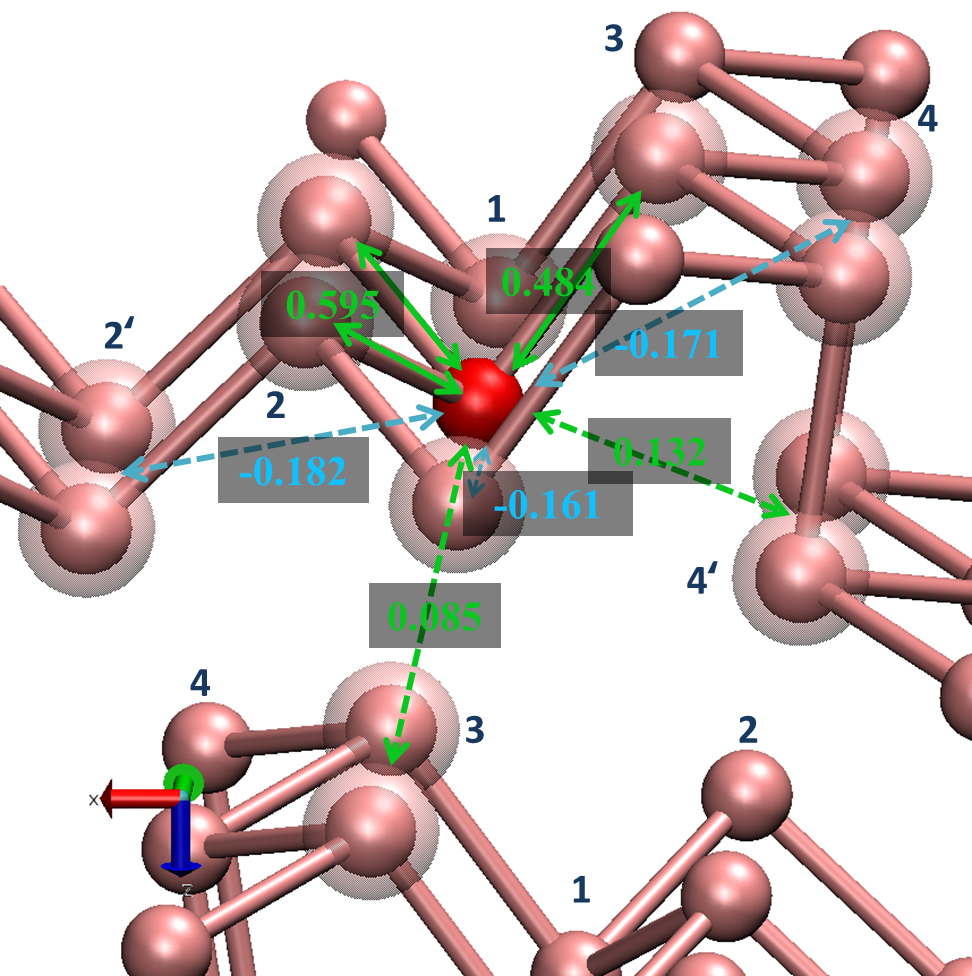


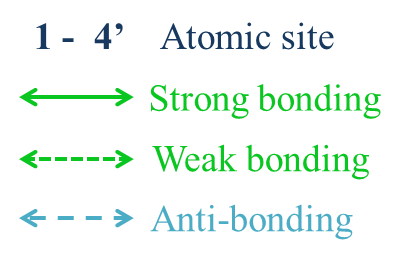


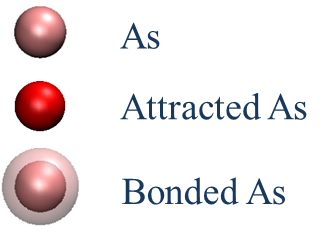


Extended Data Figure 1. Three-dimensional representation of bonds and anti-bonds around As1 atom in *Pnm2_1_*-arsenic.

********************************************************

Comment on Check CIF file, Alert level B

Alert level B

PLAT111_ALERT_2_B ADDSYM Detects New (Pseudo) Centre of Symmetry. 100 %Fit PLAT112_ALERT_2_B ADDSYM Detects New (Pseudo) Symm. Elem 100 %Fit PLAT113_ALERT_2_B ADDSYM Suggests Possible Pseudo/New Space Group Pnnm Check

→ We also analyzed the Pnmn (Pnnm) model. The R1 factor in the Pnmn (Pnnm) model was 0.0763. Since this R value is dominantly larger than the R value in the P21nm (Pnm21) model, we adopt the P21nm (Pnm21) model with R1=0.0545. The refined atomic coordinates are in good agreement within the error in both models. No further refinement was possible with this sample, which is a natural product.

Alert level B

PLAT097_ALERT_2_B Large Reported Max. (Positive) Residual Density 3.36 eA-3 PLAT971_ALERT_2_B Check Calcd Resid. Dens. 2.01A From As3 2.74 eA-3 PLAT971_ALERT_2_B Check Calcd Resid. Dens. 2.13A From As3 2.53 eA-3 PLAT987_ALERT_1_B The Flack x is >> 0 - Do a BASF/TWIN Refinement Please Check

→ No further refinement was possible with this crystal sample, which is a natural product, though we tried to search for good quality crystals. The R1 value (0.0545) is low and there is no problem with the discussion of symmetry of structure and atomic position.

CifFile AsnewStructure

data_shelx

_audit_creation_method 'SHELXL-2016/6'

_shelx_SHELXL_version_number '2016/6'

_chemical_name_systematic ?

_chemical_name_common ?

_chemical_melting_point ?

_chemical_formula_moiety ?

_chemical_formula_sum

'As2'

_chemical_formula_weight 149.84

loop_

_atom_type_symbol

_atom_type_description

_atom_type_scat_dispersion_real

_atom_type_scat_dispersion_imag

_atom_type_scat_source

'As' 'As' 0.0499 2.0058

'International Tables Vol C Tables 4.2.6.8 and 6.1.1.4'

_space_group_crystal_system 'orthorhombic'

_space_group_IT_number 31

_space_group_name_H-M_alt 'P n m 21'

_space_group_name_Hall 'P 2bc -2bc'

_shelx_space_group_comment

;

The symmetry employed for this shelxl refinement is uniquely defined

by the following loop, which should always be used as a source of

symmetry information in preference to the above space-group names.

They are only intended as comments.

;

loop_

_space_group_symop_operation_xyz

'x, y, z'

'-x, -y+1/2, z+1/2'

'-x, y+1/2, z+1/2'

'x, -y, z'

_cell_length_a 10.1193(7)

_cell_length_b 3.6288(2)

_cell_length_c 10.3152(10)

_cell_angle_alpha 90

_cell_angle_beta 90

_cell_angle_gamma 90

_cell_volume 378.78(5)

_cell_formula_units_Z 8

_cell_measurement_temperature 293(2)

_cell_measurement_reflns_used 1492

_cell_measurement_theta_min 3.9360

_cell_measurement_theta_max 30.5230

_exptl_crystal_description Plate

_exptl_crystal_colour metallic

_exptl_crystal_density_meas ?

_exptl_crystal_density_method ?

_exptl_crystal_density_diffrn 5.255

_exptl_crystal_F_000 528

_exptl_transmission_factor_min ?

_exptl_transmission_factor_max ?

_exptl_crystal_size_max 0.092

_exptl_crystal_size_mid 0.031

_exptl_crystal_size_min 0.012

_exptl_absorpt_coefficient_mu 34.722

_shelx_estimated_absorpt_T_min ?

_shelx_estimated_absorpt_T_max ?

_exptl_absorpt_correction_type gaussian

_exptl_absorpt_correction_T_min 0.238

_exptl_absorpt_correction_T_max 0.733

_exptl_absorpt_process_details ?

_exptl_absorpt_special_details ?

_diffrn_ambient_temperature 293(2)

_diffrn_radiation_wavelength 0.71073

_diffrn_radiation_type MoK\a

_diffrn_source ?

_diffrn_measurement_device 'four-circle diffractometer'

_diffrn_measurement_device_type 'SuperNova, Single source at offset/far, HyPix3000'

_diffrn_measurement_method '\w scans'

_diffrn_detector_area_resol_mean ?

_diffrn_reflns_number 3367

_diffrn_reflns_av_unetI/netI 0.0491

_diffrn_reflns_av_R_equivalents 0.0455

_diffrn_reflns_limit_h_min -13

_diffrn_reflns_limit_h_max 13

_diffrn_reflns_limit_k_min -4

_diffrn_reflns_limit_k_max 4

_diffrn_reflns_limit_l_min -13

_diffrn_reflns_limit_l_max 13

_diffrn_reflns_theta_min 2.013

_diffrn_reflns_theta_max 27.497

_diffrn_reflns_theta_full 25.242

_diffrn_measured_fraction_theta_max 1.000

_diffrn_measured_fraction_theta_full 1.000

_diffrn_reflns_Laue_measured_fraction_max 1.000

_diffrn_reflns_Laue_measured_fraction_full 1.000

_diffrn_reflns_point_group_measured_fraction_max 0.931

_diffrn_reflns_point_group_measured_fraction_full 0.953

_reflns_number_total 935

_reflns_number_gt 743

_reflns_threshold_expression 'I > 2\s(I)'

_reflns_Friedel_coverage 0.767

_reflns_Friedel_fraction_max 0.855

_reflns_Friedel_fraction_full 0.899

_reflns_special_details

;

Reflections were merged by SHELXL according to the crystal

class for the calculation of statistics and refinement.

_reflns_Friedel_fraction is defined as the number of unique

Friedel pairs measured divided by the number that would be

possible theoretically, ignoring centric projections and

systematic absences.

;

_computing_cell_refinement 'CrysAlisPro 1.171.39.32e (Rigaku OD, 2017)'

_computing_data_collection 'CrysAlisPro 1.171.39.32e (Rigaku OD, 2017)'

_computing_data_reduction 'CrysAlisPro 1.171.39.32e (Rigaku OD, 2017)'

_computing_structure_solution ?

_computing_structure_refinement 'SHELXL-2016/6 (Sheldrick, 2016)'

_computing_molecular_graphics ?

_computing_publication_material ?

_refine_special_details ?

_refine_ls_structure_factor_coef Fsqd

_refine_ls_matrix_type full

_refine_ls_weighting_scheme calc

_refine_ls_weighting_details

'w=1/[\s^2^(Fo^2^)+(0.0890P)^2^+0.5830P] where P=(Fo^2^+2Fc^2^)/3'

_atom_sites_solution_primary ?

_atom_sites_solution_secondary ?

_atom_sites_solution_hydrogens .

_refine_ls_hydrogen_treatment undef

_refine_ls_extinction_method none

_refine_ls_extinction_coef .

_refine_ls_abs_structure_details

;

Flack x determined using 278 quotients [(I+)-(I-)]/[(I+)+(I-)]

(Parsons, Flack and Wagner, Acta Cryst. B69 (2013) 249-259).

;

_refine_ls_abs_structure_Flack 10.0(10)

_chemical_absolute_configuration ?

_refine_ls_number_reflns 935

_refine_ls_number_parameters 49

_refine_ls_number_restraints 1

_refine_ls_R_factor_all 0.0718

_refine_ls_R_factor_gt 0.0545

_refine_ls_wR_factor_ref 0.1459

_refine_ls_wR_factor_gt 0.1379

_refine_ls_goodness_of_fit_ref 1.093

_refine_ls_restrained_S_all 1.092

_refine_ls_shift/su_max 0.000

_refine_ls_shift/su_mean 0.000

loop_

_atom_site_label

_atom_site_type_symbol

_atom_site_fract_x

_atom_site_fract_y

_atom_site_fract_z

_atom_site_U_iso_or_equiv

_atom_site_adp_type

_atom_site_occupancy

_atom_site_site_symmetry_order

_atom_site_calc_flag

_atom_site_refinement_flags_posn

_atom_site_refinement_flags_adp

_atom_site_refinement_flags_occupancy

_atom_site_disorder_assembly

_atom_site_disorder_group

As1 As 0.4707(5) 1.500000 -0.7657(5) 0.0133(13) Uani 1 2 d S T P . .

As1' As 0.0353(5) 0.500000 -0.7243(7) 0.0222(17) Uani 1 2 d S T P . .

As2 As 0.3400(6) 1.000000 -0.6604(6) 0.0206(16) Uani 1 2 d S T P . .

As2' As 0.1659(5) 1.000000 -0.8284(5) 0.0176(15) Uani 1 2 d S T P . .

As3 As 0.6139(5) 1.500000 -0.5669(5) 0.0147(13) Uani 1 2 d S T P . .

As3' As -0.1088(5) 0.500000 -0.9198(6) 0.0203(14) Uani 1 2 d S T P . .

As4 As -0.2275(5) 0.000000 -0.6264(6) 0.0160(13) Uani 1 2 d S T P . .

As4' As -0.2661(5) 0.000000 -0.8632(6) 0.0212(14) Uani 1 2 d S T P . .

loop_

_atom_site_aniso_label

_atom_site_aniso_U_11

_atom_site_aniso_U_22

_atom_site_aniso_U_33

_atom_site_aniso_U_23

_atom_site_aniso_U_13

_atom_site_aniso_U_12

As1 0.011(2) 0.009(3) 0.020(3) 0.000 -0.007(2) 0.000

As1' 0.009(2) 0.015(4) 0.043(4) 0.000 0.005(2) 0.000

As2 0.019(3) 0.005(2) 0.038(4) 0.000 0.002(3) 0.000

As2' 0.0091(19) 0.019(3) 0.024(4) 0.000 -0.001(2) 0.000

As3 0.025(3) 0.008(3) 0.011(2) 0.000 0.000(2) 0.000

As3' 0.009(2) 0.013(3) 0.038(4) 0.000 0.000(2) 0.000

As4 0.015(2) 0.002(2) 0.031(4) 0.000 0.000(2) 0.000

As4' 0.0100(19) 0.024(4) 0.029(3) 0.000 -0.003(3) 0.000

_geom_special_details

;

All esds (except the esd in the dihedral angle between two l.s. planes)

are estimated using the full covariance matrix. The cell esds are taken

into account individually in the estimation of esds in distances, angles

and torsion angles; correlations between esds in cell parameters are only

used when they are defined by crystal symmetry. An approximate (isotropic)

treatment of cell esds is used for estimating esds involving l.s. planes.

;

loop_

_geom_bond_atom_site_label_1

_geom_bond_atom_site_label_2

_geom_bond_distance

_geom_bond_site_symmetry_2

_geom_bond_publ_flag

As1 As2 2.494(6) . ?

As1 As2 2.494(6) 1_565 ?

As1 As3 2.512(8) . ?

As1' As3' 2.489(9) . ?

As1' As2' 2.489(7) 1_545 ?

As1' As2' 2.489(7) . ?

As2 As2' 2.471(4) . ?

As3 As4 2.499(6) 1_675 ?

As3 As4 2.499(6) 1_665 ?

As3' As4' 2.484(6) 1_565 ?

As3' As4' 2.484(6) . ?

As4 As4' 2.474(4) . ?

loop_

_geom_angle_atom_site_label_1

_geom_angle_atom_site_label_2

_geom_angle_atom_site_label_3

_geom_angle

_geom_angle_site_symmetry_1

_geom_angle_site_symmetry_3

_geom_angle_publ_flag

As2 As1 As2 93.3(3) . 1_565 ?

As2 As1 As3 87.1(2) . . ?

As2 As1 As3 87.1(2) 1_565 . ?

As3' As1' As2' 87.8(3) . 1_545 ?

As3' As1' As2' 87.8(3) . . ?

As2' As1' As2' 93.6(3) 1_545 . ?

As2' As2 As1 94.1(2) . 1_545 ?

As2' As2 As1 94.1(2) . . ?

As1 As2 As1 93.3(3) 1_545 . ?

As2 As2' As1' 94.4(2) . 1_565 ?

As2 As2' As1' 94.4(2) . . ?

As1' As2' As1' 93.6(3) 1_565 . ?

As4 As3 As4 93.1(3) 1_675 1_665 ?

As4 As3 As1 99.8(2) 1_675 . ?

As4 As3 As1 99.8(2) 1_665 . ?

As4' As3' As4' 93.9(3) 1_565 . ?

As4' As3' As1' 100.7(3) 1_565 . ?

As4' As3' As1' 100.7(3) . . ?

As4' As4 As3 98.1(2) . 1_435 ?

As4' As4 As3 98.1(2) . 1_445 ?

As3 As4 As3 93.1(3) 1_435 1_445 ?

As4 As4' As3' 97.5(2) . 1_545 ?

As4 As4' As3' 97.5(2) . . ?

As3' As4' As3' 93.9(3) 1_545 . ?

_refine_diff_density_max 3.360

_refine_diff_density_min -1.303

_refine_diff_density_rms 0.585

_shelx_res_file

;

TITL exp_2 in P21nm #31

shelx.res

created by SHELXL-2016/6 at 21:11:45 on 29-Mar-2018

REM transformed to space group :

REM reset to P21nm #31

CELL 0.71073 10.1193 3.6288 10.3152 90.000 90.000 90.000

ZERR 8 0.0007 0.0002 0.0010 0.000 0.000 0.000

LATT -1

SYMM -X,0.5-Y,0.5+Z

SYMM -X,0.5+Y,0.5+Z

SYMM +X,-Y,+Z

SFAC As

UNIT 16

L.S. 10

PLAN 5

BOND

fmap 2

acta

MERG 2

OMIT -3 55

REM <olex2.extras>

REM <HklSrc "%.\\exp_2.hkl">

REM </olex2.extras>

WGHT 0.089000 0.583000

FVAR 0.08245

AS1 1 0.470695 1.500000 -0.765738 10.50000 0.01056 0.00921 =

0.02013 0.00000 -0.00684 0.00000

AS1' 1 0.035294 0.500000 -0.724257 10.50000 0.00915 0.01483 =

0.04274 0.00000 0.00525 0.00000

AS2 1 0.340019 1.000000 -0.660371 10.50000 0.01925 0.00481 =

0.03773 0.00000 0.00176 0.00000

AS2' 1 0.165949 1.000000 -0.828390 10.50000 0.00915 0.01947 =

0.02418 0.00000 -0.00108 0.00000

AS3 1 0.613933 1.500000 -0.566900 10.50000 0.02463 0.00833 =

0.01106 0.00000 0.00041 0.00000

AS3' 1 -0.108769 0.500000 -0.919782 10.50000 0.00901 0.01339 =

0.03838 0.00000 -0.00045 0.00000

AS4 1 -0.227510 0.000000 -0.626361 10.50000 0.01476 0.00232 =

0.03080 0.00000 -0.00040 0.00000

AS4' 1 -0.266125 0.000000 -0.863179 10.50000 0.01002 0.02444 =

0.02919 0.00000 -0.00302 0.00000

HKLF 4

REM exp_2 in P21nm #31

REM R1 = 0.0545 for 743 Fo > 4sig(Fo) and 0.0718 for all 935 data

REM 49 parameters refined using 1 restraints

END

WGHT 0.0887 0.5529

REM Highest difference peak 3.360, deepest hole -1.303, 1-sigma level 0.585

Q1 1 -0.2957 -0.5000 -0.7595 10.50000 0.05 3.36

Q2 1 0.3528 1.5000 -0.7613 10.50000 0.05 3.11

Q3 1 0.5704 1.5000 -0.3753 10.50000 0.05 3.06

Q4 1 -0.2148 -0.5000 -0.7304 10.50000 0.05 2.87

Q5 1 -0.0738 0.5000 -1.1124 10.50000 0.05 2.83

;

_shelx_res_checksum 86750

_shelx_hkl_file

;

0 0 -1 0.00500 0.19671 8

0 0 1-0.01174 0.15001 8

0 0 1-0.04990 0.16197 2

0 0 -1 0.11579 0.13182 2

0 0 2 97.5985 2.31644 2

0 0 2 92.9546 2.31479 8

0 0 -2 95.7543 2.33293 2

0 0 -2 92.8310 2.34404 8

0 0 3-0.33869 0.43259 2

0 0 3 0.19847 0.47264 8

0 0 -3 0.18540 0.46676 2

0 0 -3-0.71192 0.57980 8

0 0 4 58.0194 1.66995 8

0 0 -4 58.1689 1.74872 2

0 0 4 59.0436 1.66292 2

0 0 -4 57.4989 1.78433 8

0 0 5 0.51668 0.55259 8

0 0 -5 0.15408 0.66831 2

0 0 -5-0.61338 0.75506 8

0 0 5-0.12059 0.50778 2

0 0 -6 7.44712 0.95983 8

0 0 6 8.24300 0.73035 2

0 0 -6 9.26720 0.91694 2

0 0 6 8.44714 0.75749 8

0 0 -7-0.21123 0.96866 8

0 0 -7-0.45529 0.86645 2

0 0 -8 0.62099 0.93875 2

0 0 -9 1.30600 1.07785 2

0 0 9 2.85637 1.21587 4

0 0 10 24.7229 1.75803 4

0 0 11-0.73784 1.38235 4

0 0 12 9.44479 1.62506 4

0 0 13 1.56615 1.56140 4

0 0 14 8.90003 1.70948 4

1 0 0 0.07677 0.12321 7

-1 0 0-0.00785 0.09334 7

-1 0 -1 0.60507 0.19065 2

-1 0 1 0.55549 0.20433 7

1 0 -1 0.54722 0.16340 4

1 0 1 0.56927 0.16998 2

-1 0 1 0.82603 0.18704 4

-1 0 -2 52.2392 1.28210 2

1 0 2 49.9508 1.26074 2

-1 0 2 46.1870 1.24742 8

-1 0 2 48.2181 1.26710 4

1 0 -2 46.2651 1.24902 8

-1 0 2 45.9083 1.14661 6

-1 0 3 147.751 3.71118 8

-1 0 3 160.592 3.80507 4

1 0 3 149.346 3.73672 8

1 0 3 158.710 3.74553 2

1 0 -3 145.635 3.72270 8

-1 0 -3 155.709 3.75870 2

-1 0 3 167.537 3.73831 2

1 0 4 38.7719 1.32091 8

-1 0 4 42.5878 1.42948 4

1 0 -4 38.0086 1.31688 8

1 0 4 39.6301 1.28370 2

-1 0 -4 43.1989 1.35285 2

-1 0 4 45.3171 1.28688 2

-1 0 4 39.1482 1.28361 8

1 0 5 2.04153 0.54532 2

-1 0 5 2.24794 0.54590 2

-1 0 -5 2.37525 0.64322 2

-1 0 5 2.57204 0.77084 4

-1 0 5 3.86736 0.64681 8

1 0 5 1.83892 0.65069 8

1 0 -5 3.33752 0.75596 8

-1 0 6 3.38214 0.87633 4

-1 0 -6 2.28965 0.74614 2

-1 0 6 3.09375 0.67653 8

1 0 6 3.89592 0.70859 8

-1 0 6 2.90227 0.60840 2

1 0 -6 3.06686 0.88132 8

1 0 6 2.66622 0.64406 2

-1 0 -7 35.4754 1.58199 2

-1 0 7 41.2804 1.73990 4

1 0 -7 36.0337 1.64429 8

-1 0 8 17.7266 1.40088 4

1 0 -8 13.1985 1.33113 8

-1 0 -8 17.0071 1.29840 2

-1 0 -9 1.55063 1.09040 2

1 0 -9 1.59260 1.18047 8

-1 0 9 1.92789 1.23604 4

-1 0 -10 64.1338 2.52604 2

1 0 -10 63.2402 2.55356 8

-1 0 10 73.6388 2.67593 4

-1 0 -11 3.68968 1.31962 2

1 0 11 2.60632 1.41477 4

-1 0 11 3.28299 1.38516 4

1 0 12 0.56877 1.45772 4

-1 0 12 3.35966 1.47429 4

-1 0 13 14.1505 1.76152 4

1 0 13 14.7814 1.84004 4

1 0 14-1.79048 1.60052 4

-1 0 14 0.30710 1.55929 4

-2 0 0 9.93245 0.38453 7

2 0 0 8.60535 0.38038 4

2 0 0 11.2880 0.40797 7

-2 0 1 11.9197 0.46797 7

-2 0 1 11.5820 0.40156 6

2 0 -1 11.8343 0.49217 7

-2 0 1 11.6847 0.44299 4

-2 0 -1 11.1029 0.44888 2

2 0 1 11.8018 0.45216 7

2 0 -1 11.2725 0.41104 6

2 0 -1 11.9251 0.43531 4

2 0 1 10.2048 0.43786 2

2 0 -2 16.3396 0.53670 6

-2 0 2 17.7703 0.52460 6

-2 0 -2 18.0427 0.65235 2

-2 0 2 18.2970 0.62070 4

2 0 -2 17.2367 0.61277 4

2 0 2 19.0366 0.62850 2

-2 0 2 18.1645 0.67123 7

-2 0 3 105.120 2.45899 4

-2 0 3 94.3546 2.29419 6

2 0 -3 97.4864 2.45873 4

2 0 -3 102.161 2.49001 8

-2 0 -3 95.9285 2.47400 2

2 0 3 93.6225 2.42786 2

-2 0 4 206.086 4.85078 4

-2 0 4 192.382 4.61989 6

2 0 4 205.615 4.81665 2

-2 0 4 202.383 4.78259 2

-2 0 -4 198.787 4.86207 2

-2 0 4 203.355 4.80436 8

2 0 -4 197.091 4.82056 8

-2 0 5 27.2231 1.06567 8

-2 0 5 25.2129 0.78898 6

-2 0 5 25.3149 1.10856 4

2 0 5 22.6886 1.03400 8

-2 0 5 28.1252 1.01302 2

-2 0 -5 24.0364 1.07253 2

2 0 5 24.9619 1.01523 2

2 0 -5 26.2758 1.07394 8

-2 0 6 69.9391 2.19116 4

-2 0 6 70.3440 2.04909 8

2 0 6 62.9132 1.99966 2

-2 0 -6 71.7176 2.14534 2

2 0 6 65.6479 2.05041 8

-2 0 6 68.6868 1.98286 2

-2 0 6 67.5536 1.77161 6

2 0 -6 70.4065 2.13758 8

-2 0 7 111.697 3.18952 4

-2 0 -7 108.638 3.13154 2

2 0 -7 105.653 3.13562 8

-2 0 8 3.46314 1.04502 4

2 0 -8 3.52843 1.12433 8

-2 0 -8 3.97254 0.97591 2

-2 0 9 6.00271 1.23241 4

2 0 -9 4.67365 1.27874 8

-2 0 -9 5.76915 1.14484 2

-2 0 -10 1.07743 1.25696 2

-2 0 10 2.90317 1.27543 4

2 0 -10 1.63492 1.34705 8

-2 0 11 1.44751 1.34020 4

-2 0 -11 2.87731 1.32190 2

2 0 -11 1.01796 1.46440 8

-2 0 -12 3.11702 1.42247 2

-2 0 12 1.51082 1.45224 4

2 0 12 3.44114 1.52141 4

-2 0 -13 4.32156 1.53968 2

-2 0 13 5.53742 1.63899 4

2 0 13 4.57498 1.62889 4

-2 0 14 1.70002 1.56772 4

2 0 14-0.12877 1.62362 4

3 0 0 0.26249 0.34070 7

-3 0 0 0.30761 0.31112 7

3 0 0-0.02083 0.32103 4

3 0 -1 86.4332 2.08518 4

-3 0 1 84.7015 2.11548 7

-3 0 -1 79.0535 2.08917 7

3 0 1 83.9852 2.10150 7

-3 0 -1 85.1117 2.12737 2

-3 0 1 85.5681 2.09103 4

3 0 -1 84.7068 2.08549 6

3 0 -1 87.8947 2.15504 7

3 0 -2 85.3046 2.13692 4

-3 0 2 83.0246 2.08066 6

-3 0 2 83.8960 2.15362 4

3 0 2 95.9359 2.19734 7

-3 0 -2 90.7595 2.20634 2

3 0 -2 83.5450 2.24934 7

-3 0 2 80.3465 2.18563 7

3 0 2 97.8512 2.17861 2

3 0 -2 78.5913 2.09189 6

-3 0 -3 44.2775 1.39744 2

-3 0 3 49.6468 1.36283 4

-3 0 3 47.6858 1.22373 6

-3 0 3 47.5524 1.40198 7

3 0 -3 47.2466 1.23926 6

3 0 -3 47.5637 1.34000 4

3 0 3 45.9287 1.13464 5

3 0 -3 50.5420 1.44087 8

3 0 3 48.8187 1.34308 2

3 0 -4 25.3939 1.02538 8

-3 0 4 24.2484 0.99956 7

-3 0 -4 25.3560 0.99751 2

-3 0 4 25.5430 0.96314 4

-3 0 4 21.5001 0.72089 6

3 0 4 23.2682 0.88264 2

3 0 -4 23.6929 0.94227 4

3 0 -5 48.3859 1.58076 8

-3 0 5 50.6224 1.53132 4

3 0 5 48.5832 1.47288 2

-3 0 5 45.4659 1.57805 7

-3 0 -5 50.8061 1.59715 2

-3 0 5 44.4655 1.23968 6

-3 0 6 37.3621 1.39626 4

3 0 6 32.6129 1.30602 8

-3 0 -6 34.0054 1.42447 2

3 0 -6 34.2927 1.40738 8

3 0 -7 13.9546 1.11174 8

-3 0 -7 11.5118 1.03141 2

-3 0 7 13.5772 1.09638 4

3 0 -8 4.85797 1.12762 8

-3 0 8 3.89836 1.04803 4

-3 0 -8 0.70268 0.90026 2

3 0 -9 53.0242 2.23344 8

-3 0 9 55.2013 2.17856 4

-3 0 -9 49.9998 2.09481 2

-3 0 10 8.56964 1.36015 4

3 0 -10 7.36334 1.49186 8

-3 0 -10 7.96717 1.32013 2

-3 0 11 12.0949 1.51504 4

3 0 -11 7.45934 1.66973 8

-3 0 -11 8.95808 1.48078 2

-3 0 12 7.19434 1.51651 4

3 0 -12 7.16142 1.74851 8

-3 0 -12 6.56691 1.55595 2

-3 0 -13 3.77522 1.56631 2

-3 0 13 5.54666 1.58893 4

3 0 13 0.87682 1.57609 4

3 0 -13 1.45982 1.78492 8

3 0 14 9.45441 1.79232 4

-3 0 14 13.9152 1.81066 4

-3 0 -14 12.1693 1.74306 2

-4 0 0 0.71026 0.41530 7

4 0 0 0.81007 0.43857 6

4 0 0 0.76784 0.39256 4

4 0 0 0.96283 0.41423 7

4 0 -1 94.4567 2.37067 6

4 0 1 94.7938 2.38239 7

-4 0 1 97.6172 2.39905 7

-4 0 -1 90.6381 2.36789 7

-4 0 1 93.6898 2.37035 4

4 0 -1 92.0086 2.34269 4

-4 0 -1 93.6692 2.42458 2

4 0 -1 99.0800 2.44801 7

4 0 -2 61.1690 1.78726 7

-4 0 -2 62.4727 1.73237 2

4 0 -2 63.6976 1.65153 4

-4 0 2 62.0033 1.66988 4

4 0 2 61.3392 1.68157 7

4 0 -2 60.3823 1.62936 6

-4 0 2 60.2927 1.68706 7

4 0 -3 105.059 2.64097 6

-4 0 3 108.212 2.73543 4

4 0 -3 108.195 2.71912 4

4 0 3 110.045 2.71822 2

4 0 3 110.229 2.75685 7

-4 0 -3 113.850 2.81808 2

-4 0 3 104.255 2.77537 7

-4 0 3 106.608 2.62367 6

-4 0 4 3.18050 0.39164 6

4 0 -4 2.74116 0.53420 4

-4 0 4 3.20740 0.56956 7

4 0 -4 3.12100 0.40539 6

-4 0 4 3.17853 0.55835 4

-4 0 -4 4.16624 0.65427 2

4 0 4 4.56387 0.53679 2

4 0 -4 3.88088 0.71989 8

4 0 -5 5.45657 0.81150 8

4 0 -5 4.61068 0.64930 4

-4 0 5 5.63930 0.70478 4

-4 0 -5 4.31492 0.73208 2

4 0 -6 0.62675 0.82865 8

-4 0 -6 0.07672 0.78185 2

-4 0 6 0.59946 0.72644 4

-4 0 7 1.74588 0.82173 4

4 0 -7 0.60088 0.89579 8

-4 0 -7 0.60534 0.86299 2

-4 0 -8 83.6556 2.75009 2

-4 0 8 87.1405 2.70835 4

4 0 -8 85.4244 2.76850 8

4 0 -9 1.35024 1.20653 8

-4 0 -9 0.57283 1.08046 2

-4 0 9 1.45472 1.06089 4

-4 0 -10-0.44174 1.19536 2

-4 0 10 0.86361 1.18014 4

4 0 -10 0.54166 1.42331 8

-4 0 11 3.36886 1.36880 4

4 0 -11 1.91757 1.58465 8

-4 0 -11 2.66154 1.35016 2

-4 0 -12 22.7644 1.90935 2

-4 0 12 24.9447 1.90028 4

4 0 -12 20.5908 2.12368 8

-4 0 -13 6.84283 1.72239 2

4 0 -13 4.03784 1.91121 8

-4 0 13 10.1035 1.70136 4

-4 0 14 3.45470 1.61274 4

5 0 0-0.21806 0.41674 4

5 0 0 0.45101 0.44540 7

-5 0 0 0.18013 0.43003 7

5 0 0-0.13055 0.49413 6

5 0 -1 10.3522 0.67815 7

-5 0 -1 8.94361 0.60370 7

-5 0 -1 9.64122 0.68372 2

-5 0 1 8.80925 0.58780 7

5 0 -1 8.35519 0.56064 4

5 0 1 10.7529 0.66060 7

5 0 -1 8.49855 0.61494 6

5 0 -2 66.6025 1.98706 7

-5 0 -2 69.3669 1.95017 2

5 0 -2 68.7767 1.82354 4

5 0 -2 68.2785 1.84585 6

-5 0 2 66.8252 1.85470 4

5 0 2 68.9286 1.87731 7

-5 0 2 66.0566 1.86983 7

5 0 3 39.8334 1.25582 2

-5 0 3 36.9498 1.26944 7

-5 0 3 41.1637 1.28381 4

5 0 -3 38.3313 1.21392 4

5 0 -3 36.5848 1.17165 6

-5 0 -3 40.7311 1.34622 2

5 0 3 38.8693 1.29084 7

5 0 4 32.0885 1.17899 7

-5 0 4 31.5028 1.00743 6

5 0 -4 35.5888 1.35224 8

5 0 -4 29.8925 1.02060 6

-5 0 4 31.8947 1.15648 4

5 0 -4 30.6539 1.11778 4

-5 0 -4 33.6163 1.29120 2

5 0 -5 169.180 4.48881 4

-5 0 -5 178.976 4.62678 2

5 0 -5 192.838 4.66016 8

-5 0 5 182.999 4.50331 4

5 0 -6 0.66967 0.96435 8

-5 0 -6 1.53042 0.82693 2

-5 0 6 2.04398 0.74465 4

-5 0 7 16.5781 1.15149 4

5 0 -7 18.0419 1.32536 8

-5 0 -7 16.9173 1.22869 2

5 0 -8 19.8944 1.44467 8

-5 0 8 19.4527 1.30781 4

-5 0 -8 19.5469 1.39550 2

-5 0 9 5.99233 1.18121 4

5 0 -9 8.29055 1.36936 8

-5 0 -9 5.19671 1.22935 2

-5 0 10 0.72338 1.26496 4

-5 0 -10 0.25315 1.23131 2

5 0 -10 4.18225 1.49230 8

-5 0 -11 0.36005 1.34402 2

-5 0 11 1.40795 1.43793 4

5 0 -11 0.84232 1.65466 8

5 0 -12 7.33298 1.94834 8

-5 0 -12 10.1378 1.62843 2

-5 0 12 12.8508 1.68016 4

-5 0 -13 1.30354 1.60156 2

5 0 -13-0.97494 1.96422 8

6 0 0 0.78987 0.54821 7

-6 0 0 0.95976 0.48441 7

6 0 0 0.92839 0.60016 6

6 0 0-0.22022 0.46370 4

6 0 1 50.6390 1.62959 6

6 0 -1 51.5421 1.58169 7

-6 0 -1 45.8129 1.47572 7

-6 0 -1 48.7465 1.57946 2

6 0 -1 46.7235 1.44452 4

6 0 1 50.2656 1.51491 7

6 0 -1 50.0933 1.52076 6

-6 0 1 49.5066 1.49398 7

6 0 -2 54.7180 1.52206 4

-6 0 2 46.5520 1.27626 3

6 0 -2 53.6827 1.72167 7

6 0 -2 55.1109 1.57193 6

6 0 2 52.0528 1.56505 7

-6 0 2 57.0697 1.58612 4

-6 0 2 53.5017 1.58786 7

-6 0 -2 54.6789 1.67259 2

6 0 3 0.63309 0.57114 7

6 0 -3 0.75816 0.71118 7

-6 0 3 0.69415 0.55441 4

6 0 -3 1.03383 0.55458 6

6 0 -3 1.03236 0.49947 4

-6 0 -3 1.13343 0.64585 2

-6 0 4 40.5483 1.32642 4

6 0 -4 38.0531 1.22410 4

6 0 -5 73.6187 2.24577 8

6 0 5 61.4149 2.01188 7

-6 0 5 66.4927 2.00549 4

-6 0 -5 62.2899 2.12974 2

-6 0 6 26.6091 1.23543 4

6 0 6 23.3713 1.22462 7

6 0 -6 28.6776 1.47362 8

-6 0 -6 25.1885 1.36458 2

6 0 -7 2.83081 1.12154 8

-6 0 7 2.08654 0.87908 4

-6 0 -7 2.49152 1.02463 2

-6 0 8 7.14932 1.08520 4

6 0 -8 5.42497 1.22740 8

-6 0 -8 4.62538 1.18340 2

6 0 -9 9.12750 1.47395 8

-6 0 9 9.44374 1.26633 4

-6 0 -9 8.10546 1.34954 2

-6 0 -10-0.18562 1.28297 2

6 0 -10 3.94279 1.55152 8

-6 0 10 1.31928 1.26658 4

-6 0 -11 0.42870 1.42451 2

6 0 -11 3.59693 1.71551 8

6 0 -12 6.69279 1.96569 8

-6 0 -12 6.31083 1.61376 2

-6 0 -13 0.16419 1.61656 2

6 0 -13-0.42093 2.04249 8

7 0 0 3.19018 0.58962 4

-7 0 0 4.36312 0.65154 7

7 0 0 5.66297 0.72929 7

7 0 0 5.70972 0.79672 6

7 0 1 261.081 6.42169 6

7 0 -1 262.460 6.39497 7

-7 0 -1 257.495 6.28969 7

-7 0 -1 270.100 6.43920 2

7 0 -1 265.866 6.34620 6

7 0 1 257.934 6.24269 4

7 0 -1 261.514 6.25040 4

7 0 1 262.883 6.33172 7

7 0 2 77.8735 2.39789 6

-7 0 -2 77.4477 2.29647 2

7 0 -2 76.5252 2.33085 7

7 0 -2 73.7768 2.18899 6

7 0 -2 75.9157 2.12115 4

7 0 2 82.4898 2.25076 7

7 0 3 5.90502 0.72713 7

7 0 -3 5.89998 0.91893 7

7 0 -3 5.82666 0.68811 6

-7 0 -3 5.30145 0.83220 2

-7 0 3 6.19011 0.73156 4

-7 0 4 4.91290 0.73814 4

-7 0 -4 4.44822 0.86328 2

7 0 4 4.43085 0.74266 7

7 0 -5 2.97979 1.05564 8

-7 0 5 2.07614 0.74949 4

7 0 5 1.44777 0.73930 7

-7 0 -5 1.56535 0.92883 2

-7 0 6 12.0990 1.00288 4

7 0 -6 12.6587 1.27938 8

-7 0 -6 9.87812 1.15879 2

-7 0 -7 1.69943 1.08977 2

7 0 -7 2.70444 1.20136 8

-7 0 7 2.74329 0.94340 4

-7 0 -8 8.10069 1.34189 2

7 0 -8 9.27950 1.41937 8

-7 0 -9 0.63055 1.30035 2

7 0 -9-0.76936 1.42944 8

7 0 -10 4.67495 1.57154 8

-7 0 -10-0.87655 1.40275 2

7 0 -11 12.6862 1.95406 8

-7 0 -11 9.92257 1.64520 2

-7 0 -12-0.51136 1.57919 2

7 0 -12 1.93049 1.97366 8

8 0 0 29.9277 1.26317 7

8 0 0 27.6546 1.28665 6

8 0 -1 4.16295 0.81948 6

8 0 1 4.20126 0.88238 6

8 0 -1 3.07090 0.82055 7

-8 0 -1 4.08706 0.87862 2

8 0 1 5.15101 0.77794 7

8 0 -2 10.6869 0.88565 6

8 0 2 11.4653 1.12040 6

8 0 -2 11.1234 1.04318 7

-8 0 -2 11.4611 1.00744 2

8 0 2 12.6505 0.90234 7

8 0 -3 1.55620 0.92830 7

8 0 3 2.23866 0.76542 7

-8 0 -3 1.66483 0.86090 2

8 0 3 2.81431 1.08729 6

8 0 4 9.17288 0.89100 7

-8 0 -4 9.43499 1.05905 2

8 0 -5 1.54321 1.15140 8

-8 0 -5-0.59130 0.98823 2

8 0 -6 12.4982 1.40004 8

-8 0 -6 8.55148 1.25353 2

-8 0 -7-0.11681 1.15131 2

8 0 -7 1.50415 1.33068 8

8 0 -8 0.69043 1.39255 8

-8 0 -8-0.10256 1.24972 2

8 0 -9 55.0627 2.46873 8

-8 0 -9 48.8353 2.32891 2

8 0 -10 4.20440 1.63406 8

-8 0 -10 0.79651 1.45364 2

8 0 -11 30.4581 2.30821 8

-8 0 -11 24.3675 1.97364 2

-8 0 -12 0.03289 1.61849 2

8 0 -12 2.42187 1.96149 8

-9 0 0-0.19618 0.91315 2

9 0 0 1.43832 0.86279 6

9 0 0 1.18204 0.83265 7

-9 0 -1 17.3977 1.19695 2

9 0 1 18.0056 1.09846 7

9 0 1 16.7379 1.21277 6

9 0 -1 17.4675 1.14478 6

9 0 -1 17.3619 1.18836 7

9 0 2 18.5771 1.34249 6

-9 0 -2 17.4038 1.21024 2

9 0 2 17.0451 1.09609 7

9 0 -2 22.0872 1.30907 7

9 0 3 34.8752 1.46075 7

9 0 3 36.2469 1.80102 6

-9 0 -3 35.2899 1.61003 2

9 0 -3 34.3025 1.66287 7

9 0 4 53.0001 2.34362 6

-9 0 -4 56.2461 2.09991 2

9 0 -5 17.5445 1.48238 8

9 0 5 14.1283 1.66140 6

-9 0 -5 15.0142 1.36891 2

9 0 6 17.7274 1.86260 6

9 0 -6 25.4390 1.72403 8

-9 0 -6 23.3327 1.62046 2

-9 0 -7 2.67705 1.26900 2

9 0 -7 3.72416 1.42761 8

-9 0 -8 26.2130 1.86660 2

9 0 -8 29.9635 2.02987 8

9 0 -9 1.09764 1.58792 8

-9 0 -9 0.99548 1.45659 2

9 0 9 1.02986 1.81140 6

-9 0 -10 3.33064 1.59427 2

9 0 -10 5.89578 1.79488 8

9 0 10 5.33558 1.86400 6

-9 0 -11 0.89082 1.60164 2

9 0 -11 3.99237 1.92144 8

9 0 11 6.26491 1.84253 6

10 0 0 12.3775 1.10875 7

10 0 0 13.2931 1.19125 6

-10 0 0 13.2590 1.25153 2

10 0 -1 5.14936 1.04597 7

-10 0 -1 4.37548 1.06799 2

10 0 1 5.18247 0.94036 7

10 0 1 3.35158 1.07728 6

10 0 -2 27.8736 1.55583 7

10 0 2 27.1755 1.63230 6

-10 0 -2 30.1162 1.59637 2

10 0 3 1.43078 1.25083 6

-10 0 -3 0.87522 1.06671 2

-10 0 -4 0.45259 1.13286 2

10 0 4 1.97661 1.38883 6

10 0 5 12.2222 1.72122 6

-10 0 -5 9.70740 1.32113 2

10 0 -6 13.7514 1.65094 8

10 0 6 11.0716 1.83171 6

-10 0 -6 10.8702 1.49115 2

-10 0 -7 26.4151 1.84773 2

10 0 7 19.3172 2.15595 6

10 0 -7 30.4860 1.96207 8

10 0 -8 8.81136 1.74868 8

-10 0 -8 4.31114 1.50005 2

10 0 8 3.24693 1.94395 6

-10 0 -9 1.97841 1.55902 2

10 0 9 6.24708 1.97893 6

10 0 -9 3.49074 1.75315 8

10 0 -10 3.51586 1.84278 8

10 0 10 5.60639 1.96994 6

-10 0 -10 0.78588 1.59962 2

11 0 0 0.79381 1.12014 6

-11 0 0 0.33036 1.11152 2

11 0 0 0.87555 1.03082 7

11 0 1 0.80649 1.15088 6

11 0 -1 1.91284 1.12223 7

-11 0 -1 0.62233 1.10193 2

-11 0 -2 34.6084 1.79275 2

11 0 -2 31.8180 1.73305 7

11 0 2 30.3823 1.79544 6

11 0 3 27.2306 1.86507 6

-11 0 -3 31.2873 1.73022 2

11 0 4 4.27530 1.51611 6

-11 0 -4 2.80553 1.24794 2

11 0 -5 4.13510 1.49938 8

11 0 5 4.16181 1.68085 6

-11 0 -5 1.54974 1.30274 2

11 0 6 3.14143 1.81648 6

11 0 -6 3.28794 1.59103 8

-11 0 -6 1.18317 1.39215 2

11 0 -7 11.0757 1.77052 8

11 0 7 6.94074 2.02123 6

-11 0 -7 8.61906 1.56564 2

-11 0 -8 1.64847 1.53530 2

11 0 8-0.11970 1.91788 6

11 0 -8 1.89515 1.72193 8

11 0 -9 6.56784 1.87748 8

-11 0 -9 7.28777 1.71633 2

11 0 9 8.70008 2.14364 6

-12 0 0 3.61490 1.33993 2

-12 0 1 28.0075 1.78338 2

-12 0 -1 30.2957 1.78531 2

12 0 1 27.8887 1.77310 6

12 0 2 4.35656 1.47746 6

-12 0 -2 1.71105 1.34528 2

12 0 -2 1.21583 1.24426 7

12 0 3 12.3606 1.67627 6

-12 0 -3 14.1906 1.50178 2

-12 0 -4 11.9581 1.56385 2

12 0 4 14.3872 1.85377 6

-12 0 -5 1.36865 1.40300 2

12 0 5 2.25005 1.79059 6

12 0 -5 3.21786 1.58415 8

12 0 -6 29.3910 2.08275 8

-12 0 -6 26.0547 1.95123 2

12 0 6 29.1216 2.39814 6

12 0 -7 2.40974 1.67286 8

-12 0 -7-0.78453 1.52531 2

12 0 7 2.04522 1.98875 6

-13 0 0 0.27184 1.28554 2

-13 0 1 8.31412 1.48586 2

-13 0 -1 7.97832 1.46798 2

13 0 2 7.41705 1.61295 6

-13 0 -2 7.98821 1.45451 2

-13 0 2 4.82332 1.43329 2

-13 0 -3 5.03668 1.43266 2

13 0 3 4.66678 1.68065 6

-13 0 -4 23.3548 1.82754 2

13 0 4 21.1645 2.07897 6

13 0 -5 3.90037 1.67616 8

13 0 5 2.07965 1.81434 6

-13 0 -5 1.80936 1.51040 2

13 0 6 7.25365 2.08946 6

-13 0 -6 5.93306 1.65627 2

13 0 -6 8.94440 1.82442 8

-14 0 0 27.5284 1.92912 2

-14 0 1 15.2193 1.69087 2

-14 0 -1 17.8210 1.78771 2

-14 0 2 1.34251 1.47977 2

-14 0 -2 3.62497 1.50564 2

14 0 3 15.5456 1.95921 6

0 1 0 0.30574 0.43115 1

0 -1 0-0.03105 0.23254 4

0 -1 0 0.03986 0.29807 3

0 -1 0 0.27190 0.40052 1

0 -1 0 0.03630 0.28017 6

0 1 1 2.09635 0.34156 6

0 1 1 2.33588 0.41442 3

0 1 -1 1.89318 0.44294 1

0 1 1 1.92299 0.35008 4

0 -1 -1 2.05951 0.29525 6

0 -1 -1 2.31954 0.37531 5

0 -1 -1 2.09436 0.37009 3

0 -1 1 2.23196 0.43475 8

0 1 1 2.22702 0.26328 7

0 -1 -1 1.58102 0.26238 7

0 1 1 2.77741 0.23485 2

0 1 1 1.94323 0.45040 1

0 -1 1 1.82243 0.43663 1

0 -1 -1 2.32832 0.31083 4

0 -1 -1 1.79160 0.38416 1

0 -1 1 2.18944 0.23103 2

0 1 2 0.02469 0.35950 5

0 1 2-0.02673 0.40148 1

0 -1 -2 0.27414 0.27640 6

0 1 2-0.00537 0.36304 4

0 -1 -2 0.04632 0.28499 7

0 1 -2 0.01456 0.35559 2

0 -1 -2 0.15784 0.20940 8

0 1 2-0.08882 0.30119 6

0 -1 -2-0.03177 0.40028 3

0 -1 2-0.22145 0.32439 2

0 -1 -2 0.30056 0.40889 1

0 -1 2-0.32473 0.49904 8

0 1 2-0.16368 0.44479 3

0 1 2 0.08962 0.33350 2

0 -1 -2 0.32249 0.35877 5

0 -1 2 0.19076 0.38425 1

0 1 -2-0.12565 0.43735 1

0 -1 -2 0.00965 0.35297 2

0 1 2-0.00317 0.23853 7

0 -1 -2 0.01475 0.33410 4

0 1 3 20.3860 0.79802 2

0 1 3 20.4804 0.67449 6

0 1 -3 21.0523 0.86140 2

0 1 3 23.3834 0.87996 3

0 1 3 21.2403 0.79158 1

0 -1 -3 22.4127 0.82352 1

0 -1 -3 19.6294 0.83733 2

0 1 3 24.1685 0.84601 5

0 -1 3 19.9983 0.79472 2

0 1 3 21.7123 0.67738 7

0 -1 -3 20.6458 0.81236 4

0 -1 3 23.6063 0.96820 8

0 -1 3 23.9799 0.82404 1

0 -1 -3 21.6840 0.68045 8

0 1 3 22.2948 0.81830 4

0 1 -3 21.3251 0.79470 1

0 -1 -3 20.1384 0.67287 6

0 1 4-0.11152 0.48430 2

0 -1 -4 0.05971 0.40058 1

0 -1 4-0.05742 0.37063 1

0 -1 4-0.15164 0.46407 2

0 1 4 0.77220 0.54509 4

0 1 -4 0.28893 0.56983 2

0 -1 -4 0.18371 0.57709 2

0 1 -4 0.17120 0.41498 1

0 1 4 0.25483 0.38468 1

0 1 4 0.33782 0.56410 3

0 1 4-0.02763 0.34710 7

0 -1 -4 0.29295 0.45997 8

0 -1 4 0.35479 0.62197 8

0 1 4 0.02598 0.47143 5

0 1 4-0.16592 0.31975 6

0 -1 -5 478.919 11.0956 8

0 1 5 462.736 11.0027 8

0 -1 -5 474.148 10.9803 1

0 1 -5 499.091 11.2683 2

0 -1 5 472.520 10.9640 1

0 1 5 489.852 11.1919 3

0 1 5 467.025 11.1253 5

0 1 5 480.677 11.2373 4

0 -1 -5 456.181 11.2271 2

0 1 5 466.501 11.1334 2

0 1 -5 486.034 11.0088 1

0 -1 5 496.656 11.2099 8

0 -1 5 487.045 11.1307 2

0 1 5 482.055 11.0263 7

0 1 5 476.465 10.9361 6

0 1 6 0.32871 0.45140 6

0 -1 -6-0.27036 0.74260 8

0 -1 6 0.57737 0.60659 2

0 1 -6 0.31978 0.76770 2

0 1 6-0.23176 0.54407 7

0 1 6 0.55355 0.60943 2

0 1 6 0.42907 0.77082 4

0 1 6-0.07077 0.55652 8

0 1 6 0.16520 0.80321 3

0 -1 -6-0.31672 0.75495 2

0 -1 -7 31.3267 1.48073 2

0 1 7 27.1546 1.50657 4

0 1 7 35.9105 1.68268 3

0 1 -7 33.4035 1.51116 2

0 -1 -7 32.8922 1.42483 8

0 1 8-0.61691 1.07031 4

0 1 8-1.45219 1.34150 3

0 -1 -8-0.52491 1.03989 2

0 1 -8-0.64706 1.01671 2

0 1 9 20.1710 2.00735 3

0 1 9 34.1999 1.80900 4

0 1 10 0.77411 1.28433 4

0 1 10-1.57566 1.61959 3

0 1 11 4.06571 1.46130 4

0 1 11 4.97738 1.67651 3

0 1 12 1.12169 1.48255 4

0 1 12 3.55014 1.67035 3

0 1 13 1.24907 1.60368 4

0 1 13 4.57816 1.69792 3

0 -1 13 0.26714 1.52715 4

0 1 14 1.07826 1.63008 4

0 -1 14-0.33692 1.56013 4

-1 -1 14 1.13825 1.50233 4

1 1 14 5.91107 1.74985 3

1 1 14 3.29670 1.69456 4

1 1 13 5.57126 1.73010 3

1 1 13 3.68194 1.60204 4

-1 -1 13-0.31663 1.47195 4

1 1 12 38.1226 2.31091 3

-1 -1 12 24.6575 1.90421 4

1 1 12 28.9501 2.03764 4

1 1 11 7.94360 1.79629 3

1 1 11 7.40939 1.51192 4

-1 -1 11 5.97305 1.41397 4

1 1 10 0.98870 1.34415 4

1 1 10-0.69300 1.67978 3

-1 1 -10 0.48118 1.17026 2

1 1 9 0.31686 1.68642 3

1 1 9 8.17779 1.33271 4

-1 1 -9 8.37980 1.29749 2

1 1 8 72.3998 2.84499 3

-1 1 -8 83.2189 2.64471 2

1 -1 -8 73.3449 2.47413 8

1 1 8 78.6597 2.72574 4

1 -1 -7 0.01401 0.82578 8

1 1 7 1.26934 0.68511 6

-1 1 -7 1.33962 0.92914 2

1 1 7 0.23433 1.03815 3

1 1 7-0.43041 1.02903 4

1 1 6 0.46876 0.61686 2

1 1 6 0.79618 0.56235 6

1 1 6 0.80689 0.57795 8

1 1 6 1.29953 0.83705 3

1 1 6-0.31444 0.89356 4

-1 1 -6 0.69208 0.73552 2

1 1 6 0.79879 0.61609 5

1 -1 -6 0.82191 0.66672 8

-1 -1 6 0.71054 0.56713 2

1 -1 -5 100.573 2.42219 8

-1 -1 5 99.6341 2.54684 8

1 1 5 94.5680 2.55272 3

-1 1 -5 97.9127 2.59381 2

-1 -1 5 85.4317 2.26396 1

1 1 5 90.8818 2.35631 6

1 1 5 86.5522 2.34643 8

1 1 5 86.8968 2.64615 4

-1 -1 5 89.3785 2.43424 2

-1 1 -5 88.4145 2.32549 1

1 1 5 94.6973 2.45797 5

1 1 5 76.7351 2.44172 2

1 -1 -4 1.83185 0.52505 4

-1 1 -4 1.85758 0.45234 1

1 -1 -4 1.50776 0.44134 8

1 1 4 1.67608 0.35000 7

1 1 4 1.42204 0.57919 3

1 1 4 1.97214 0.49398 2

1 1 4 1.58899 0.61004 4

-1 1 -4 1.24922 0.55066 2

-1 -1 4 1.39844 0.38188 1

1 1 4 1.97112 0.44987 6

1 1 4 1.83464 0.47900 5

-1 -1 4 1.10657 0.46164 2

1 1 4 1.55414 0.41205 8

1 -1 -4 1.92332 0.47364 1

-1 -1 4 1.74460 0.57693 8

1 -1 -3 53.0198 1.43189 8

1 1 3 55.3208 1.47693 6

1 1 3 51.1177 1.34990 7

-1 -1 3 55.9936 1.50857 1

-1 -1 3 54.4075 1.59325 8

1 -1 -3 52.6594 1.53037 7

-1 1 -3 61.2903 1.57776 2

1 1 3 55.5275 1.58251 3

-1 -1 3 59.8628 1.58652 6

1 -1 -3 57.0808 1.58068 1

1 1 3 58.2841 1.53801 5

-1 1 -3 61.8218 1.60785 1

1 -1 -3 48.8682 1.33654 6

1 1 3 58.3591 1.50881 2

-1 -1 3 61.3689 1.52469 2

1 1 3 53.7084 1.36889 8

1 1 3 56.9049 1.56331 4

1 -1 -3 51.2839 1.51774 4

-1 -1 2 3.73892 0.46913 6

1 -1 -2 4.70928 0.42933 4

-1 1 -2 4.21198 0.59467 1

1 1 2 3.85453 0.42252 6

1 1 2 3.68070 0.50198 3

-1 -1 2 4.33255 0.47185 1

1 -1 -2 3.44904 0.29519 6

1 -1 -2 3.86982 0.49090 3

1 -1 -2 4.20828 0.51309 1

1 -1 -2 4.02101 0.42661 7

-1 -1 2 4.78978 0.36953 2

-1 1 -2 4.73172 0.40376 2

-1 -1 2 4.17396 0.50923 8

1 1 2 4.03445 0.37029 4

1 1 2 3.43297 0.21052 7

1 1 2 4.33289 0.35654 2

-1 -1 1 13.7033 0.65433 1

-1 1 -1 13.6277 0.74036 1

1 1 1 13.4337 0.60494 3

1 -1 -1 13.8440 0.54979 7

1 -1 -1 12.9543 0.58068 3

1 1 1 12.5457 0.41943 2

1 -1 -1 12.5876 0.49385 6

1 1 1 13.3547 0.58148 6

1 -1 -1 13.5990 0.63263 5

1 -1 -1 12.9278 0.57193 4

1 -1 -1 12.9193 0.66516 1

-1 -1 1 13.4551 0.59261 6

1 1 1 13.5893 0.48210 4

-1 1 -1 13.4087 0.44511 2

-1 -1 1 13.4187 0.56065 8

1 -1 0 7.43161 0.44251 3

-1 -1 0 8.71049 0.58536 1

-1 -1 0 7.75071 0.45345 6

1 1 0 7.68339 0.49486 6

1 -1 0 8.00310 0.46128 7

-1 -1 0 8.42107 0.34833 8

-1 -1 0 7.25685 0.31485 4

1 1 0 9.97722 0.46850 8

1 -1 0 7.83826 0.46067 4

1 -1 0 9.21085 0.61655 1

-1 -1 0 7.05783 0.41580 3

-1 1 0 9.45150 0.63524 1

-1 1 1 13.4780 0.59123 5

1 1 -1 14.0130 0.62294 6

1 -1 1 13.0433 0.68906 1

-1 1 1 13.3699 0.74022 1

-1 -1 -1 11.8406 0.37828 7

-1 -1 -1 12.4176 0.55241 3

-1 -1 -1 13.7001 0.43435 2

-1 -1 -1 13.5204 0.53412 6

-1 1 1 14.5457 0.61498 3

-1 -1 -1 12.9681 0.63964 1

-1 1 1 13.2288 0.53578 7

1 -1 1 12.6821 0.41617 2

-1 -1 -1 12.4668 0.44972 4

1 1 -1 13.7110 0.69516 8

-1 1 1 14.7988 0.61280 4

1 1 -2 2.86229 0.69586 8

-1 -1 -2 4.08789 0.45474 3

-1 1 2 5.07470 0.52019 3

1 -1 2 4.33042 0.52961 1

-1 1 2 3.82546 0.36316 7

-1 -1 -2 3.68939 0.47864 1

-1 -1 -2 3.81413 0.36686 6

-1 1 2 3.95680 0.46454 5

-1 -1 -2 4.77646 0.39758 2

-1 1 2 3.91249 0.57690 1

-1 -1 -2 4.11589 0.44938 5

-1 1 2 4.50833 0.48489 4

-1 1 2 4.97206 0.37648 2

1 -1 2 4.30476 0.34176 2

-1 1 3 55.2594 1.48546 7

1 -1 3 55.1729 1.58230 1

-1 1 3 57.7713 1.59213 1

-1 1 3 55.5107 1.38760 6

-1 1 3 58.5048 1.55335 2

-1 1 3 56.3307 1.59322 5

-1 -1 -3 58.1649 1.58797 2

-1 -1 -3 54.3312 1.49027 6

1 1 -3 55.0375 1.82535 8

-1 1 3 56.3744 1.62305 3

1 -1 3 59.4836 1.54004 2

-1 1 3 58.1935 1.60338 4

-1 -1 -3 53.4935 1.55809 1

-1 1 4 2.04220 0.42470 1

-1 1 4 2.27165 0.53825 4

1 1 -4 1.36405 0.83214 8

-1 1 4 1.31257 0.47959 5

-1 -1 -4 1.33655 0.40668 1

1 -1 4 2.35173 0.49828 2

1 -1 4 2.09356 0.61418 8

1 -1 4 1.96593 0.43629 1

-1 1 4 1.43385 0.25028 6

-1 1 4 2.73758 0.41431 7

-1 1 4 1.63845 0.61010 3

-1 -1 -4 1.57550 0.54950 2

-1 1 4 1.27467 0.46330 2

1 -1 5 78.8722 2.50555 2

-1 1 5 108.904 2.67726 3

-1 1 5 92.7478 2.53963 2

-1 -1 -5 88.8234 2.36867 1

-1 1 5 98.4837 2.24944 6

-1 -1 -5 93.1674 2.65911 2

-1 1 5 104.939 2.70072 4

-1 1 5 87.7665 2.37657 1

1 1 -5 105.285 2.92138 8

1 -1 5 92.5676 2.65712 8

-1 1 5 96.2414 2.45538 7

-1 1 6 0.20996 0.55613 2

-1 1 6 0.79160 0.55109 7

-1 1 6 0.81619 0.73037 4

-1 1 6 0.47717 0.35545 1

1 -1 6 0.37631 0.61466 2

-1 1 6-0.04424 0.79379 3

1 1 -6 0.05801 0.99822 8

-1 -1 -6 0.91604 0.73915 2

-1 1 7 0.51797 0.65038 7

1 1 -7 0.86676 1.13046 8

-1 1 7 1.60989 0.92418 4

-1 1 7 1.55462 1.04544 3

-1 -1 -7 0.72154 0.88461 2

-1 1 8 70.3830 2.53694 4

-1 1 8 64.2169 2.63910 3

-1 -1 -8 79.5420 2.54424 2

-1 1 9 7.19351 1.32094 4

-1 1 9 2.20569 1.60574 3

-1 -1 -9 7.78190 1.26238 2

-1 1 10 0.16016 1.27192 4

-1 -1 -10 0.45319 1.16420 2

-1 1 10-1.43820 1.60950 3

-1 -1 -11 6.66550 1.36087 2

-1 1 11 7.91888 1.74103 3

-1 1 11 6.33891 1.53370 4

-1 1 12 32.2109 2.16883 3

-1 1 12 27.4249 2.03997 4

-1 1 13 1.07778 1.60268 4

-1 1 13 5.12470 1.65016 3

-1 1 14 2.13933 1.71466 4

1 -1 14 0.53430 1.60792 4

-1 1 14 2.93834 1.66823 3

2 1 14 8.61162 1.82530 3

2 1 14 5.52969 1.63713 4

2 1 13 2.57867 1.56155 4

2 1 13 9.17422 1.83668 3

-2 -1 12 10.4091 1.53581 4

2 1 12 12.2894 1.90234 3

2 1 12 9.06133 1.61927 4

-2 1 -12 8.37793 1.53848 2

2 1 11 1.49206 1.38514 4

-2 -1 11 0.84645 1.28667 4

2 1 11 1.21960 1.71691 3

-2 1 -11 0.88526 1.31901 2

2 1 10 15.7337 1.97310 3

-2 1 -10 13.2685 1.49156 2

-2 -1 10 18.3211 1.50309 4

2 1 10 16.3041 1.59913 4

2 1 9 19.5910 1.56076 4

-2 1 -9 14.6188 1.36427 2

2 1 9 5.25958 1.71777 3

-2 -1 9 17.3270 1.41624 4

2 -1 -8 0.60849 0.97013 8

2 1 8-1.76807 1.34365 3

2 1 8 1.46005 1.13855 4

-2 1 -8-0.78487 0.96855 2

2 -1 -7 10.5298 0.94188 8

-2 1 -7 9.32289 1.01813 2

2 1 7 8.86803 1.20420 3

2 1 7 8.27293 0.92984 6

2 1 7 9.94100 1.20743 4

-2 1 -6 4.44880 0.78917 2

-2 -1 6 4.99626 0.64478 6

2 -1 -6 4.15305 0.70888 8

2 1 6 5.11182 0.73846 6

2 1 6 5.47272 0.90112 3

2 1 6 4.39973 0.64410 5

2 1 6 3.60312 0.95737 4

2 1 5 4.21357 0.60990 2

-2 -1 5 4.65770 0.60128 6

-2 -1 5 4.85312 0.61260 2

2 1 5 4.70256 0.72587 3

-2 -1 5 4.29004 0.72297 8

2 1 5 3.99496 0.62744 6

-2 1 -5 4.88453 0.68549 2

2 -1 -5 4.60421 0.61822 8

-2 1 -5 3.99489 0.60246 1

-2 -1 5 4.09177 0.45999 1

2 1 5 3.29005 0.85683 4

2 1 5 5.27803 0.60096 5

2 1 5 4.75048 0.59926 8

2 -1 -5 4.11273 0.65937 4

2 1 4 0.40048 0.58275 3

-2 -1 4-0.03899 0.42886 2

2 1 4 0.23908 0.46221 8

2 1 4 0.20058 0.45831 2

-2 1 -4-0.43276 0.57038 1

2 1 4 0.15930 0.50373 6

2 1 4-0.43884 0.71501 4

-2 -1 4 0.37878 0.56214 8

-2 -1 4 0.04874 0.45407 6

-2 1 -4-0.19561 0.54155 2

2 1 4 0.30858 0.41374 5

2 -1 -4 0.44175 0.49171 8

2 -1 -4 0.22445 0.47099 4

-2 -1 4 0.12810 0.42476 1

2 -1 -3 1.96151 0.46745 4

2 1 3 0.25334 0.35029 8

2 -1 -3 0.03896 0.18045 6

2 1 3 0.40323 0.36266 2

-2 1 -3 0.17776 0.43765 2

-2 1 -3 0.39013 0.56148 1

2 1 3 0.13906 0.52525 4

2 1 3 0.24733 0.48214 3

-2 -1 3 5.76490 0.62758 1

2 -1 -3 1.57312 0.49404 7

2 1 3 0.38115 0.46374 6

2 1 2 266.441 6.20204 3

2 -1 -2 258.321 6.19564 3

-2 -1 2 261.112 6.21826 6

-2 -1 2 264.244 6.18571 8

2 1 2 264.198 6.22594 6

2 1 2 260.454 6.12880 2

2 1 2 267.520 6.17310 4

2 -1 -2 265.399 6.22334 4

-2 1 -2 264.556 6.16113 2

2 -1 -2 268.350 6.23325 7

-2 1 -2 279.006 6.40609 1

-2 1 -1 313.791 7.31448 2

-2 1 -1 323.315 7.60717 1

2 1 1 323.947 7.37182 3

2 -1 -1 321.039 7.44209 5

-2 -1 1 320.981 7.41054 6

2 1 1 325.029 7.42980 6

2 1 1 316.656 7.26602 4

2 -1 -1 319.235 7.42623 4

2 -1 -1 306.253 7.37368 3

2 -1 -1 328.189 7.43216 7

-2 1 0 1.39325 0.38583 7

-2 1 0 2.84032 0.66834 1

2 -1 0 1.12225 0.41671 7

2 -1 0 0.50512 0.41446 4

2 -1 0 0.04841 0.35993 3

-2 -1 0 1.83330 0.39995 6

-2 -1 0 0.44097 0.36948 3

2 -1 0 1.03421 0.40445 5

2 1 0 1.77208 0.45370 6

2 1 0 1.58166 0.32463 8

-2 1 1 314.082 7.72453 1

2 -1 1 329.735 7.60217 7

-2 1 1 334.670 7.55493 7

-2 -1 -1 315.589 7.51452 3

2 1 -1 330.083 7.51754 8

2 1 -1 323.650 7.59115 6

-2 -1 -1 320.741 7.54510 6

-2 1 1 337.014 7.56814 5

-2 -1 -1 329.272 7.46009 2

-2 1 1 313.347 7.50668 3

-2 1 1 324.742 7.59413 4

-2 1 2 256.894 6.33762 1

-2 1 2 254.091 6.16132 7

2 1 -2 267.332 6.22693 6

2 1 -2 265.089 6.23131 8

-2 -1 -2 271.275 6.18124 5

-2 1 2 265.458 6.18202 3

-2 -1 -2 268.304 6.18047 3

-2 1 2 256.091 6.19747 5

-2 -1 -2 263.829 6.17796 6

2 -1 2 260.804 6.09871 2

-2 -1 -2 262.897 6.14132 2

-2 1 2 273.499 6.24881 4

-2 1 3 1.78226 0.46878 5

-2 -1 -3 0.39645 0.44126 2

2 -1 3 0.54457 0.36244 2

-2 1 3 1.01569 0.51735 4

-2 1 3 4.40696 0.64537 1

-2 1 3 1.26363 0.52285 3

-2 -1 -3 0.35832 0.39214 5

-2 -1 -3 0.43232 0.53255 1

-2 1 3 1.42589 0.40190 7

-2 1 4-0.06221 0.49538 1

-2 1 4 0.55340 0.40402 7

2 -1 4 0.06358 0.45344 2

-2 1 4 0.49352 0.58463 3

-2 1 4 0.31571 0.54737 4

-2 -1 -4 0.40713 0.51684 2

-2 -1 -4 0.30037 0.50111 1

2 1 -4 0.13115 0.77206 8

-2 1 4 0.30255 0.43234 2

2 -1 5 4.86709 0.73013 8

-2 1 5 4.33155 0.60017 2

2 1 -5 4.27594 0.92865 8

-2 1 5 4.19884 0.52931 1

-2 1 5 4.55918 0.54817 7

-2 1 5 5.51842 0.72067 4

-2 1 5 4.88786 0.72571 3

-2 -1 -5 4.35016 0.71251 2

2 -1 5 4.78102 0.61049 2

-2 1 6 4.46705 0.62306 7

-2 1 6 4.64708 0.48454 1

-2 1 6 4.53681 0.85087 3

-2 -1 -6 5.10975 0.80536 2

2 1 -6 4.16651 1.06829 8

-2 1 6 5.01378 0.84144 4

2 1 -7 7.76258 1.30405 8

-2 -1 -7 7.49113 0.99384 2

-2 1 7 9.27480 1.15283 3

-2 1 7 10.8482 1.06550 4

-2 1 8 1.08849 1.01074 4

2 1 -8 0.64393 1.22699 8

-2 1 8-0.64565 1.27472 3

-2 -1 -8 0.09726 0.97534 2

-2 -1 -9 17.5794 1.43604 2

-2 1 9 6.14281 1.59756 3

2 1 -9 15.1157 1.59854 8

-2 1 9 16.1885 1.43418 4

2 1 -10 12.4128 1.65024 8

-2 -1 -10 16.3504 1.52254 2

-2 1 10 13.2624 1.80604 3

-2 1 10 15.1044 1.58833 4

-2 1 11 1.17394 1.41892 4

-2 -1 -11 1.67586 1.35826 2

-2 1 11 1.35922 1.55621 3

-2 1 12 11.0729 1.77210 3

-2 1 12 10.8929 1.76740 4

-2 -1 -12 9.68952 1.56333 2

-2 1 13 5.06322 1.63393 3

-2 1 13 2.36598 1.69691 4

-2 1 14 8.08954 1.80952 4

-2 1 14 6.02560 1.71802 3

-3 1 -14 5.46695 1.67466 2

3 1 14 5.76282 1.61927 4

3 1 14 8.43386 1.84858 3

3 1 13 6.12910 1.82864 3

3 1 13 1.48534 1.50663 4

-3 1 -13 4.10026 1.56376 2

3 1 12 2.68599 1.74339 3

-3 1 -12 0.61608 1.44233 2

3 1 12-0.44138 1.44420 4

3 1 11 17.0761 2.03834 3

-3 1 -11 13.3014 1.62168 2

3 1 11 12.0582 1.62311 4

3 1 10 1.65377 1.33636 4

-3 -1 10 2.10604 1.16478 4

3 1 10 0.03387 1.73880 3

-3 1 -10 0.84015 1.21225 2

3 -1 -9 27.8468 1.56882 8

-3 1 -9 28.3763 1.62978 2

3 1 9 11.9287 1.85634 3

3 1 9 33.4669 1.79994 4

-3 -1 9 29.7040 1.60324 4

3 -1 -8 2.26323 0.95852 8

-3 1 -8 0.93942 0.94309 2

3 1 8-1.63949 1.09190 6

3 1 8 0.28678 1.29500 3

-3 -1 8 1.00005 0.99290 4

-3 1 -7 36.3257 1.59428 2

3 1 7 36.7824 1.63568 6

3 -1 -7 36.8026 1.50346 8

3 1 7 41.2578 1.78890 3

-3 -1 7 40.3201 1.62678 4

-3 1 -6 0.69687 0.75394 2

-3 -1 6 0.02784 0.75403 4

3 -1 -6 0.96246 0.72217 8

-3 -1 6 1.23361 0.56133 6

3 1 6 0.51976 0.83479 3

3 1 6 0.50785 0.77960 6

3 -1 -5 8.22584 0.74642 8

3 1 5 8.59361 0.82149 6

-3 -1 5 7.44299 0.63980 2

3 1 5 7.65581 0.66746 8

-3 -1 5 7.43140 0.76480 4

-3 1 -5 9.06399 0.77286 1

3 1 5 8.52380 0.83057 3

-3 -1 5 8.87818 0.66385 6

3 1 5 8.77467 0.65426 2

-3 1 -5 8.87969 0.79208 2

-3 1 -4 160.175 3.75935 2

3 1 4 146.720 3.73709 6

3 -1 -4 153.438 3.79916 7

3 1 4 144.816 3.71555 3

-3 -1 4 149.125 3.64131 6

-3 1 -4 158.044 3.81299 1

-3 -1 4 147.411 3.71098 8

-3 -1 4 142.706 3.63900 4

3 1 4 141.794 3.61403 2

3 -1 -4 132.070 3.67585 4

3 1 3 9.00672 0.33884 7

3 1 3 10.6039 0.66776 3

-3 1 -3 12.6991 0.87576 1

-3 -1 3 10.9342 0.62054 6

3 1 3 10.7338 0.71704 6

3 -1 -3 10.6408 0.71330 7

3 1 3 10.7295 0.57598 2

-3 -1 3 10.1181 0.52015 4

3 -1 -3 10.0344 0.65005 4

-3 1 -3 10.9645 0.66215 2

-3 1 -2 69.8911 2.02341 1

3 -1 -2 64.0697 1.82306 7

3 -1 -2 61.5956 1.70089 3

3 -1 -2 67.2255 1.79723 4

-3 -1 2 63.3039 1.74741 6

3 1 2 66.1748 1.83483 6

3 1 2 64.3200 1.69105 2

-3 -1 2 63.8565 1.56126 4

-3 1 -2 65.4365 1.72931 2

3 1 2 67.4222 1.73435 3

3 1 1 5.79583 0.56349 6

3 -1 -1 7.06661 0.62404 7

-3 -1 1 5.52304 0.49875 6

3 -1 -1 8.79725 0.51703 3

3 1 1 6.11429 0.46537 3

-3 1 -1 5.76609 0.43511 2

3 -1 -1 7.74008 0.59768 4

-3 -1 0 135.277 3.17453 3

3 -1 0 125.501 3.16261 3

-3 1 0 137.240 3.29357 7

3 -1 0 139.017 3.32692 7

-3 -1 0 130.018 3.12312 7

-3 -1 0 134.800 3.26898 6

3 1 0 133.077 3.30274 6

3 -1 0 138.425 3.23065 5

-3 1 0 130.265 3.20026 5

3 -1 0 131.188 3.27411 4

3 1 -1 5.48860 0.48518 8

3 -1 1 6.11354 0.57517 7

-3 1 1 6.37336 0.52992 7

-3 -1 -1 6.34184 0.52293 6

-3 -1 -1 5.61427 0.44438 3

3 1 -1 6.03615 0.54286 6

-3 -1 -1 4.56004 0.25394 7

-3 1 1 6.48787 0.46353 3

-3 1 1 6.45299 0.48542 5

3 -1 1 6.43781 0.47431 5

-3 -1 -1 5.74885 0.44264 2

-3 1 1 6.45355 0.59964 4

-3 1 2 68.4402 2.03252 1

-3 -1 -2 63.6560 1.70205 3

3 1 -2 65.2704 1.82458 6

3 1 -2 66.1102 1.79172 8

3 -1 2 63.9255 1.85065 7

-3 -1 -2 65.5310 1.78136 6

-3 1 2 62.1465 1.75149 5

-3 1 2 63.8911 1.75625 7

-3 1 2 65.2355 1.72793 3

3 -1 2 62.6122 1.67367 2

-3 -1 -2 65.8006 1.72203 2

-3 1 2 64.0077 1.82839 4

3 1 -3 10.7549 0.75737 8

-3 1 3 11.0530 0.83282 1

-3 1 3 9.64579 0.63936 3

3 1 -3 10.8369 0.70640 6

-3 -1 -3 10.7518 0.65391 2

-3 -1 -3 10.3150 0.58967 5

3 -1 3 10.9298 0.57301 2

-3 1 3 10.2947 0.62757 7

-3 1 3 10.3505 0.72603 4

-3 -1 -4 141.158 3.60582 2

3 1 -4 147.357 3.70175 8

-3 1 4 140.413 3.63251 4

-3 1 4 142.362 3.52385 7

-3 1 4 146.598 3.63486 1

3 -1 4 142.537 3.50232 2

-3 1 4 132.357 3.57387 3

3 1 -5 9.06603 0.94790 8

-3 1 5 8.28339 0.65311 7

-3 1 5 8.41852 0.81821 3

-3 1 5 8.20637 0.64423 2

-3 1 5 8.62446 0.69542 1

-3 -1 -5 9.24801 0.79622 2

3 -1 5 7.53241 0.65162 2

-3 1 5 8.80504 0.81559 4

-3 1 6 0.20411 0.76555 3

-3 -1 -6 0.24935 0.72608 2

-3 1 6 0.45567 0.52046 1

3 1 -6 0.82621 0.97683 8

-3 1 6 1.36524 0.76979 4

3 1 -7 37.8760 1.84845 8

-3 -1 -7 37.9469 1.59425 2

-3 1 7 38.1892 1.69713 3

-3 1 7 41.5923 1.69564 4

-3 -1 -8 0.46506 0.94999 2

-3 1 8 1.72992 1.10110 4

-3 1 8 0.51868 1.18947 3

3 1 -8 1.73780 1.27878 8

-3 -1 -9 27.8441 1.60979 2

-3 1 9 18.0868 1.81035 3

3 1 -9 27.5873 1.85898 8

-3 1 9 30.1193 1.73338 4

3 1 -10 0.40604 1.48190 8

-3 1 10-0.49368 1.55492 3

-3 1 10-0.62578 1.36502 4

-3 -1 -10 1.00369 1.23856 2

-3 -1 -11 14.5984 1.62254 2

3 1 -11 14.9678 1.89236 8

-3 1 11 12.5247 1.77004 3

-3 1 11 14.0556 1.72158 4

-3 1 12 2.57682 1.59222 3

-3 1 12 1.35255 1.58949 4

-3 -1 -12 1.07575 1.42833 2

-3 1 13 4.53956 1.63244 3

-3 -1 -13 3.64752 1.60308 2

-3 1 13 3.65866 1.73808 4

-3 1 14 7.31907 1.76564 3

-3 -1 -14 6.18755 1.66879 2

4 1 13 7.79467 1.64326 4

4 1 13 11.2188 1.93246 3

-4 1 -13 9.46192 1.75072 2

4 1 12 1.86401 1.47469 4

4 1 12 2.78366 1.76508 3

-4 1 -12 1.41865 1.59561 2

4 1 11-0.63723 1.35044 4

4 1 11 0.03327 1.73799 3

-4 1 -11 1.51198 1.37019 2

4 1 10 3.32902 1.33555 4

4 1 10-0.09662 1.73886 3

-4 1 -10 2.30384 1.23562 2

4 -1 -9 1.95386 1.14007 8

4 1 9-1.52144 1.49851 3

-4 1 -9 0.44565 1.08990 2

4 -1 -8 42.0350 1.70658 8

-4 1 -8 44.2499 1.81467 2

4 1 8 42.9590 1.98750 3

4 1 8 25.0002 1.81858 6

-4 -1 7 5.28134 0.86203 4

4 -1 -7 7.16364 0.99028 8

4 1 7 8.26376 1.17978 6

-4 1 -7 7.26992 0.98329 2

4 1 7 7.41787 1.13581 3

-4 1 -6 34.3941 1.45192 2

4 -1 -6 34.0730 1.41531 8

4 1 6 34.4372 1.48590 3

4 1 6 32.5104 1.53272 6

-4 -1 6 35.9646 1.33605 4

-4 -1 5 1.10834 0.54861 4

4 -1 -5 1.17991 0.73108 8

-4 -1 5-0.05469 0.50453 6

-4 1 -5 0.63940 0.70043 2

4 1 5-0.17162 0.69343 3

4 1 5 0.64670 0.77900 6

-4 1 -5 0.55315 0.66472 1

4 -1 -4 22.9772 1.11701 7

4 1 4 23.2996 1.09900 6

-4 -1 4 21.9625 0.87536 6

4 1 4 20.0268 0.68209 7

4 1 4 21.3778 0.87081 2

4 -1 -4 21.3236 0.95229 4

4 1 4 21.6191 0.97147 3

-4 -1 4 21.8499 0.80250 4

-4 1 -4 21.2024 0.99481 2

4 -1 -3 210.776 4.82504 7

4 1 3 180.818 4.42312 7

4 1 3 198.820 4.62827 3

-4 -1 3 194.565 4.63576 6

4 1 3 211.694 4.82237 6

4 1 3 190.985 4.62415 2

-4 -1 3 188.548 4.49606 4

4 -1 -3 191.712 4.70090 4

-4 1 -3 198.463 4.70717 2

-4 -1 2 26.3905 0.97953 7

4 1 2 29.5852 1.12128 6

4 1 2 28.3303 0.77240 7

4 -1 -2 29.6429 1.11610 7

-4 -1 2 29.2247 1.00026 6

4 -1 -2 27.4385 0.90965 3

4 1 2 27.7613 0.93787 3

-4 1 -2 28.3678 0.99121 2

4 -1 -2 29.1104 1.04191 4

-4 -1 2 27.9856 0.78136 4

-4 -1 1 0.77332 0.36339 3

-4 1 -1 0.24085 0.41155 2

4 -1 -1 0.50583 0.54171 7

-4 -1 1 0.86483 0.37368 7

4 -1 -1 1.33831 0.48342 4

4 1 1 1.05009 0.54914 6

-4 -1 1 1.07119 0.47908 6

4 1 1 0.94476 0.38748 3

4 -1 -1 0.92042 0.37923 3

4 -1 0 0.22322 0.33386 3

4 1 0 0.36260 0.19308 4

4 -1 0 0.42174 0.47507 4

-4 1 0 0.10173 0.46584 7

-4 -1 0 0.21959 0.45169 6

4 -1 0 0.56663 0.51919 7

-4 -1 0 0.26013 0.34671 3

-4 -1 0 0.23875 0.29694 7

4 1 0 0.34687 0.52227 6

-4 1 0 0.22084 0.36545 5

-4 -1 -1 0.83554 0.39386 3

4 -1 1 1.00355 0.54160 7

-4 1 1 1.23366 0.57598 4

4 -1 1 0.39798 0.35237 3

-4 1 1 1.16306 0.47143 7

4 1 -1 1.11662 0.55507 6

-4 -1 -1 0.84931 0.47570 6

-4 -1 -1 0.65153 0.42002 2

-4 1 1 1.26587 0.40171 5

-4 -1 -1 0.46512 0.26717 7

-4 1 1 0.93616 0.38545 3

4 1 -2 28.9109 1.06868 8

-4 1 2 29.7859 1.05503 7

4 -1 2 27.4777 1.10891 7

-4 1 2 28.9909 0.95336 3

-4 -1 -2 27.7275 0.93629 3

4 1 -2 28.5536 1.09284 6

4 -1 2 30.4423 0.90662 5

-4 -1 -2 29.7197 1.02295 2

-4 1 2 30.1636 1.12786 4

4 1 -3 196.188 4.82075 8

-4 1 3 193.044 4.69290 3

4 1 -3 198.551 4.79536 6

-4 1 3 200.044 4.76560 7

-4 -1 -3 191.903 4.65747 5

4 -1 3 187.374 4.58853 5

4 -1 3 198.933 4.88807 7

4 -1 3 191.677 4.68914 2

-4 -1 -3 197.801 4.78251 2

-4 1 3 211.654 4.88155 4

4 1 -4 22.5607 1.09237 8

-4 1 4 22.0586 0.97353 3

4 1 -4 23.4505 1.01341 6

-4 1 4 22.9984 0.94223 7

4 -1 4 20.4858 0.86603 2

-4 1 4 23.5095 1.08963 4

-4 -1 -4 20.8582 0.99571 2

4 1 -5 0.17324 0.80020 8

-4 -1 -5-0.22937 0.67472 2

-4 1 5 0.23242 0.68762 3

-4 1 5 0.20411 0.70030 4

-4 1 5 0.55029 0.64084 1

4 1 -6 38.2757 1.63954 8

-4 -1 -6 32.0163 1.41872 2

-4 1 6 30.8801 1.24280 1

-4 1 6 35.0722 1.45938 4

-4 1 6 31.6480 1.40259 3

-4 -1 -7 6.75234 0.99200 2

-4 1 7 7.14184 1.03467 3

4 1 -7 8.01964 1.19441 8

-4 1 7 8.23516 1.07831 4

-4 1 8 43.3597 1.95726 3

-4 -1 -8 45.4249 1.88224 2

4 1 -8 42.9521 2.10888 8

-4 1 8 48.5901 1.96524 4

-4 1 9 1.61064 1.21350 4

-4 1 9-0.29169 1.32188 3

-4 -1 -9 0.04410 1.09681 2

4 1 -9-0.66195 1.41589 8

4 1 -10 3.50412 1.62701 8

-4 1 10 2.71248 1.39929 4

-4 1 10-0.32773 1.49698 3

-4 -1 -10 2.33039 1.22630 2

4 1 -11-0.78144 1.69080 8

-4 1 11-0.79487 1.51431 4

-4 1 11-1.14696 1.52887 3

-4 -1 -11-1.15938 1.32883 2

-4 1 12 2.38406 1.67141 4

4 1 -12 2.47762 1.88324 8

-4 1 12 3.50990 1.59940 3

-4 -1 -12 1.92375 1.53456 2

-4 1 13 11.2304 1.79523 3

-4 1 13 10.7020 1.93573 4

-4 -1 -13 8.96939 1.77622 2

-4 1 14 5.00010 1.74569 3

5 1 13 8.13766 1.62519 4

-5 1 -13 8.32148 1.81165 2

5 1 12 0.97314 1.47357 4

-5 1 -12 0.84868 1.49476 2

5 1 11 3.51168 1.43912 4

5 1 11 2.16818 1.79947 3

-5 1 -11 0.69398 1.40117 2

5 1 10 1.88019 1.70013 3

-5 1 -10 7.89685 1.39582 2

5 -1 -10 10.1928 1.46972 8

5 -1 -9 5.78645 1.24347 8

5 1 9 2.21487 1.40627 3

5 1 9 3.11269 1.48423 6

-5 1 -9 4.47632 1.19319 2

5 -1 -8 4.45553 1.13788 8

-5 1 -8 1.92975 1.10392 2

5 1 8 0.16146 1.37266 6

5 1 8 2.97774 1.24910 3

-5 1 -7 52.9935 2.02259 2

5 -1 -7 57.4517 2.02397 8

5 1 7 55.5458 2.07990 3

5 1 7 48.0701 2.22083 6

-5 1 -6 0.92316 0.83887 2

5 1 6 1.98640 0.90178 3

5 -1 -6 1.53156 0.90366 8

5 1 6 1.47596 1.03977 6

5 1 5 1.54171 0.74280 3

-5 1 -5 1.33562 0.74415 2

5 1 5 1.59571 0.91455 6

5 -1 -4 13.3842 1.04670 7

5 1 4 11.9387 0.77658 3

5 1 4 12.3154 0.98825 6

-5 1 -4 14.5213 0.90520 2

-5 -1 4 12.3553 0.74410 6

-5 -1 3-0.27820 0.51278 6

-5 -1 3 0.34546 0.51031 7

5 1 3-0.25764 0.30196 7

-5 1 -3 0.28245 0.60070 2

5 1 3 0.27386 0.49814 3

5 1 3-0.21808 0.68804 6

5 -1 -3 0.12308 0.66540 7

5 1 2 56.7194 1.50298 3

5 1 2 56.2335 1.78245 6

-5 -1 2 48.3271 1.55616 7

5 1 2 54.4226 1.41742 7

5 -1 -2 58.6315 1.77190 7

-5 -1 2 57.1030 1.63377 6

-5 1 -2 54.7388 1.64671 2

5 -1 -2 53.1707 1.65112 4

-5 -1 1 36.6583 1.11207 7

5 -1 -1 38.1946 1.35884 7

5 1 1 34.2933 1.05379 3

-5 1 -1 29.6353 1.20759 7

-5 -1 1 33.1989 1.02800 3

5 -1 -1 35.5232 1.06640 3

-5 -1 1 40.9862 1.24005 6

5 1 1 38.1312 1.37045 6

-5 1 -1 29.6135 1.17993 2

5 -1 -1 35.7208 1.26360 4

-5 1 0 436.226 9.72562 7

-5 -1 0 419.407 9.48468 3

5 -1 0 420.714 9.75651 7

5 1 0 431.709 9.74773 6

-5 -1 0 432.589 9.67631 6

-5 1 0 397.331 9.55952 5

-5 -1 0 411.871 9.52948 7

5 -1 0 407.783 9.47610 3

5 1 0 403.382 9.42704 4

5 -1 0 417.663 9.67536 4

-5 -1 -1 36.6280 1.10056 3

5 -1 1 30.3348 1.01709 3

5 -1 1 29.3008 1.25600 7

5 1 -1 43.6583 1.37584 6

-5 1 1 37.8911 1.25386 7

-5 -1 -1 35.5689 1.23933 6

-5 1 1 32.6563 1.09821 5

-5 -1 -1 31.6323 1.20432 2

5 1 -1 34.9448 0.91804 4

5 -1 1 31.7986 1.23990 4

5 1 -2 56.1871 1.71712 8

5 1 -2 55.3136 1.73471 6

-5 1 2 54.4668 1.68606 7

5 -1 2 52.7061 1.76340 7

-5 1 2 55.3475 1.52571 3

-5 -1 -2 57.7972 1.68674 2

-5 1 2 59.2834 1.80293 4

-5 -1 -3-0.01090 0.57322 2

-5 1 3-0.05055 0.63507 4

5 -1 3-0.05409 0.66495 7

-5 1 3 0.07160 0.50893 3

5 1 -3 0.03396 0.62909 6

-5 -1 -3-0.14159 0.42220 5

5 1 -3 0.38393 0.66591 8

5 1 -4 12.2707 0.97553 8

5 -1 4 12.5388 0.93248 7

-5 1 4 12.2508 0.76552 3

5 1 -4 12.3959 0.86237 6

-5 -1 -4 12.8101 0.87422 2

-5 1 4 14.8361 0.95231 4

5 1 -5 1.28572 0.86265 8

-5 1 5 0.77254 0.77059 4

-5 1 5 1.54725 0.71188 3

-5 -1 -5 1.67576 0.76451 2

5 1 -6 1.69210 0.96756 8

-5 1 6 1.69111 0.89518 4

-5 -1 -6-0.11665 0.83779 2

-5 1 6 1.76226 0.85175 3

-5 1 6 2.13265 0.72682 1

5 1 -7 53.6536 2.12457 8

-5 -1 -7 52.6834 1.97789 2

-5 1 7 50.3857 1.95203 3

-5 1 7 55.4331 2.00688 4

-5 1 8 3.16958 1.10626 4

5 1 -8 4.36144 1.33957 8

-5 -1 -8 1.68018 1.08997 2

-5 1 8 3.36309 1.16725 3

-5 1 9 4.47048 1.29193 4

-5 1 9 3.39977 1.35260 3

-5 -1 -9 3.31872 1.20567 2

5 1 -9 3.42599 1.49920 8

-5 1 10 9.21070 1.53785 4

-5 1 10 2.79250 1.45648 3

-5 -1 -10 7.86830 1.36571 2

5 1 -10 6.83446 1.71955 8

5 1 -11 2.27169 1.84739 8

-5 1 11 2.41172 1.55367 4

-5 1 11-0.08599 1.55986 3

-5 -1 -11 0.74226 1.36684 2

5 1 -12 0.72543 1.89661 8

-5 1 12 1.58474 1.65975 4

-5 -1 -12 1.30814 1.48391 2

-5 1 12-0.02957 1.60850 3

5 1 -13 10.9946 2.18807 8

-5 1 13 12.4172 1.96026 4

-5 -1 -13 12.0591 1.86875 2

6 1 13 5.57352 1.60354 4

-6 1 -13 5.38655 1.72999 2

6 1 12 0.39667 1.41980 4

-6 1 -12 1.51026 1.52614 2

-6 1 -11 13.0079 1.65250 2

6 -1 -10 43.3225 2.10774 8

-6 1 -10 40.3823 2.09328 2

6 -1 -9 13.9875 1.46212 8

-6 1 -9 12.7032 1.47933 2

6 1 9 12.0502 1.84498 6

6 -1 -8 0.78617 1.16596 8

-6 1 -8 0.42940 1.08780 2

6 1 8 0.08955 1.45644 6

-6 1 -7 31.1784 1.63355 2

6 -1 -7 33.6709 1.66660 8

6 1 7 30.0028 1.88547 6

-6 1 -6 2.13527 0.94765 2

6 1 6 2.22537 1.18107 6

6 -1 -6 2.05383 1.04488 8

6 -1 -5 0.92146 0.97604 7

-6 1 -5 0.94963 0.81530 2

6 1 5 0.42205 0.97417 6

-6 -1 4 49.0623 1.56101 6

6 1 4 46.6616 1.85575 6

6 -1 -4 49.7814 1.88646 7

-6 1 -4 48.9821 1.72644 2

6 -1 -3 4.00660 0.83699 7

-6 -1 3 4.57367 0.63706 6

-6 -1 3 4.53414 0.59354 7

6 1 3 5.53512 0.89367 6

-6 1 -3 5.75535 0.73849 2

6 1 2 0.47622 0.41137 7

-6 1 -2 0.15302 0.61519 2

-6 -1 2 0.69583 0.47814 7

6 -1 -2 0.56821 0.70902 7

6 1 2 0.79589 0.72917 6

-6 -1 2 0.73167 0.55675 6

6 1 1 1.45360 0.45922 7

-6 1 -1 2.20344 0.62637 2

6 1 1 1.30212 0.69093 6

6 -1 -1 1.54306 0.71976 7

6 -1 0 0.04228 0.65857 7

6 1 0 0.55477 0.67198 6

-6 -1 -1 1.43720 0.61310 2

6 -1 1 1.22261 0.65903 7

6 1 -1 0.94736 0.64922 6

-6 1 2-0.05570 0.69160 4

-6 -1 -2 0.83482 0.63974 2

6 -1 2 0.17131 0.66942 7

-6 1 2 0.55190 0.43154 3

6 1 -2 0.50192 0.69994 6

-6 1 3 4.38244 0.81448 4

-6 -1 -3 5.58298 0.72879 2

-6 1 3 4.37075 0.55926 3

6 1 -3 4.92771 0.84378 8

6 1 -3 4.83052 0.77409 6

6 -1 3 4.93087 0.80625 7

6 1 -4 46.8179 1.75962 8

6 -1 4 47.1673 1.77790 7

-6 1 4 49.1166 1.51459 3

6 1 -4 48.3537 1.68000 6

-6 -1 -4 48.7276 1.71484 2

-6 1 4 51.6374 1.75297 4

6 1 -5 0.76071 0.97061 8

6 -1 5 0.18772 0.82501 7

-6 1 5 1.66592 0.86128 4

-6 -1 -5-0.19311 0.84082 2

6 1 -5 1.15587 0.73805 6

-6 1 5 0.72824 0.66928 3

6 1 -6 2.40979 1.01897 8

-6 1 6 2.62667 0.92603 4

-6 1 6 2.48218 0.85515 3

6 -1 6 1.15601 0.92812 7

-6 -1 -6 0.77485 0.90533 2

-6 -1 -7 30.5503 1.60438 2

6 1 -7 31.5906 1.73097 8

-6 1 7 33.0515 1.60612 4

6 1 -8 2.74507 1.34974 8

-6 1 8 1.06135 1.08247 4

-6 -1 -8 0.60568 1.11215 2

6 1 -9 13.7037 1.66336 8

-6 -1 -9 13.6554 1.44736 2

-6 1 9 16.0044 1.50021 4

6 1 -10 40.2605 2.41154 8

-6 -1 -10 39.3824 2.06776 2

-6 1 10 43.3552 2.19160 4

6 1 -11 13.5548 2.11485 8

-6 -1 -11 12.2993 1.65149 2

-6 1 11 15.0186 1.76875 4

6 1 -12 0.84279 2.04431 8

-6 1 12 0.68871 1.62995 4

-6 -1 -12 1.88397 1.54156 2

-6 1 13 4.28129 1.80742 4

-6 -1 -13 8.24430 1.75158 2

-7 1 -12-0.25350 1.59751 2

-7 1 -11 2.63979 1.53936 2

7 -1 -10-0.69705 1.45276 8

-7 1 -10-0.98506 1.41857 2

7 -1 -9 1.15434 1.35810 8

-7 1 -9 0.02634 1.30870 2

7 1 9 0.03536 1.74528 6

7 -1 -8 5.36168 1.32946 8

-7 1 -8 3.51474 1.19868 2

7 1 8 0.06470 1.57076 6

-7 1 -7 16.6911 1.41021 2

7 -1 -7 17.5328 1.44083 8

7 1 7 14.1192 1.66172 6

7 -1 -6 112.002 3.33277 8

7 1 6 115.436 3.57928 6

-7 1 -6 107.441 3.29594 2

-7 1 -5 0.07101 0.93271 2

7 -1 -5 2.03530 1.04564 7

7 1 5 3.23473 1.16407 6

7 -1 -4 65.4927 2.32555 7

7 1 4 64.5152 2.31749 6

-7 1 -4 66.4217 2.15945 2

7 1 3 53.1710 1.95210 6

7 -1 -3 50.8302 1.92779 7

-7 1 -3 54.3722 1.80687 2

-7 1 -2 0.73373 0.75942 2

7 -1 -2 1.41083 0.84327 7

7 1 2 0.84558 0.83184 6

7 -1 -1 17.4376 1.08370 7

7 1 1 17.4993 0.88147 7

7 1 1 19.3929 1.16837 6

-7 1 -1 24.9069 1.13585 2

7 -1 0 11.6333 0.93971 7

7 1 0 11.9300 0.95927 6

7 1 -1 16.7106 1.08863 6

-7 -1 -1 19.8505 1.05814 2

7 -1 1 23.7523 1.18146 7

-7 -1 -2 0.46101 0.77335 2

7 1 -2 0.73386 0.77316 6

7 -1 2-0.04640 0.74913 7

7 1 -3 52.7604 1.86395 8

7 -1 3 50.0346 1.86408 7

7 1 -3 52.4624 1.82097 6

-7 -1 -3 52.4528 1.79096 2

-7 1 3 52.3901 1.84737 4

7 1 -4 66.3364 2.23794 8

7 -1 4 65.2524 2.21306 7

7 1 -4 66.1451 2.13713 6

-7 -1 -4 68.4218 2.19954 2

-7 1 4 68.9880 2.21343 4

7 -1 5 1.40063 0.92499 7

-7 1 5 1.26425 0.90142 4

-7 -1 -5 0.61872 0.92883 2

7 1 -5 1.41050 1.04433 8

7 1 -6 110.537 3.30539 8

7 -1 6 100.302 3.24348 7

-7 1 6 111.391 3.20102 4

-7 -1 -6 107.456 3.23283 2

-7 -1 -7 15.7691 1.36679 2

7 1 -7 15.7179 1.46760 8

7 -1 7 15.3389 1.32910 7

-7 1 7 15.1498 1.29766 4

7 1 -8 5.02812 1.37294 8

-7 1 8 4.85069 1.22805 4

7 -1 8 2.97214 1.19028 7

-7 -1 -8 3.93015 1.23008 2

-7 1 9 0.32432 1.25246 4

7 -1 9-0.22771 1.22595 7

-7 -1 -9-0.36722 1.27587 2

7 1 -9 2.63342 1.54863 8

-7 1 10 1.10318 1.40256 4

-7 -1 -10-0.02801 1.39098 2

7 1 -10 0.82606 1.73807 8

7 1 -11 2.53639 2.01032 8

-7 1 11 3.23543 1.58635 4

-7 -1 -11 1.85681 1.45149 2

7 1 -12-0.67273 2.13580 8

-7 1 12 1.34287 1.68452 4

-7 -1 -12-0.53554 1.61012 2

-8 1 -11 6.40359 1.62438 2

8 1 10 3.48614 1.87532 6

8 -1 -10 1.05806 1.54360 8

-8 1 -10-0.67603 1.47621 2

8 -1 -9 11.3986 1.61671 8

-8 1 -9 9.12952 1.54053 2

8 1 9 4.29038 1.92206 6

8 -1 -8 1.18820 1.36124 8

-8 1 -8 0.43400 1.22220 2

8 1 8-2.04483 1.70557 6

-8 1 -7 2.61715 1.18177 2

8 -1 -7 7.42158 1.41486 8

8 1 7 3.70918 1.55601 6

8 1 6 24.2192 1.78063 6

-8 1 -6 20.3430 1.47143 2

8 -1 -5 8.27904 1.30377 7

-8 1 -5 7.54244 1.14040 2

8 1 5 9.79034 1.39023 6

8 -1 -4 67.1865 2.44061 7

8 1 4 69.3385 2.49516 6

-8 1 -4 64.6793 2.24971 2

8 -1 -3 4.48814 1.03500 7

-8 1 -3 4.88230 0.90837 2

8 1 3 7.50601 1.12508 6

-8 1 -2 1.75174 0.84489 2

8 1 2 2.33479 0.97628 6

8 -1 -2 2.24458 0.92975 7

-8 1 -1 2.31036 0.87255 2

8 -1 -1 1.39065 0.88100 7

-8 -1 0-1.37280 0.87823 2

-8 1 0-1.37102 0.88929 2

8 -1 0 1.23982 0.86814 7

8 1 0 0.97331 0.86516 6

8 -1 1 3.07599 0.91235 7

8 1 -1 1.62972 0.87712 6

-8 -1 -1 5.42523 0.96838 2

8 -1 2 1.68549 0.84999 7

-8 -1 -2 2.05577 0.87321 2

8 1 -2 1.20190 0.86176 6

8 1 -3 4.64724 1.00424 8

-8 -1 -3 3.67950 0.89824 2

8 -1 3 5.16984 0.96944 7

8 1 -3 3.87129 0.94453 6

8 1 -4 67.3617 2.35680 8

8 -1 4 66.4178 2.31778 7

-8 -1 -4 67.9511 2.28279 2

-8 1 4 70.1787 2.32761 4

8 1 -5 10.0173 1.30430 8

-8 -1 -5 7.35018 1.16386 2

-8 1 5 8.68479 1.11870 4

8 -1 5 7.72867 1.10016 7

8 1 -6 25.6329 1.64311 8

8 -1 6 21.2228 1.45008 7

-8 -1 -6 22.5402 1.51081 2

-8 1 6 23.2539 1.40881 4

-8 1 7 4.11964 1.16302 4

-8 -1 -7 3.58055 1.19735 2

8 -1 7 3.87236 1.19796 7

8 1 -7 5.01025 1.46023 8

8 -1 8-0.44155 1.19031 7

-8 1 8 2.13645 1.21862 4

8 1 -8 0.10623 1.40356 8

-8 -1 -8-0.12760 1.24048 2

8 1 -9 13.9277 1.85054 8

-8 -1 -9 8.78879 1.52232 2

-8 1 9 10.9048 1.46955 4

8 1 -10 1.66936 1.75671 8

-8 1 10 1.34192 1.47882 4

-8 -1 -10-1.14559 1.48667 2

8 1 -11 6.23090 2.11046 8

-8 -1 -11 5.37038 1.61284 2

-8 1 11 4.65082 1.62569 4

9 -1 -11 3.17436 1.76753 8

-9 1 -11 0.55256 1.64865 2

9 -1 -10 0.60748 1.66159 8

-9 1 -10 0.28090 1.55259 2

9 1 10 1.51972 1.99483 6

9 -1 -9 7.73514 1.71468 8

-9 1 -9 5.46709 1.56824 2

9 1 9 1.74832 1.96030 6

9 -1 -8 12.0013 1.64460 8

-9 1 -8 8.18271 1.48592 2

9 1 8 6.63658 1.90136 6

9 -1 -7-0.54825 1.35863 8

-9 1 -7 0.39960 1.24234 2

9 1 7 1.85084 1.65872 6

-9 1 -6 4.98523 1.28713 2

9 1 6 7.98001 1.62848 6

9 -1 -5 4.73962 1.30473 7

-9 1 -5 2.54455 1.16678 2

9 1 5 3.32136 1.40682 6

9 -1 -4 3.38057 1.22651 7

9 1 4 6.10435 1.30126 6

-9 1 -4 5.24838 1.09249 2

9 -1 -3 45.5580 1.98532 7

9 1 3 44.2084 2.00746 6

-9 1 -3 44.9835 1.84399 2

9 1 2 25.3422 1.50744 6

-9 1 -2 21.9799 1.40245 2

9 -1 -2 28.2975 1.52764 7

9 1 1 88.0067 2.76562 6

-9 1 -1 83.8015 2.67933 2

9 -1 -1 91.7235 2.80392 7

9 -1 0 42.1917 1.76848 7

9 1 0 45.6861 1.78161 6

-9 -1 0 43.2118 1.77461 2

-9 1 0 38.5583 1.75070 2

9 -1 1 88.3450 2.77448 7

9 1 -1 86.6670 2.74642 6

-9 -1 -1 93.7805 2.75563 2

9 1 -2 23.5446 1.37002 6

9 -1 2 25.7223 1.44979 7

-9 -1 -2 24.0698 1.43165 2

9 1 -3 45.3147 1.89256 8

9 -1 3 46.8616 1.86024 7

9 1 -3 44.2482 1.81514 6

-9 -1 -3 47.0765 1.88146 2

9 1 -4 5.11873 1.21556 8

9 -1 4 4.41289 1.07954 7

-9 -1 -4 5.24253 1.11742 2

-9 1 4 4.18720 1.06303 4

9 1 -5 3.47484 1.27384 8

-9 1 5 3.60051 1.10032 4

9 -1 5 3.34862 1.11936 7

-9 -1 -5 2.64938 1.14226 2

9 1 -6 8.20441 1.43716 8

9 -1 6 8.10826 1.21549 7

-9 1 6 6.07859 1.20232 4

-9 -1 -6 5.43542 1.28049 2

9 1 -7 1.75379 1.45593 8

-9 1 7 0.57413 1.12393 4

-9 -1 -7 0.20069 1.21750 2

9 -1 7 2.25491 1.17524 7

9 1 -8 9.61573 1.67406 8

-9 -1 -8 8.75652 1.48794 2

-9 1 8 10.5756 1.38355 4

9 1 -9 7.58228 1.74744 8

-9 -1 -9 5.03860 1.51947 2

9 1 -10 2.09998 1.84241 8

-9 -1 -10 0.40612 1.51920 2

-9 -1 -11-1.36542 1.64614 2

10 -1 -10 13.1567 1.92528 8

-10 1 -10 10.4847 1.80202 2

10 1 10 10.5490 2.24794 6

10 -1 -9 2.02612 1.69039 8

-10 1 -9 0.48758 1.49356 2

10 1 9-2.22877 2.08483 6

-10 1 -8 13.5042 1.66491 2

10 -1 -8 15.3688 1.78347 8

10 1 8 9.47664 2.10210 6

10 -1 -7 3.18926 1.53248 8

-10 1 -7 3.03668 1.36496 2

10 1 7 4.63508 1.88757 6

-10 1 -6-0.32579 1.26916 2

10 1 6 1.43407 1.61261 6

-10 1 -5 5.89719 1.28535 2

10 1 5 5.88493 1.56609 6

10 -1 -4 5.52976 1.35129 7

-10 1 -4 5.20416 1.20752 2

10 1 4 6.01510 1.43783 6

10 -1 -3 26.1886 1.69434 7

10 1 3 28.4911 1.77585 6

-10 1 -3 29.8375 1.58499 2

10 1 2 20.3948 1.53194 6

-10 1 -2 20.0257 1.41188 2

10 -1 -2 20.1741 1.47962 7

10 -1 -1 10.9904 1.28935 7

10 1 1 9.42637 1.22940 6

-10 1 -1 9.41464 1.15094 2

10 -1 0 1.22521 1.05159 7

-10 -1 0 0.01975 1.02627 2

-10 1 0 0.07914 1.04057 2

10 1 0 1.74284 1.08011 6

10 -1 1 9.27343 1.16553 7

-10 -1 -1 8.80977 1.16419 2

10 1 -1 8.28645 1.13092 6

10 -1 2 20.3958 1.41356 7

10 1 -2 19.2473 1.36156 6

-10 -1 -2 19.5118 1.41402 2

10 -1 3 30.2284 1.68950 7

-10 -1 -3 31.5761 1.67290 2

10 1 -4 5.54944 1.30832 8

10 -1 4 5.85487 1.18102 7

-10 -1 -4 5.70100 1.21260 2

10 -1 5 4.65023 1.19929 7

-10 -1 -5 4.08743 1.25704 2

10 1 -5 6.95634 1.42863 8

10 1 -6-0.30632 1.38822 8

-10 -1 -6-0.57918 1.29477 2

10 1 -7 3.37361 1.57082 8

-10 -1 -7 3.53858 1.37837 2

-10 -1 -8 14.9931 1.72331 2

10 1 -8 12.4300 1.78513 8

10 1 -9 3.88451 1.72839 8

-10 -1 -9 0.24506 1.51464 2

10 1 -10 13.6433 2.08721 8

-10 -1 -10 10.3920 1.80027 2

11 -1 -9 4.30977 1.82949 8

-11 1 -9 1.11106 1.60435 2

11 1 9-1.67725 2.18629 6

11 -1 -8 2.21894 1.66877 8

-11 1 -8 3.73822 1.59060 2

11 1 8 1.24785 1.97569 6

11 -1 -7 17.2958 1.87944 8

11 1 7 16.1113 2.16835 6

-11 1 -7 17.0673 1.71618 2

11 1 6 13.1900 1.98011 6

-11 1 -6 11.2107 1.60496 2

-11 1 -5 3.83847 1.36341 2

11 1 5 4.41836 1.65862 6

11 -1 -4 3.83352 1.41258 7

-11 1 -4 6.11330 1.31146 2

11 1 4 6.91003 1.59645 6

11 -1 -3 8.30164 1.46546 7

11 1 3 10.2236 1.52497 6

-11 1 -3 8.97455 1.26342 2

11 -1 -2 41.7769 2.06144 7

11 1 2 39.9665 2.01200 6

-11 1 -2 43.8587 1.96056 2

-11 -1 1 5.14933 1.24043 2

-11 1 -1 4.25864 1.23284 2

11 -1 -1 8.56906 1.34167 7

11 1 1 4.77034 1.26858 6

11 1 0 4.99578 1.19476 6

-11 -1 0 5.45217 1.22101 2

11 -1 0 6.75994 1.29451 7

-11 1 0 4.24964 1.19873 2

-11 1 1 5.12812 1.27009 2

-11 -1 -1 4.36455 1.26415 2

11 -1 1 5.70889 1.19258 7

11 1 -1 7.78795 1.23457 6

11 -1 2 41.7344 1.93612 7

11 -1 2 39.4148 2.07059 6

-11 -1 -2 44.3375 1.97541 2

11 1 -3 9.17180 1.39919 8

-11 -1 -3 9.11345 1.29669 2

11 -1 3 8.63015 1.31288 7

11 -1 4 6.01327 1.32561 7

-11 -1 -4 4.70564 1.28524 2

11 1 -4 5.63428 1.41294 8

-11 -1 -5 6.44237 1.41984 2

11 1 -5 6.61956 1.48773 8

11 1 -6 11.4885 1.70193 8

-11 -1 -6 11.5351 1.61741 2

11 1 -7 17.3456 1.87529 8

-11 -1 -7 15.9030 1.71242 2

11 1 -8 1.95790 1.66047 8

-11 -1 -8 2.37437 1.56637 2

-11 -1 -9 0.31938 1.59971 2

12 -1 -7 0.96826 1.68690 8

-12 1 -7 2.11946 1.52132 2

12 1 7 1.28176 1.98876 6

12 1 6 16.8469 2.13230 6

-12 1 -6 16.2865 1.77106 2

-12 1 -5-0.77034 1.41279 2

12 1 5 0.74558 1.70026 6

12 -1 -4 3.52319 1.52802 7

12 1 4 7.35841 1.70675 6

-12 1 -4 4.44572 1.41878 2

12 -1 -3 0.45842 1.40434 7

12 1 3 2.07154 1.49897 6

-12 1 -3 0.36427 1.26763 2

-12 1 -2 3.06645 1.30773 2

12 -1 -2 2.97183 1.43506 7

12 1 2 2.63475 1.42232 6

12 -1 -1 44.2184 2.07070 7

12 1 1 39.2523 2.01742 6

-12 -1 1 39.3239 2.00299 2

-12 1 -1 43.5441 2.03226 2

-12 -1 0 3.21465 1.29876 2

-12 1 0 3.50007 1.29111 2

12 1 0 3.23021 1.30606 6

12 -1 0 6.02586 1.38826 7

12 -1 1 45.7303 2.08529 7

-12 1 1 41.1046 2.04818 2

-12 -1 -1 45.3114 2.07723 2

12 -1 2 2.08087 1.26679 7

-12 -1 -2 2.99543 1.30208 2

12 1 -3 0.81289 1.38441 8

-12 -1 -3 1.65580 1.30930 2

12 -1 3 1.11858 1.57503 6

12 1 -4 6.39386 1.56198 8

-12 -1 -4 5.60221 1.44777 2

12 -1 4 3.62221 1.73829 6

12 1 -5 0.44937 1.49328 8

-12 -1 -5 1.35636 1.39252 2

12 1 -6 16.9836 1.86064 8

-12 -1 -6 17.4393 1.78661 2

12 1 -7 2.92235 1.73658 8

-12 -1 -7 1.20230 1.49279 2

-13 1 -5-0.55665 1.49419 2

13 1 5 2.46842 1.83607 6

-13 1 -4 3.02593 1.47921 2

13 1 4 4.13269 1.75903 6

-13 1 -3 2.09942 1.37512 2

13 -1 -3 3.43622 1.55686 7

13 1 3 2.67624 1.61810 6

-13 -1 2 0.15307 1.32615 2

-13 1 -2 1.77805 1.35133 2

13 1 2 0.47653 1.47250 6

13 -1 -2 2.52470 1.46827 7

-13 1 -1 1.55293 1.38701 2

13 -1 -1 1.69642 1.42163 7

-13 -1 1 1.63928 1.34105 2

13 1 1 0.88430 1.37936 6

-13 -1 0 2.34753 1.33151 2

-13 1 0 3.49661 1.32484 2

13 -1 0 2.79744 1.40993 7

-13 1 1 1.49464 1.34164 2

13 -1 1 1.43264 1.39878 7

-13 -1 -1 3.50868 1.41698 2

-13 1 2 0.69090 1.32595 2

-13 -1 -2 2.71552 1.40576 2

13 1 -3 4.70110 1.53637 8

-13 -1 -3 3.07195 1.38820 2

13 1 -4 6.04526 1.57186 8

-13 -1 -4 4.41258 1.48776 2

13 -1 4 4.64131 1.84313 6

13 1 -5 5.40795 1.64224 8

-13 -1 -5 0.97104 1.52178 2

13 -1 5 2.34494 1.94482 6

14 -1 -2 15.9386 1.82530 7

14 1 2 15.2030 1.79156 6

-14 -1 2 16.6848 1.73857 2

-14 1 -2 18.3095 1.74664 2

-14 -1 1 11.6238 1.57567 2

-14 1 -1 11.4156 1.66187 2

-14 -1 0-1.75545 1.45683 2

-14 1 0 1.92367 1.46180 2

-14 1 1 8.34388 1.54633 2

-14 -1 -1 9.93119 1.60711 2

-14 1 2 14.2289 1.70241 2

-14 -1 -2 18.4285 1.74675 2

0 -2 0 1129.61 25.2554 3

0 -2 0 1090.55 25.4419 1

0 -2 0 1110.17 25.2632 6

0 -2 0 1093.86 25.2328 7

0 2 0 1134.41 25.5135 1

0 -2 0 1099.76 25.1762 4

0 -2 -1 0.45720 0.46905 4

0 2 -1 0.01482 0.67295 1

0 2 1 1.59592 0.54390 4

0 2 1 1.80396 0.75353 1

0 -2 1-0.18085 0.40691 4

0 2 1 2.70186 0.72633 3

0 -2 -1 0.70505 0.65241 3

0 -2 -1 2.21126 0.52707 6

0 -2 1-0.48256 0.54515 3

0 -2 1 0.27405 0.49996 6

0 -2 -1 0.62217 0.47784 7

0 -2 1 0.48282 0.64110 1

0 -2 -1 1.27057 0.66123 1

0 -2 -1 1.73042 0.70155 5

0 -2 1-0.15615 0.57110 8

0 -2 2 45.0898 1.47044 8

0 -2 -2 40.7676 1.28314 6

0 2 2 47.7602 1.47169 3

0 -2 2 42.5396 1.50738 1

0 -2 -2 42.5846 1.52129 1

0 -2 -2 44.2037 1.41020 3

0 -2 2 45.6267 1.45808 6

0 -2 -2 44.6536 1.26779 7

0 2 -2 40.7665 1.56702 1

0 2 2 38.6544 1.53291 1

0 -2 -2 48.4533 1.53955 5

0 -2 -2 42.8020 1.28508 4

0 2 2 38.6921 1.08854 2

0 2 2 46.4474 1.37285 4

0 -2 -3 0.18960 0.46909 4

0 -2 3 1.91592 0.80424 8

0 2 3 0.15144 0.58764 4

0 -2 -3-0.16951 0.42015 7

0 2 3-0.16116 0.36375 7

0 2 -3 1.52839 0.75794 1

0 2 3-0.09333 0.65996 3

0 2 3-0.50347 0.55769 6

0 2 3-0.49410 0.72335 1

0 -2 3-0.01262 0.64185 1

0 -2 -3-0.55262 0.67544 1

0 -2 -3-0.17269 0.44231 6

0 -2 -3 0.09360 0.61487 5

0 -2 4 32.6589 1.36624 8

0 -2 4 30.1384 1.21231 1

0 -2 -4 30.0939 1.25267 1

0 2 4 32.1492 1.30965 3

0 2 4 32.3162 1.12358 6

0 2 -4 30.9607 1.33682 1

0 2 4 30.2190 1.28694 1

0 -2 -4 29.7838 1.13995 4

0 2 4 31.3559 1.20519 4

0 2 5-0.07824 0.68591 4

0 2 -5 0.31276 0.72684 1

0 2 5-0.04206 0.53659 6

0 2 5 0.18445 0.80516 3

0 2 5-0.63940 0.71203 1

0 2 6 4.22154 0.89465 4

0 2 6 4.48858 0.95526 3

0 2 7 0.41885 0.92628 4

0 2 7 2.50414 1.04591 3

0 2 8 0.02139 1.07212 4

0 2 8 2.10745 1.18736 3

0 2 9-0.44963 1.25977 4

0 2 9 4.03796 1.44476 3

0 2 10 21.8049 2.02314 3

0 2 10 14.8987 1.69552 4

0 2 11-1.45031 1.57077 4

0 2 11 0.78659 2.02030 3

0 2 12 6.07743 1.71355 4

0 2 12-3.45469 2.48604 3

0 2 13 2.23497 1.73482 4

0 2 13-0.35755 2.31520 3

1 2 13 9.59875 2.54762 3

1 2 13 13.4900 1.83408 4

1 2 12-0.28087 1.57488 4

1 2 11 0.74439 1.48506 4

1 2 11-0.07957 2.05199 3

1 2 10 54.2447 2.59175 3

1 2 10 39.0362 2.18827 4

1 2 9 1.18876 1.18978 4

1 2 9 3.96030 1.40774 3

1 2 8 14.0365 1.37144 3

-1 2 -8 10.8954 1.25553 2

1 2 8 12.7506 1.27617 4

-1 2 -7 22.7854 1.40429 2

1 2 7 24.8386 1.44498 3

1 2 7 25.1043 1.36422 4

1 2 6 2.27741 0.81610 4

1 2 6 2.52300 0.95959 3

-1 2 -6 2.21716 0.89253 2

1 2 5 1.85928 0.70969 4

1 2 5 0.85012 0.79614 3

1 2 5 1.78559 0.63668 6

-1 2 -5 1.74477 0.76474 2

-1 2 -5 1.40223 0.71599 1

-1 -2 4 22.7635 1.09951 8

1 -2 -4 21.7392 1.06872 1

-1 2 -4 20.5347 1.04451 2

1 -2 -4 19.6455 0.99035 7

1 2 4 23.6487 1.14917 3

1 2 4 26.3268 1.03075 6

-1 2 -4 24.0190 1.19110 1

-1 -2 4 22.1067 1.02358 1

1 2 4 24.0012 1.01679 4

-1 -2 3 70.4554 2.16426 8

-1 2 -3 66.5204 2.03849 2

1 -2 -3 73.1968 2.08251 7

1 -2 -3 77.2942 2.25387 1

1 2 3 82.0072 2.24508 3

-1 -2 3 76.4140 2.20801 1

1 2 3 86.2214 2.17779 6

-1 2 -3 79.8798 2.37108 1

1 -2 -3 79.4776 2.27622 5

1 -2 -3 76.4984 2.08700 4

1 2 3 78.0462 2.09260 4

-1 2 -2 19.4522 0.74322 2

1 -2 -2 17.4360 1.01980 1

1 -2 -2 21.1508 0.89280 7

1 2 2 24.6712 1.05788 3

1 -2 -2 21.3546 0.95515 3

1 2 2 26.0513 1.00213 6

-1 -2 2 21.7970 0.96610 6

1 -2 -2 20.3092 0.80848 6

-1 -2 2 19.0221 1.01106 1

-1 2 -2 22.1576 1.18962 1

1 -2 -2 23.1726 1.07780 5

1 2 2 19.6896 0.63465 2

1 2 2 23.1163 1.18879 1

1 -2 -2 21.5066 0.89109 4

1 2 2 26.0729 0.92386 4

-1 -2 2 19.0497 0.91276 8

-1 -2 1 0.64642 0.50285 8

1 -2 -1 0.31957 0.59874 1

1 -2 -1 0.57146 0.46351 4

-1 -2 1 1.03434 0.60012 1

-1 -2 1 0.48880 0.32664 4

1 2 1 0.30981 0.43483 4

1 -2 -1 0.25143 0.47774 7

-1 2 -1 0.45433 0.75741 1

1 2 1-0.46384 0.75500 1

1 2 1 0.42718 0.62766 3

-1 -2 1 0.33641 0.48444 3

1 -2 -1 0.27055 0.54178 3

1 2 1 0.22287 0.57255 6

1 -2 -1 0.52337 0.43238 6

-1 -2 1 0.31945 0.51333 6

1 -2 -1 0.47495 0.61641 5

-1 -2 0 0.09086 0.31967 4

1 -2 0 0.18427 0.45994 4

-1 -2 0 0.05286 0.63291 1

1 2 0-0.71276 0.77635 1

-1 2 0-0.29037 0.75465 1

1 -2 0-0.03736 0.48963 7

1 -2 0 0.20984 0.48223 3

-1 -2 0 0.28138 0.49553 3

1 -2 0-0.24091 0.47139 6

-1 -2 0-0.12571 0.44783 6

1 -2 0-0.26293 0.67916 1

-1 -2 -1 0.24297 0.32981 7

-1 -2 -1 0.53506 0.33056 4

-1 -2 -1 0.40060 0.57388 1

1 -2 1 0.12143 0.45955 4

-1 2 1 0.28800 0.56732 4

1 -2 1 0.39739 0.64156 1

1 2 -1 0.11400 0.71770 1

-1 2 1 0.14201 0.70818 1

-1 2 1 0.03220 0.62034 3

1 -2 1 0.27533 0.51967 3

-1 2 1 0.33914 0.21796 2

-1 -2 -1 0.62477 0.51177 3

-1 -2 -1 0.22137 0.42846 6

-1 2 2 15.9507 0.68651 2

-1 -2 -2 18.6092 0.72295 2

1 -2 2 18.9901 1.03453 1

-1 -2 -2 21.5104 0.69469 7

-1 2 2 22.0398 0.80075 7

-1 2 2 22.9436 1.00433 3

-1 -2 -2 23.1628 0.95635 3

-1 -2 -2 21.0557 1.04104 1

-1 -2 -2 21.3006 0.85606 6

-1 2 2 22.8305 1.14417 1

-1 -2 -2 22.8549 1.03400 5

1 2 -2 19.3135 1.12504 1

-1 -2 -2 19.4842 0.76828 4

-1 2 2 22.4045 0.96536 4

1 -2 3 82.4611 2.26640 8

-1 -2 -3 65.6461 2.03617 2

1 -2 3 72.9470 2.22449 1

-1 2 3 78.8589 2.22491 3

-1 -2 -3 83.8070 2.27481 1

-1 -2 -3 74.4079 2.06704 6

-1 2 3 81.6124 2.34171 1

-1 -2 -3 80.4816 2.27970 5

-1 2 3 78.8080 2.02192 7

-1 -2 -3 67.0581 2.05517 4

-1 2 3 83.3179 2.22872 4

1 -2 4 22.2235 1.07259 1

-1 -2 -4 23.0912 1.09142 2

-1 2 4 23.7424 1.16566 3

-1 -2 -4 24.0679 1.09151 1

-1 2 4 22.6088 1.11559 1

-1 -2 -4 23.9359 1.14584 5

-1 2 4 23.8897 1.11528 4

-1 2 5 3.50941 0.82267 4

-1 2 5 3.64604 0.88792 3

-1 2 5 4.27833 0.71358 1

-1 -2 -5 1.00076 0.80116 2

-1 2 6 2.70697 0.87953 4

-1 2 6 1.54687 0.66456 1

-1 2 6 2.18595 0.92774 3

-1 -2 -6 2.28642 0.91567 2

-1 2 7 26.0463 1.44675 3

-1 2 7 22.1953 1.38667 4

-1 2 8 12.1664 1.33586 3

-1 2 8 11.6105 1.30966 4

-1 2 9 0.05059 1.24931 4

-1 2 9 2.41229 1.39305 3

-1 2 10 53.7857 2.54031 3

-1 2 10 39.1886 2.24673 4

-1 2 11-0.30527 1.95584 3

-1 2 11 0.12710 1.59619 4

-1 2 12-3.64290 2.36917 3

-1 2 12 2.04747 1.68556 4

-1 2 13 8.67132 2.34960 3

-1 2 13 14.8982 1.99847 4

2 2 13 4.80581 1.64931 4

2 2 13 1.58725 2.37555 3

2 2 12 2.61916 1.54041 4

2 2 11 0.71344 1.44303 4

2 2 11 1.12135 2.00539 3

2 2 10 1.26753 1.37375 4

-2 2 -10 1.30812 1.30369 2

2 2 9 3.32276 1.24251 4

2 2 9 5.09767 1.46629 3

-2 2 -9 5.08393 1.24414 2

2 2 8 2.57432 1.08110 4

2 2 8 6.46308 1.25372 3

-2 2 -8 3.38964 1.16873 2

-2 2 -7 79.9696 2.57453 2

2 2 7 85.4901 2.61041 3

2 2 7 72.1346 2.47964 4

-2 2 -6 46.6247 1.79548 2

2 2 6 51.9477 1.88211 3

2 2 6 46.9949 1.64146 6

2 2 6 48.7705 1.73019 4

2 -2 -5 16.5059 0.97797 7

-2 2 -5 14.9794 0.99714 2

2 2 5 14.7630 1.07409 3

2 2 5 15.1594 0.95079 6

-2 2 -5 16.1330 1.06986 1

2 2 5 15.4236 0.92971 4

2 -2 -4 118.561 3.21088 1

-2 -2 4 108.395 3.10168 8

-2 2 -4 117.860 3.08270 2

2 -2 -4 108.426 3.13418 7

2 2 4 123.544 3.20508 3

2 2 4 125.997 3.15589 6

-2 2 -4 131.999 3.36410 1

-2 -2 4 122.056 3.14084 1

2 2 4 112.897 3.03113 4

2 -2 -3 52.3200 1.79127 1

-2 -2 3 64.0482 1.72234 8

-2 2 -3 49.6664 1.54881 2

2 -2 -3 50.4795 1.66714 7

2 2 3 52.2153 1.71374 3

-2 2 -3 56.3924 1.94170 1

2 2 3 52.8603 1.69970 6

-2 -2 3 67.8646 1.83248 1

2 -2 -3 55.2627 1.64916 4

2 2 3 47.1043 1.51957 4

-2 2 -2 11.0001 0.57240 2

-2 -2 2 9.95261 0.65798 8

2 -2 -2 9.13188 0.84250 1

2 2 2 10.7722 0.79198 3

-2 2 -2 11.8854 1.06544 1

2 -2 -2 8.30835 0.71242 3

2 -2 -2 7.99390 0.55892 6

2 2 2 10.5104 0.80045 6

-2 -2 2 9.25437 0.79947 1

2 -2 -2 9.85941 0.81646 5

2 -2 -2 9.65490 0.68977 4

2 2 2 9.97672 0.58164 4

2 -2 -2 8.97534 0.72579 7

2 -2 -1 5.29670 0.73488 1

2 -2 -1 5.52705 0.61655 7

-2 -2 1 4.74077 0.57179 3

2 -2 -1 5.78996 0.54264 6

2 2 1 5.23956 0.70417 3

-2 -2 1 4.72138 0.60863 6

2 -2 -1 5.72777 0.62563 3

2 2 1 5.29662 0.68729 6

-2 -2 1 4.91928 0.72987 1

2 -2 -1 5.93667 0.72364 5

-2 2 -1 4.67530 0.92334 1

2 -2 -1 5.75698 0.60346 4

-2 2 -1 5.38212 0.37812 2

2 2 1 4.89979 0.44490 4

2 -2 0 4.69823 0.75124 1

2 -2 0 5.34728 0.63287 7

2 -2 0 5.13436 0.59719 3

-2 -2 0 4.45459 0.58255 3

2 2 0 5.72952 0.70288 6

-2 -2 0 4.78000 0.57824 6

2 -2 0 6.19892 0.67976 5

-2 -2 0 4.23579 0.72820 1

-2 2 0 5.05715 0.88483 1

-2 2 0 4.88314 0.31675 2

2 -2 0 5.36072 0.57542 4

-2 2 1 6.84858 0.70928 4

2 -2 1 4.74456 0.75353 1

-2 -2 -1 4.21400 0.36813 7

2 -2 1 6.24136 0.66687 7

2 -2 1 4.96117 0.59284 3

-2 -2 -1 4.78508 0.60185 3

2 2 -1 6.76897 0.73385 6

-2 2 1 5.63058 0.72365 3

-2 -2 -1 5.46807 0.54886 6

-2 -2 -1 4.95575 0.70997 1

-2 2 1 6.00434 0.88458 1

-2 -2 -1 5.60457 0.37037 2

2 -2 1 5.73797 0.58327 4

-2 -2 -2 11.0778 0.57317 2

2 -2 2 9.98422 0.88305 1

-2 2 2 9.31480 0.62801 7

-2 2 2 10.1494 0.79631 3

-2 -2 -2 9.62362 0.71555 3

-2 2 2 10.7683 0.96171 1

-2 -2 -2 9.76316 0.65947 6

-2 -2 -2 9.65542 0.84877 1

-2 -2 -2 9.61646 0.81070 5

-2 2 2 10.7983 0.81653 4

2 2 -3 62.6801 2.06193 8

2 -2 3 51.4266 1.73599 1

-2 2 3 56.9548 1.72498 3

-2 -2 -3 51.7795 1.55488 2

-2 2 3 52.9555 1.55309 7

-2 2 3 57.3604 1.87596 1

-2 -2 -3 45.8586 1.56694 6

-2 -2 -3 51.8221 1.75269 1

-2 -2 -3 51.2094 1.74686 5

-2 2 3 53.3656 1.74936 4

2 2 -4 118.085 3.53263 8

-2 -2 -4 123.634 3.17338 2

-2 2 4 117.211 3.06791 7

-2 2 4 119.760 3.25596 3

-2 2 4 124.651 3.32179 1

-2 -2 -4 125.308 3.30277 1

-2 -2 -4 120.910 3.28455 5

-2 2 4 119.762 3.29568 4

-2 -2 -5 14.0974 1.00357 2

-2 2 5 15.3740 1.06420 3

-2 2 5 16.2088 0.99175 1

-2 2 5 15.4768 1.05683 4

-2 -2 -6 46.2517 1.77302 2

-2 2 6 50.5227 1.85886 3

-2 2 6 44.6812 1.59903 1

-2 2 6 48.1373 1.80115 4

-2 2 7 83.9278 2.60808 3

-2 2 7 77.3435 2.59763 4

-2 2 8 3.33238 1.15587 4

-2 2 8 3.19000 1.16070 3

-2 2 9 4.30167 1.33671 4

-2 2 9 6.70384 1.43375 3

-2 2 10 2.10489 1.45656 4

-2 2 10 4.80344 1.63881 3

-2 2 11 0.32438 1.58215 4

-2 2 11-0.64255 1.83879 3

-2 2 12 0.87586 1.73485 4

-2 2 12-4.34412 2.17945 3

-2 2 13 4.11976 1.87914 4

-2 2 13 0.53087 2.13529 3

3 2 13 1.61491 1.53726 4

3 2 13-0.40813 2.44070 3

3 2 12-2.90646 2.37258 3

3 2 12 4.65553 1.53314 4

3 2 11 6.46424 1.48485 4

3 2 11 4.55972 2.02944 3

3 2 10 5.20312 1.48127 4

3 2 10 11.1674 1.79647 3

-3 2 -10 6.22787 1.38723 2

-3 2 -9 39.1948 1.92826 2

3 2 9 47.6833 2.17563 3

3 2 9 33.5482 1.95862 4

3 2 8 0.24687 1.11272 4

3 2 8 3.54737 1.17702 3

-3 2 -8 0.83023 1.04776 2

3 2 7 11.1039 1.18639 3

-3 2 -7 7.30246 1.06544 2

3 2 7 8.34512 1.09106 4

-3 2 -6 22.0115 1.25373 2

3 2 6 24.2983 1.34247 3

3 2 6 22.0517 1.21161 6

3 2 6 23.6932 1.21742 4

-3 2 -5 28.7946 1.26081 2

3 -2 -5 30.9937 1.36869 7

3 2 5 30.2082 1.35043 3

-3 2 -5 32.2070 1.44604 1

3 2 5 31.0432 1.31182 6

3 2 5 29.4087 1.19795 4

-3 2 -4 13.1390 0.88210 2

3 -2 -4 16.1574 0.97908 7

3 2 4 15.1673 1.00012 3

-3 2 -4 15.8076 1.19996 1

3 2 4 16.1614 1.00274 6

3 2 4 14.1939 0.77915 4

-3 2 -3 28.1315 1.05997 2

3 -2 -3 29.2487 1.17102 7

-3 2 -3 29.6170 1.50594 1

3 2 3 28.2454 1.19130 3

3 2 3 28.6668 1.22893 6

3 2 3 24.6906 0.93685 4

3 -2 -2 48.5212 1.62307 7

-3 -2 2 46.6722 1.53655 3

-3 2 -2 51.8699 1.98627 1

3 2 2 65.6970 1.72758 3

3 -2 -2 46.7329 1.56226 3

3 2 2 58.9753 1.75457 6

3 2 2 51.2363 1.40372 4

-3 2 -2 47.0614 1.43428 2

3 -2 -1 45.9977 1.44894 7

3 2 1 41.6410 1.42059 3

-3 2 -1 35.9912 1.73877 1

-3 -2 1 42.9465 1.32071 3

3 -2 -1 46.4579 1.37760 3

3 2 1 42.5231 1.48173 6

3 -2 -1 47.3898 1.51686 5

-3 2 -1 39.7451 1.16591 2

3 -2 -1 46.7483 1.42542 4

3 2 1 35.7041 1.07993 4

3 2 0 0.00802 0.62523 8

3 -2 0-0.34569 0.54471 4

-3 -2 0 0.16498 0.35422 2

-3 2 0-0.23506 0.35259 2

3 -2 0 0.50645 0.62275 7

3 -2 0-0.21390 0.54316 3

-3 -2 0-0.06868 0.55489 3

-3 2 0 0.93829 0.93256 1

3 2 0 0.55632 0.67362 6

3 -2 0 0.01209 0.61552 5

3 2 -1 39.9534 1.45851 8

3 -2 1 42.3596 1.48131 7

-3 2 1 40.3119 1.79581 1

-3 2 1 46.5295 1.37555 7

3 -2 1 41.6673 1.31666 3

-3 -2 -1 39.7703 1.34076 3

3 2 -1 44.6300 1.49861 6

-3 -2 -1 39.8746 1.35007 6

3 -2 1 41.5613 1.42689 5

-3 -2 -1 38.8282 1.16286 2

3 -2 1 40.5413 1.38449 4

3 2 -2 43.0151 1.74887 8

3 -2 2 43.9247 1.50145 3

-3 2 2 48.8620 1.52632 7

3 -2 2 49.1595 1.69213 7

-3 2 2 50.2363 1.61881 3

-3 2 2 47.9980 1.91983 1

-3 -2 -2 57.5044 1.65446 3

3 2 -2 45.1724 1.68645 6

-3 -2 -2 53.4282 1.62739 6

-3 -2 -2 53.9853 1.45413 2

-3 2 2 48.5319 1.70647 4

-3 -2 -3 26.7605 1.03586 2

3 2 -3 25.3833 1.43426 8

-3 2 3 27.8239 1.05390 7

-3 2 3 28.5178 1.21001 3

-3 2 3 28.9091 1.43939 1

-3 -2 -3 25.4726 1.18260 5

-3 2 3 28.5141 1.27206 4

3 2 -4 15.4618 1.37769 8

-3 -2 -4 14.4269 0.90170 2

-3 2 4 14.6155 1.00616 3

-3 2 4 15.4166 1.10511 1

-3 -2 -4 13.8726 0.96294 5

-3 2 4 16.3683 1.07413 4

3 2 -5 30.1213 1.70907 8

-3 -2 -5 29.1106 1.28599 2

-3 2 5 29.5133 1.34825 3

-3 2 5 29.8508 1.33509 1

-3 2 5 30.0414 1.39831 4

3 2 -6 24.2337 1.71404 8

-3 -2 -6 21.8581 1.24853 2

-3 2 6 23.1466 1.32199 3

-3 2 6 23.3944 1.15958 1

-3 2 6 22.9753 1.35019 4

3 2 -7 6.44505 1.49398 8

-3 2 7 9.58689 1.14113 3

-3 2 7 9.27547 1.15897 4

3 2 -8 0.59330 1.50672 8

-3 2 8 1.29059 1.15151 4

-3 2 8 4.64324 1.21900 3

-3 2 9 44.1225 2.08655 3

-3 2 9 38.9812 2.03558 4

-3 2 10 6.86912 1.55151 4

-3 2 10 9.03101 1.63637 3

-3 2 11 5.84100 1.74263 4

-3 2 11 4.94044 1.82884 3

-3 2 12 3.25127 1.76634 4

-3 2 12-0.46108 2.05474 3

-3 2 13 2.42430 1.88941 4

-3 2 13-0.27471 2.07665 3

4 2 13 3.03253 2.39897 3

4 2 12 6.20448 2.27571 3

4 2 11 3.26996 1.91106 3

4 2 10 3.69411 1.65025 3

4 2 9 3.44876 1.41574 3

-4 2 -9 0.15861 1.18341 2

-4 2 -8 52.9117 2.18054 2

4 2 8 68.1303 2.37227 3

4 2 7-0.01877 1.07641 3

4 2 7 1.05297 0.99002 6

-4 2 -7-1.19827 0.95707 2

4 2 6 1.02373 0.95269 3

4 2 6 0.59540 0.96043 6

-4 2 -6-0.62125 0.88814 2

4 -2 -5 1.99939 0.90051 7

4 2 5 3.38973 0.86954 3

-4 2 -5 1.04193 0.76874 2

-4 2 -5 2.35262 1.00351 1

4 2 5 3.21365 0.91860 6

4 -2 -4 2.68226 0.83051 7

-4 2 -4 3.28724 1.01374 1

-4 2 -4 2.55651 0.69946 2

4 2 4 2.91379 0.80264 3

4 2 4 2.11205 0.81316 6

4 -2 -3 68.8876 2.16364 7

-4 2 -3 72.4813 2.48088 1

4 2 3 74.3401 2.08027 3

4 2 3 68.5946 2.17284 6

-4 2 -3 66.6600 1.96863 2

4 -2 -2 39.5381 1.43968 7

-4 2 -2 37.7216 1.79398 1

-4 -2 2 36.3159 1.27439 3

4 2 2 40.9730 1.39297 3

4 -2 -2 36.6276 1.31322 3

4 2 2 42.7117 1.52654 6

-4 2 -2 38.4948 1.24909 2

-4 2 -1 46.0102 2.18103 1

-4 -2 1 47.7097 1.60947 3

4 -2 -1 74.4240 1.79206 3

4 2 1 55.5812 1.84826 6

4 -2 -1 70.3175 1.90257 7

-4 2 -1 51.4787 1.56590 2

-4 -2 0 0.92472 0.47926 2

-4 2 0-0.23104 0.44780 2

-4 2 0 0.31424 0.98863 1

4 -2 0-0.23590 0.69946 7

4 -2 0-0.33005 0.56504 3

-4 -2 0-0.05161 0.58479 3

4 2 0 1.75093 0.77360 6

4 -2 0 0.42273 0.62992 5

4 2 -1 53.7178 1.67667 8

-4 2 1 67.0785 2.35756 1

4 -2 1 54.0711 1.79487 7

4 -2 1 51.8179 1.58513 3

-4 -2 -1 53.4214 1.63714 3

4 2 -1 58.2684 1.85810 6

4 -2 1 54.6198 1.72645 5

-4 -2 -1 51.1113 1.52237 2

4 2 -2 33.1402 1.43224 8

-4 2 2 38.8193 1.81838 1

4 -2 2 37.5807 1.49265 7

4 -2 2 34.1459 1.25507 3

-4 2 2 39.1308 1.37184 3

-4 -2 -2 38.9263 1.33260 3

4 2 -2 37.4004 1.49190 6

4 -2 2 38.9102 1.34741 5

-4 -2 -2 39.2097 1.23761 2

-4 2 2 39.8200 1.54852 4

4 2 -3 61.2799 2.16889 8

-4 2 3 67.1304 2.37956 1

4 -2 3 69.9438 2.18571 7

-4 2 3 66.0960 1.99445 3

4 2 -3 64.9385 2.14961 6

-4 -2 -3 66.8115 2.03535 5

-4 -2 -3 66.2182 1.91249 2

-4 2 3 68.2637 2.16955 4

4 2 -4 2.53353 1.07072 8

-4 2 4 2.86832 0.85149 4

4 -2 4 3.07597 0.91477 7

-4 -2 -4 2.73978 0.71225 2

-4 2 4 3.09618 0.79519 3

-4 2 4 2.89118 0.97658 1

-4 -2 -4 2.81236 0.76741 5

4 2 -5 1.41099 1.13003 8

-4 2 5 4.40308 0.96564 4

-4 2 5 2.76163 0.89783 3

-4 -2 -5 2.62122 0.82670 2

-4 2 5 2.52353 0.88917 1

-4 -2 -5 2.85735 0.75937 5

4 2 -6 1.20443 1.35934 8

-4 2 6 1.41878 1.00395 4

-4 2 6 1.17479 0.95041 3

-4 2 6 1.96431 0.84759 1

4 2 -7-1.02474 1.42376 8

-4 2 7-0.35646 1.03052 4

-4 2 7-0.19867 1.03637 3

4 2 -8 58.6501 2.66778 8

-4 2 8 64.0151 2.31765 3

-4 2 8 64.0155 2.42386 4

4 2 -9-0.55192 1.60548 8

-4 2 9 1.05621 1.28565 4

-4 2 9 3.12494 1.28783 3

4 2 -10-0.22928 1.69030 8

-4 2 10 2.07553 1.47742 4

-4 2 10 3.18948 1.49356 3

-4 2 11 1.09506 1.68276 4

-4 2 11 4.70409 1.77292 3

-4 2 12 14.6037 2.02765 4

-4 2 12 8.28873 2.02530 3

-4 2 13 0.62929 2.01254 3

5 2 9 8.13958 1.45608 3

-5 2 -9 4.37153 1.28405 2

-5 2 -8 12.0074 1.29894 2

5 2 8 15.3262 1.48848 3

-5 2 -7 9.50858 1.16128 2

5 2 7 12.4875 1.27654 3

5 2 7 10.2788 1.30664 6

5 -2 -6 3.26413 1.08201 7

5 2 6 0.57652 0.90648 3

-5 2 -6-0.13455 0.92619 2

5 2 6 2.13960 1.05846 6

-5 2 -5 117.706 3.29004 2

5 -2 -5 119.149 3.52191 7

-5 2 -5 120.234 3.55505 1

5 2 5 119.978 3.28564 3

5 2 5 122.430 3.45243 6

5 -2 -4 20.3168 1.25006 7

-5 2 -4 22.6696 1.46414 1

5 2 4 21.6179 1.12032 3

5 2 4 21.4508 1.28657 6

-5 2 -4 22.6968 1.10096 2

-5 2 -3 25.1597 1.55362 1

5 -2 -3 26.8885 1.31152 7

5 2 3 27.6379 1.16387 3

5 2 3 23.7029 1.28586 6

-5 2 -3 24.7715 1.10205 2

-5 2 -2 41.3649 1.97598 1

5 -2 -2 43.7957 1.61385 7

5 2 2 44.2037 1.46557 3

5 2 2 42.7391 1.63522 6

-5 2 -2 40.4081 1.37709 2

-5 2 -1 5.35683 1.22165 1

5 -2 -1 5.70353 0.85526 7

5 2 1 7.61067 0.94056 6

-5 2 -1 5.71546 0.66012 2

-5 2 0-0.10940 1.14515 1

-5 -2 0-0.55344 0.56296 2

-5 2 0-0.48888 0.56302 2

5 -2 0-0.03073 0.73136 7

5 2 0-0.31944 0.77558 6

5 2 -1 6.26724 0.72083 8

-5 2 1 4.69873 1.23141 1

5 -2 1 6.58616 0.88113 7

5 2 -1 6.88281 0.90749 6

5 -2 1 5.41595 0.75977 5

-5 -2 -1 6.15200 0.69328 2

-5 2 2 43.4169 1.66510 4

5 2 -2 40.7406 1.48808 8

-5 2 2 40.8598 1.99940 1

5 -2 2 40.7307 1.64955 7

-5 2 2 40.9553 1.43918 3

5 2 -2 40.0675 1.63303 6

5 -2 2 43.5521 1.43723 5

-5 -2 -2 41.1556 1.37372 2

5 2 -3 23.8462 1.26507 8

-5 2 3 25.3114 1.58461 1

5 -2 3 26.7698 1.34421 7

-5 2 3 26.1886 1.13873 3

5 2 -3 23.0944 1.32893 6

-5 -2 -3 24.8893 1.18183 5

-5 -2 -3 27.6518 1.13148 2

-5 2 3 25.3211 1.36553 4

5 2 -4 19.6933 1.32893 8

-5 2 4 21.7301 1.43954 1

5 -2 4 20.7588 1.29753 7

-5 2 4 22.2695 1.12038 3

5 2 -4 20.2838 1.28041 6

-5 2 4 19.5144 1.26009 4

-5 -2 -4 21.4064 1.09295 2

-5 -2 -5 115.466 3.22602 2

5 2 -5 107.798 3.44165 8

5 -2 5 122.224 3.49934 7

-5 2 5 116.393 3.18960 3

-5 2 5 113.709 3.39737 1

-5 2 5 119.634 3.37503 4

5 2 -6 0.94042 1.26156 8

-5 2 6 2.88318 0.95435 1

-5 2 6 2.32123 0.98969 3

-5 2 6 3.44028 1.08409 4

5 2 -7 10.1039 1.58461 8

-5 2 7 12.2880 1.21723 3

-5 2 7 14.3614 1.32747 4

5 2 -8 11.2114 1.76662 8

-5 2 8 12.7365 1.39237 3

-5 2 8 15.1718 1.47266 4

5 2 -9 3.04982 1.70625 8

-5 2 9 5.11856 1.42936 4

-5 2 9 8.89540 1.37528 3

5 2 -10 0.67442 1.74033 8

-5 2 10 2.60821 1.55919 4

-5 2 10 4.61332 1.46615 3

5 2 -11 3.00089 1.94638 8

-5 2 11 3.65115 1.67720 4

-5 2 11 1.45497 1.64606 3

-5 2 12 7.88876 1.93773 4

6 2 8 3.30122 1.48491 6

-6 2 -8 2.99004 1.18866 2

-6 2 -7 1.30995 1.07010 2

6 2 7 1.04922 1.25873 6

-6 2 -6 13.8868 1.21474 2

6 -2 -6 17.0963 1.45473 7

6 2 6 17.2966 1.44086 6

6 -2 -5 43.3466 1.91322 7

-6 2 -5 40.9304 1.92949 1

6 2 5 43.5713 1.85204 6

-6 2 -5 42.4462 1.67179 2

-6 2 -4 11.4529 1.34957 1

6 2 4 12.3660 1.20414 6

-6 2 -3 1.16331 1.15475 1

6 -2 -3 1.71405 0.91721 7

-6 2 -3 0.23927 0.71699 2

6 2 3 1.40749 0.91772 6

-6 2 -2 30.6520 1.85558 1

6 -2 -2 34.5126 1.49691 7

6 2 2 32.3704 1.48585 6

-6 2 -2 32.2854 1.25581 2

-6 2 -1 29.4831 1.86830 1

6 -2 -1 33.4375 1.49732 7

6 2 1 31.2138 1.47815 6

-6 2 -1 30.9366 1.22398 2

-6 2 0 0.78720 1.27955 1

-6 -2 0 0.62562 0.68150 2

-6 2 0-0.63544 0.66916 2

6 -2 0 0.33511 0.80797 7

6 2 0 2.49561 0.91381 6

6 2 -1 29.6838 1.24163 8

-6 2 1 28.7475 1.90607 1

6 -2 1 30.2612 1.42377 7

6 2 -1 32.0576 1.48892 6

-6 -2 -1 30.2807 1.20460 2

6 2 -2 32.0458 1.34150 8

-6 2 2 34.2743 1.94352 1

6 -2 2 30.8159 1.51187 7

6 2 -2 38.3989 1.59582 6

-6 -2 -2 34.1947 1.28851 2

6 2 -3 0.49130 0.85911 8

-6 2 3 0.42923 1.16564 1

-6 2 3-0.49880 0.95469 4

-6 -2 -3 0.60881 0.73475 2

6 -2 3 0.60512 0.91871 7

-6 2 3 1.08897 0.73936 3

6 2 -3 0.64081 0.91670 6

6 2 -4 19.3075 1.39717 8

6 -2 4 9.03172 1.25149 7

6 2 -4 15.9593 1.38757 6

-6 -2 -4 9.76709 1.10125 2

6 2 -5 40.5921 1.84888 8

-6 2 5 40.5208 1.87608 1

6 -2 5 42.1386 1.89989 7

-6 2 5 42.5448 1.81647 4

6 2 -6 17.1710 1.54004 8

-6 2 6 15.8504 1.29785 1

6 -2 6 18.4276 1.48575 7

-6 2 6 17.9758 1.35322 4

6 2 -7 1.65274 1.42045 8

-6 2 7 1.92717 1.18555 4

6 -2 7 1.71190 1.25968 7

6 2 -8 3.89291 1.62320 8

-6 2 8 5.75051 1.35871 4

6 2 -9 5.59889 1.88191 8

-6 2 9 8.61649 1.54008 4

6 2 -10-3.13504 1.91068 8

-6 2 10 0.68169 1.55928 4

6 2 -11 0.55473 1.96511 8

-6 2 11 2.42921 1.75676 4

7 2 8 4.34499 1.67275 6

-7 2 -7 2.32717 1.11266 2

7 2 7 3.45680 1.45208 6

7 -2 -6 7.47254 1.35781 7

-7 2 -6 6.60684 1.15654 2

7 2 6 7.18989 1.37865 6

-7 2 -5 2.23850 1.16702 1

7 -2 -5 1.46427 1.12935 7

-7 2 -5 0.97522 0.91895 2

7 2 5 0.96509 1.14536 6

-7 2 -4 2.83239 1.24695 1

7 -2 -4 2.65584 1.06603 7

-7 2 -4 2.72751 0.86784 2

7 2 4 2.05133 1.10094 6

-7 2 -3 5.03004 1.32275 1

7 -2 -3 5.00593 1.06069 7

-7 2 -3 4.56502 0.88556 2

7 2 3 5.52379 1.10270 6

-7 2 -2 46.1919 2.28237 1

7 -2 -2 51.5921 1.94141 7

7 2 2 47.7042 1.90621 6

-7 2 -2 48.5584 1.68925 2

-7 2 -1 147.715 4.98627 1

7 -2 -1 177.603 4.57790 7

7 2 1 167.319 4.53703 6

-7 2 -1 171.544 4.33044 2

-7 2 0 4.43386 1.55662 1

-7 2 0 0.14110 0.84031 2

7 -2 0 3.91710 1.02974 7

7 2 0 16.6908 1.26423 6

-7 -2 0 12.8609 1.04906 2

-7 2 1 147.982 5.15906 1

7 -2 1 171.901 4.60384 7

7 2 -1 176.232 4.64714 6

-7 -2 -1 178.073 4.41070 2

7 2 -2 45.2826 1.71880 8

-7 2 2 51.6558 2.45080 1

7 -2 2 44.5648 1.87515 7

7 2 -2 51.1203 1.97100 6

-7 2 3 4.88993 1.43467 1

7 2 -3 4.12551 0.98291 8

7 -2 3 4.88108 1.05817 7

7 2 -3 4.51423 1.06424 6

7 2 -4 3.62388 1.06160 8

-7 2 4 4.29378 1.30628 1

-7 2 4 2.53769 1.09371 4

7 -2 4 3.10179 1.09638 7

7 2 -4 2.83786 1.07207 6

7 2 -5 3.25574 1.13820 8

-7 2 5 0.39523 1.12144 1

-7 2 5-0.04431 1.08091 4

7 -2 5 0.89838 1.11406 7

7 2 -5 0.69393 1.04837 6

7 2 -6 8.47794 1.39965 8

-7 2 6 7.88619 1.24997 4

7 -2 6 7.53493 1.31578 7

7 2 -7 3.44569 1.43839 8

-7 2 7 1.74593 1.21298 4

7 -2 7 1.67403 1.27992 7

7 2 -8 6.25540 1.65962 8

-7 2 8 5.44216 1.36234 4

7 -2 8 5.65217 1.44985 7

7 2 -9 0.36405 1.75021 8

-7 2 9 1.17387 1.42429 4

7 -2 9 0.77006 1.49998 7

7 2 -10-0.10890 1.88765 8

-7 2 10 0.95278 1.58449 4

7 -2 10-0.93935 1.63518 7

7 2 -11 7.02050 2.10674 8

-7 2 11 9.68202 1.86009 4

7 -2 11 7.99725 1.80639 7

8 2 8-0.31084 1.73446 6

8 2 7-0.81798 1.52252 6

8 -2 -6 7.66907 1.44740 7

-8 2 -6 7.55026 1.20799 2

8 2 6 6.18087 1.45854 6

-8 2 -5 1.25194 1.20239 1

8 -2 -5 1.32091 1.23994 7

-8 2 -5-0.19078 1.03122 2

8 2 5 0.74459 1.25421 6

-8 2 -4 6.38944 1.36820 1

8 -2 -4 6.34758 1.26139 7

-8 2 -4 5.88068 1.03726 2

8 2 4 6.07193 1.29350 6

-8 2 -3 2.07577 1.40418 1

8 -2 -3 2.39973 1.09897 7

-8 2 -3 1.44160 0.89727 2

8 2 3 2.48124 1.17388 6

-8 2 -2 7.05893 1.54984 1

8 -2 -2 9.09026 1.16651 7

8 2 2 7.18154 1.15964 6

-8 2 -2 9.14904 1.00246 2

-8 2 -1 2.27592 0.82820 2

8 -2 -1 3.33243 1.06232 7

8 2 1 4.70182 1.11881 6

-8 2 -1 2.30969 1.61020 1

8 -2 0 19.7181 1.37829 7

8 2 0 19.2145 1.37603 6

-8 2 0 19.9826 1.16225 2

-8 2 0 11.9790 1.91393 1

8 -2 1 2.91889 1.01338 7

8 2 -1 2.90784 1.10238 6

-8 2 1 1.26945 1.74932 1

-8 2 2 7.40796 1.70809 1

8 -2 2 7.92969 1.14222 7

8 2 -2 8.75955 1.20861 6

-8 2 3 2.99044 1.46980 1

8 2 -3 1.81770 1.01615 8

8 -2 3 1.12606 1.07378 7

8 2 -3 0.91046 1.10198 6

-8 2 4 6.04513 1.43085 1

8 2 -4 6.15089 1.16987 8

-8 2 4 7.45518 1.24363 4

8 -2 4 6.42437 1.20157 7

8 2 -4 6.94991 1.20647 6

8 2 -5 1.13976 1.21907 8

-8 2 5 0.78992 1.23202 1

-8 2 5 0.28199 1.18559 4

8 -2 5 0.65657 1.12352 7

8 2 -6 9.19676 1.42919 8

-8 2 6 8.51117 1.36627 4

8 -2 6 8.36757 1.38747 7

8 2 -7 1.00819 1.42185 8

-8 2 7 0.22674 1.25900 4

8 -2 7 0.02834 1.29575 7

8 2 -8 3.01343 1.70300 8

-8 2 8-0.26830 1.33339 4

8 -2 8 0.57583 1.42903 7

8 2 -9 33.2383 2.41580 8

8 -2 9 30.0342 2.18974 7

-8 2 9 37.4045 2.11733 4

8 2 -10 2.40828 2.00478 8

-8 2 10-0.03125 1.53359 4

8 -2 10-0.20495 1.58837 7

8 -2 11 13.3827 1.95881 7

9 2 8 16.2163 2.18951 6

9 2 7 2.61176 1.70451 6

9 -2 -6 16.2492 1.72703 7

9 2 6 18.1253 1.84467 6

-9 2 -5 9.19803 1.47034 1

9 -2 -5 10.9539 1.49204 7

-9 2 -5 8.94981 1.29446 2

9 2 5 10.6617 1.52063 6

-9 2 -4 36.9413 2.15300 1

9 -2 -4 38.6727 2.01913 7

9 2 4 38.8818 2.03551 6

-9 2 -4 39.1025 1.80788 2

-9 2 -3 24.9745 1.96130 1

9 -2 -3 25.9824 1.68659 7

9 2 3 22.0275 1.63832 6

-9 2 -3 21.5907 1.36883 2

-9 2 -2 13.7450 1.86145 1

9 -2 -2 17.3701 1.42412 7

9 2 2 10.8742 1.33826 6

-9 2 -2 11.7272 1.17920 2

9 -2 -1 12.5091 1.30152 7

9 2 1 10.3612 1.29154 6

-9 2 -1 9.46737 1.10687 2

-9 2 -1 10.7084 1.88244 1

-9 2 0 0.06524 0.91614 2

9 -2 0 0.67929 1.14496 7

9 2 0 1.39053 1.13917 6

-9 2 0 0.91403 1.81885 1

9 -2 1 10.8941 1.33114 7

9 2 -1 11.1149 1.31205 6

-9 2 1 8.84797 1.10830 2

-9 2 1 10.9141 2.13928 1

-9 2 2 14.9273 2.04275 1

9 -2 2 10.5190 1.28528 7

9 2 -2 13.6209 1.39487 6

-9 2 3 23.6338 2.06083 1

9 -2 3 23.2343 1.56954 7

9 2 -3 24.2079 1.60130 6

-9 2 4 39.8024 2.25502 1

9 -2 4 36.7780 1.91641 7

9 2 -4 38.8131 1.90294 6

-9 2 5 12.1970 1.40460 4

9 2 -5 11.4033 1.41293 8

-9 2 5 9.60749 1.52714 1

9 -2 5 11.6769 1.42418 7

9 2 -6 16.3861 1.59274 8

9 -2 6 17.3561 1.60369 7

-9 2 6 16.0881 1.61165 4

9 2 -7 4.47635 1.59622 8

-9 2 7 1.96245 1.39829 4

9 -2 7 2.76025 1.39765 7

9 2 -8 18.2490 1.96994 8

9 -2 8 17.2034 1.84655 7

-9 2 8 20.2493 1.79571 4

9 2 -9 2.07154 1.83733 8

-9 2 9 0.53307 1.47550 4

9 -2 9 2.29660 1.54324 7

9 2 -10 2.58589 2.03716 8

10 2 9-0.66341 2.16912 6

10 2 8 2.63115 1.98193 6

10 2 7 20.2477 2.15423 6

10 -2 -6 6.99234 1.64157 7

10 2 6 8.73019 1.79896 6

-10 2 -5 6.98453 1.52994 1

10 -2 -5 5.76858 1.51106 7

10 2 5 8.30390 1.61050 6

-10 2 -4-0.99577 1.53399 1

10 -2 -4 0.11375 1.40227 7

10 2 4 0.74862 1.39830 6

10 -2 -3 1.65500 1.25025 7

-10 2 -3 0.56469 1.07615 2

10 2 3 1.61031 1.32358 6

-10 2 -3 1.28093 1.60234 1

-10 2 -2 19.1430 2.04473 1

10 -2 -2 18.9541 1.61440 7

10 2 2 18.2407 1.56765 6

-10 2 -2 18.9794 1.37803 2

-10 2 -1 2.21392 1.10981 2

10 -2 -1 5.52952 1.29591 7

10 2 1 4.04317 1.29885 6

-10 2 -1 2.60552 1.84535 1

10 -2 0 9.70517 1.33988 7

10 2 0 10.9325 1.42105 6

-10 2 0 8.05730 1.15959 2

-10 2 0 4.32659 2.00057 1

-10 2 1 2.85748 1.11361 2

10 -2 1 2.43405 1.19479 7

10 2 -1 3.73731 1.26137 6

-10 2 1 2.80705 2.04098 1

-10 2 2 19.2274 2.28440 1

10 -2 2 20.3087 1.61651 7

10 2 -2 20.8826 1.61270 6

10 -2 3 0.14625 1.16960 7

10 2 -3 1.12069 1.28268 6

-10 2 3 1.39410 1.70373 1

-10 2 4 0.52564 1.55599 1

10 -2 4 0.37962 1.25642 7

-10 2 5 6.53913 1.59694 1

10 -2 5 6.48097 1.38942 7

10 2 -6 8.37872 1.55575 8

-10 2 6 9.06621 1.52398 4

10 -2 6 7.04691 1.47397 7

10 2 -7 21.7068 1.89943 8

10 -2 7 18.7020 1.80023 7

-10 2 7 20.3230 1.77736 4

10 2 -8 5.59545 1.74881 8

-10 2 8 5.78124 1.53843 4

10 -2 8 5.69543 1.57129 7

10 2 -9 1.88972 1.91588 8

-10 2 9 3.85582 1.60995 4

11 2 7 7.81235 2.00780 6

11 2 6 1.04985 1.73326 6

11 -2 -5 2.52944 1.60858 7

11 2 5 4.21938 1.71071 6

-11 2 -4 4.13842 1.69300 1

11 -2 -4 2.57481 1.54044 7

11 2 4 2.84666 1.53895 6

-11 2 -3 20.3853 2.08906 1

11 -2 -3 20.1075 1.81890 7

11 2 3 21.2869 1.79647 6

-11 2 -2 20.8925 2.19530 1

11 -2 -2 22.0787 1.74312 7

11 2 2 24.3213 1.77909 6

11 -2 -1 1.80319 1.34593 7

11 2 1 1.50296 1.35748 6

-11 2 -1 1.93255 1.99870 1

11 -2 0 2.39525 1.33386 7

11 2 0 0.69256 1.31969 6

11 -2 1 1.67171 1.29600 7

11 2 -1 1.95946 1.35832 6

-11 2 1 1.41924 2.11173 1

-11 2 2 25.1939 2.42516 1

11 -2 2 24.1453 1.72927 7

11 2 -2 21.3636 1.72567 6

-11 2 3 19.6094 2.20849 1

11 -2 3 21.9414 1.76527 7

11 -2 4 3.26079 1.37104 7

-11 2 4 3.07938 1.66462 1

-11 2 5 1.73292 1.57639 1

11 -2 5 2.14847 1.37266 7

11 -2 6 0.39857 1.39674 7

11 -2 7 6.93748 1.57111 7

12 -2 -5 1.00917 1.66142 7

12 2 5 2.30436 1.74867 6

-12 2 -4 7.04923 1.80207 1

12 -2 -4 9.98538 1.72667 7

12 2 4 9.83135 1.78861 6

12 -2 -3 9.10170 1.65928 7

12 2 3 7.24398 1.63669 6

-12 2 -3 9.81729 1.92508 1

12 -2 -2 1.11131 1.42074 7

12 2 2 4.06039 1.53584 6

-12 2 -2 5.49468 2.02062 1

12 -2 -1 22.0194 1.78428 7

12 2 1 18.6575 1.75841 6

-12 2 -1 17.6528 2.33293 1

12 -2 0 2.17772 1.44172 7

12 2 0 1.96613 1.42667 6

12 -2 1 22.6863 1.75661 7

12 2 -1 19.6657 1.73911 6

-12 2 1 19.2946 2.50309 1

12 -2 2 3.07418 1.43844 7

-12 2 2 0.47590 1.97630 1

12 -2 3 8.47139 1.52044 7

-12 2 3 8.58536 2.02662 1

12 -2 4 8.57168 1.57942 7

-12 2 4 7.69591 1.83032 1

12 -2 5 3.53218 1.48886 7

12 -2 6 20.3631 1.82710 7

13 -2 -2 5.83152 1.64038 7

13 2 2 5.56644 1.69069 6

-13 2 -2 6.38376 2.06336 1

13 -2 -1 6.33816 1.62480 7

13 2 1 7.92731 1.65952 6

-13 2 -1 5.38766 2.17977 1

13 -2 0 2.77503 1.52677 7

13 2 0 0.25366 1.49196 6

-13 2 0 4.47945 2.22245 1

13 -2 1 6.48690 1.57432 7

-13 2 1 7.12569 2.39365 1

13 -2 2 7.97186 1.57590 7

-13 2 2 6.69064 2.22699 1

13 -2 3 4.86691 1.53442 7

-13 2 3 6.18017 2.08398 1

0 3 0-0.15089 1.08600 1

0 3 -1 1.67135 1.09325 1

0 3 1 0.79028 1.07507 1

0 -3 -1 0.48218 0.89536 5

0 3 2-0.79773 1.13340 1

0 3 2 0.33091 0.73790 4

0 3 2-0.01735 0.95937 3

0 -3 -2-0.40012 0.92352 5

0 3 -2 0.12567 1.12437 1

0 3 3 10.0334 1.06943 3

0 3 -3 9.93851 1.30912 1

0 3 3 5.68934 1.19090 1

0 -3 -3 9.08922 1.08500 5

0 3 3 8.81580 0.89452 4

0 3 4-0.25975 0.80754 4

0 3 4-0.68642 0.94942 3

0 -3 -4 0.75708 0.92218 5

0 3 -4-0.62637 1.09221 1

0 3 4-0.22278 1.02919 1

0 3 5 215.814 5.31399 3

0 3 -5 194.747 5.45656 1

0 3 5 195.209 5.39230 1

0 3 5 204.405 5.21405 4

0 3 6 1.70587 0.97172 4

0 3 6 1.24084 1.07691 3

0 3 7 15.8759 1.43136 3

0 3 7 13.2952 1.26433 4

0 3 8 2.65480 1.22222 4

0 3 8 1.64410 1.32797 3

0 3 9 19.7213 1.77245 3

0 3 9 16.5661 1.59614 4

0 3 10 2.38760 1.46584 4

0 3 10-0.29685 1.64119 3

0 3 11 2.22330 1.63902 4

0 3 11 0.94623 1.87504 3

1 3 11 4.65431 2.00958 3

1 3 10 1.49506 1.70577 3

1 3 9 6.82638 1.56095 3

1 3 8 39.1768 2.01657 3

1 3 7 2.11174 1.19917 3

1 3 6 1.00618 1.10245 3

1 3 5 50.5934 1.90522 3

-1 3 -5 36.6584 1.90690 1

-1 3 -4 1.53976 1.11093 1

1 3 4 1.36545 0.95141 3

1 3 3 23.9371 1.36468 3

-1 3 -3 19.5737 1.50318 1

1 -3 -3 30.5679 1.51834 5

-1 3 -2 1.07987 1.06050 1

1 3 2 1.81105 0.97686 3

1 -3 -2 2.40380 0.99537 5

-1 3 -1 2.60368 1.08517 1

1 3 1 4.37887 1.18653 1

1 -3 -1 4.36983 0.98114 5

1 3 0 3.40619 1.17728 1

1 -3 0 2.04203 0.93688 5

-1 3 0 2.27295 1.06026 1

-1 3 1 4.26129 1.11337 1

1 -3 1 3.61414 0.78578 7

1 3 -1 3.19068 1.15141 1

-1 -3 -1 4.43106 0.95483 5

-1 3 2 1.60439 1.09767 1

-1 3 2 1.56587 0.83082 4

1 -3 2 0.25203 0.84249 7

-1 3 2 1.77351 1.02150 3

-1 -3 -2 1.84065 0.90414 5

-1 3 3 42.6573 1.70860 3

-1 3 3 33.9744 1.73380 1

-1 -3 -3 21.7313 1.47305 5

-1 3 3 33.1420 1.54576 4

-1 3 4 1.66504 0.87576 4

-1 3 4 1.49818 0.98612 3

-1 3 4 0.93353 1.01034 1

-1 -3 -4 1.22757 0.97113 5

-1 3 5 54.4030 1.96992 3

-1 3 5 44.6944 1.96610 1

-1 3 5 44.4403 1.81408 4

-1 3 6 1.29844 0.99197 4

-1 3 6 1.14371 1.11598 3

-1 3 7 1.20401 1.09629 4

-1 3 7 0.52094 1.16562 3

-1 3 8 38.4278 1.97597 3

-1 3 8 36.9026 1.90877 4

-1 3 9 5.49035 1.40575 4

-1 3 9 4.73651 1.49293 3

-1 3 10 4.56494 1.53764 4

-1 3 10-0.29279 1.66399 3

-1 3 11 5.22574 1.70989 4

-1 3 11 2.03280 1.85990 3

-1 3 12 13.3703 1.99849 4

2 3 11-0.68396 1.92777 3

2 3 10 7.83791 1.81164 3

2 3 9 10.7805 1.68308 3

2 3 8 1.20086 1.33723 3

2 3 7 5.05000 1.28669 3

2 3 6 3.21916 1.11340 3

2 3 5 6.07650 1.10003 3

-2 3 -5 1.77996 1.11459 1

2 3 4 1.26901 0.98242 3

2 3 4 0.85961 0.94121 6

-2 3 -4 0.59629 1.09271 1

2 3 3 0.31970 0.95693 3

2 3 3 1.23452 0.93700 6

-2 3 -3-0.42397 1.11023 1

2 -3 -3 1.31517 0.95994 5

2 3 2 100.301 2.92959 6

-2 3 -2 98.1124 3.26951 1

2 -3 -2 96.8003 3.00258 5

2 -3 -1 128.204 3.34715 7

-2 3 -1 108.753 3.72549 1

2 -3 -1 131.250 3.60346 5

2 -3 0 5.94594 0.94397 7

-2 3 0 3.26326 1.24184 1

2 -3 0 2.38412 0.98058 5

2 -3 1 118.337 3.26736 7

-2 3 1 111.106 3.60700 1

2 -3 1 120.041 3.38046 5

2 -3 2 98.6239 2.87398 7

-2 3 2 91.2791 3.11232 1

-2 -3 -2 96.3990 2.93018 5

-2 3 3 0.79010 0.89825 4

2 -3 3 0.41434 0.89240 7

-2 3 3 0.58162 0.99414 3

-2 3 3 1.72687 1.08331 1

-2 -3 -3 0.67355 0.98009 5

-2 3 4 0.21115 0.90917 4

-2 3 4 2.28511 0.98067 3

-2 -3 -4 0.93126 0.97332 5

-2 3 4 0.45125 1.02340 1

-2 3 5 2.04685 0.98030 4

-2 3 5 3.22990 1.03933 3

-2 -3 -5 4.03608 1.04605 5

-2 3 5 2.84322 0.99094 1

-2 3 6 2.37446 1.05100 4

-2 3 6 3.01668 1.14793 3

-2 3 7 4.16289 1.20681 4

-2 3 7 5.07826 1.25412 3

-2 3 8 2.27416 1.24200 4

-2 3 8 0.56710 1.28403 3

-2 3 9 9.06157 1.57185 3

-2 3 9 11.3158 1.55829 4

-2 3 10 10.1849 1.69334 4

-2 3 10 8.54659 1.73770 3

-2 3 11 0.46297 1.63679 4

-2 3 11 0.68220 1.81936 3

3 3 11 5.74228 2.00938 3

3 3 10 0.93724 1.66810 3

3 3 9 16.0872 1.72517 3

3 3 8 0.78060 1.33025 3

3 3 7 22.3320 1.54894 3

3 3 6 1.34163 1.14305 3

3 3 5 5.20671 1.07804 3

-3 3 -5 4.01100 1.20043 1

3 3 5 3.97643 1.07105 6

3 3 4 67.0192 2.26127 3

3 3 4 66.0373 2.28613 6

-3 3 -4 68.0580 2.61206 1

-3 3 -3 5.33214 1.28460 1

3 3 3 5.04726 1.06737 3

3 3 3 5.56083 1.06166 6

3 -3 -2 24.7566 1.25072 7

3 3 2 25.8886 1.38379 6

-3 3 -2 24.8219 1.66518 1

3 -3 -2 24.4011 1.40355 5

-3 3 -1 3.23774 1.26795 1

3 -3 -1 4.97871 0.89382 7

3 3 1 3.95412 1.01685 6

3 -3 -1 5.34225 1.05188 5

3 -3 0 49.0109 1.79984 7

3 3 0 49.5854 1.90092 6

-3 3 0 46.4913 2.18534 1

3 -3 0 52.9850 1.94693 5

-3 3 1 5.92655 1.28472 1

3 -3 1 1.90127 0.87862 7

3 -3 1 2.80690 0.92384 5

3 -3 2 26.0934 1.40354 7

-3 3 2 25.4517 1.65299 1

3 -3 2 23.6652 1.35038 5

-3 -3 -2 25.3463 1.43438 5

-3 3 3 4.39610 1.01686 4

-3 3 3 4.48411 1.21327 1

3 -3 3 4.17019 1.03012 7

-3 3 3 5.77523 1.09335 3

-3 -3 -3 4.66439 1.05060 5

3 -3 4 60.8958 2.30615 7

-3 3 4 66.1536 2.21789 3

-3 3 4 61.1316 2.31773 1

-3 -3 -4 59.3507 2.23271 5

-3 3 4 60.6633 2.22937 4

-3 3 5 3.75457 1.07198 4

-3 3 5 4.95254 1.09866 3

-3 3 5 4.33042 1.10321 1

-3 -3 -5 4.41387 1.06099 5

-3 3 6 1.37583 1.08173 4

-3 3 6 1.75431 1.13728 3

-3 3 7 20.3389 1.49243 3

-3 3 7 19.1377 1.53659 4

-3 3 8 2.37459 1.32067 4

-3 3 8 3.06636 1.32423 3

-3 3 9 16.3353 1.66591 3

-3 3 9 17.7181 1.70378 4

-3 3 10 3.90618 1.61091 4

-3 3 10 0.36055 1.59012 3

-3 3 11 9.33739 1.85378 4

-3 3 11 4.81887 1.81733 3

4 3 11 0.91399 1.86659 3

4 3 10 2.14330 1.67689 3

4 3 9 1.84754 1.46723 3

4 3 8 28.0635 1.77981 3

4 3 7 6.94419 1.29849 3

4 3 6 14.3650 1.31181 3

-4 3 -5 0.50440 1.16156 1

4 3 5 0.64770 1.01103 3

4 3 5 0.34591 1.07573 6

4 3 4 11.4148 1.16797 3

4 3 4 11.2841 1.23204 6

-4 3 -4 10.6105 1.41824 1

4 -3 -3 89.3388 2.75616 7

4 3 3 93.7556 2.80861 3

-4 3 -3 91.6880 3.17784 1

4 3 3 91.3511 2.87510 6

4 -3 -2 13.3726 1.09653 7

-4 3 -2 12.3396 1.52908 1

4 3 2 13.1088 1.21763 6

4 -3 -1 0.43229 0.86940 7

4 3 1 1.79772 1.01100 6

4 -3 -1 0.95618 0.94396 5

-4 3 -1 1.41714 1.30741 1

4 -3 0-0.24655 0.87140 7

4 3 0 0.79139 0.99976 6

4 -3 0 1.25916 0.91452 5

-4 3 0 0.27507 1.26585 1

4 -3 1 0.22452 0.89778 7

4 3 -1 1.09093 1.02485 6

4 -3 1 0.41132 0.91618 5

-4 3 1 1.40292 1.25862 1

4 -3 2 11.6902 1.17060 7

-4 3 2 13.2358 1.47865 1

4 -3 2 11.5274 1.10293 5

-4 -3 -2 12.0807 1.19396 5

-4 3 3 92.0198 2.83428 4

-4 3 3 91.9443 2.74361 3

4 -3 3 84.2629 2.80390 7

-4 3 3 87.7353 3.03213 1

-4 -3 -3 83.3937 2.75823 5

4 -3 3 80.8292 2.66993 5

-4 3 4 9.84223 1.15532 3

4 -3 4 9.16213 1.22834 7

-4 3 4 11.0100 1.32825 1

-4 -3 -4 9.79675 1.19851 5

-4 3 4 10.0852 1.23954 4

-4 3 5-0.37193 1.07780 4

-4 3 5 0.99463 1.05635 1

4 -3 5-0.02563 1.14780 7

-4 3 5 1.09783 1.06024 3

-4 3 6 15.7574 1.34910 3

-4 3 6 15.5682 1.40132 4

-4 3 7 3.43208 1.26445 4

-4 3 7 5.82361 1.27602 3

-4 3 8 25.3748 1.70388 3

-4 3 8 23.4275 1.77795 4

-4 3 9 2.01962 1.48301 4

-4 3 9 0.45766 1.39443 3

-4 3 10 2.57371 1.61350 4

-4 3 10 3.12583 1.55749 3

-4 3 11 0.31431 1.72977 4

-4 3 11 1.29073 1.70737 3

5 3 7 29.0003 1.72498 3

5 3 6 1.87368 1.11890 3

5 3 5 0.13846 1.05708 3

5 3 5 1.76067 1.16740 6

-5 3 -5 2.31569 1.28957 1

5 -3 -4 6.44126 1.10292 7

5 3 4 5.64055 1.09629 3

5 3 4 4.92714 1.21762 6

-5 3 -4 6.84804 1.39938 1

5 -3 -3 0.83967 0.95279 7

5 3 3-1.25891 1.02697 3

5 3 3-0.16889 1.07052 6

-5 3 -3 1.14832 1.35680 1

5 -3 -2 25.4870 1.41427 7

-5 3 -2 23.5752 1.91749 1

5 3 2 27.6923 1.57061 6

5 -3 -1 17.4423 1.31889 7

-5 3 -1 16.3247 1.82921 1

5 3 1 28.4922 1.54089 6

5 -3 0 194.934 4.98860 7

-5 3 0 164.171 5.43100 1

5 3 0 187.649 5.04776 6

5 -3 0 194.906 4.94378 5

5 -3 1 14.2955 1.28159 7

-5 3 1 17.4604 1.83625 1

5 3 -1 18.2981 1.43343 6

5 -3 1 13.1472 1.23648 5

5 -3 2 23.4555 1.45233 7

-5 3 2 26.2116 1.96150 1

5 3 -2 23.8436 1.54165 6

5 -3 2 23.3439 1.33714 5

5 -3 3 0.73450 1.07403 7

-5 3 3 2.29282 1.36029 1

-5 -3 -3 0.73004 0.98745 5

-5 3 4 5.36139 1.21908 4

5 -3 4 5.04649 1.24826 7

-5 3 4 6.42704 1.11032 3

-5 3 4 7.01925 1.37710 1

-5 3 5 2.71265 1.20427 4

5 -3 5-0.48439 1.23846 7

-5 3 5 1.54382 1.06277 3

-5 3 5 2.76520 1.19619 1

-5 3 6 3.45884 1.26123 4

5 -3 6 1.03986 1.35590 7

-5 3 6 3.50046 1.17388 3

5 -3 7 23.3558 1.97459 7

-5 3 7 29.2562 1.68670 3

-5 3 7 27.0004 1.76296 4

-5 3 8 2.38008 1.38682 4

5 -3 8 0.24449 1.59440 7

-5 3 8 3.71119 1.32117 3

-5 3 9 3.54035 1.52998 4

-5 3 9 2.28625 1.39188 3

-5 3 10 5.53651 1.67078 4

6 3 6 1.83191 1.30882 6

6 3 5 2.27727 1.26576 6

-6 3 -5 2.52424 1.41179 1

6 -3 -4 26.2754 1.58305 7

-6 3 -4 19.8912 1.80863 1

6 3 4 24.4720 1.64350 6

6 -3 -3 2.03856 1.04975 7

6 3 3 2.23047 1.21984 6

-6 3 -3 3.37792 1.52141 1

6 -3 -2 1.49900 1.06361 7

6 3 2 0.78669 1.12648 6

-6 3 -2 1.05863 1.54470 1

6 -3 -1 1.39060 1.03162 7

6 3 1 1.90142 1.18249 6

-6 3 -1 0.03726 1.56598 1

6 -3 0 0.30620 0.96670 7

6 3 0-0.42405 1.10944 6

-6 3 0 1.04583 1.60958 1

6 -3 1 1.05811 1.05581 7

6 3 -1 1.65872 1.16352 6

-6 3 1 0.95674 1.62271 1

6 -3 2 0.86716 1.08317 7

6 3 -2 0.40040 1.19746 6

-6 3 2 0.97687 1.60991 1

6 -3 3 3.12219 1.17029 7

-6 3 3 3.32401 1.54495 1

-6 3 4 23.1616 1.64874 4

6 -3 4 22.7841 1.65972 7

-6 3 4 24.9444 1.87138 1

-6 3 5 0.55176 1.23980 4

6 -3 5 1.31421 1.30871 7

-6 3 5 0.72786 1.22132 1

-6 3 6-0.23444 1.29454 4

6 -3 6-1.43188 1.44391 7

6 -3 7 15.5220 1.85501 7

-6 3 7 16.1628 1.65086 4

-6 3 8 2.12009 1.42751 4

6 -3 8-1.11562 1.69682 7

-6 3 9 7.96476 1.63844 4

6 -3 9 8.08267 1.87671 7

6 -3 10 18.3103 2.22540 7

-6 3 10 25.8006 2.11647 4

7 3 6 53.5818 2.44211 6

7 -3 -5 1.19138 1.26924 7

7 3 5 2.02887 1.38212 6

-7 3 -5 2.36873 1.49269 1

7 -3 -4 33.7696 1.83261 7

-7 3 -4 29.4242 2.11074 1

7 3 4 32.5832 1.85527 6

7 -3 -3 25.5457 1.60714 7

-7 3 -3 21.3464 1.95982 1

7 3 3 23.2213 1.68199 6

7 -3 -2 1.59705 1.13972 7

-7 3 -2 2.61062 1.67545 1

7 3 2 1.10787 1.24343 6

-7 3 -1 12.6302 1.98722 1

7 -3 -1 7.66294 1.21509 7

7 3 1 10.4536 1.36626 6

7 -3 0 6.60090 1.21698 7

-7 3 0 2.12907 1.80159 1

7 3 0 5.12846 1.28404 6

7 -3 1 12.7395 1.36891 7

-7 3 1 5.13301 1.80915 1

7 3 -1 6.89355 1.31241 6

7 -3 2 0.47614 1.14269 7

-7 3 2-0.47827 1.67659 1

7 3 -2 1.44477 1.25598 6

7 -3 3 25.4696 1.64996 7

-7 3 3 24.3282 2.07960 1

7 3 -3 22.0992 1.69450 6

7 -3 4 30.4581 1.84055 7

-7 3 4 33.0690 2.11279 1

-7 3 5 1.13193 1.29596 4

7 -3 5 2.39690 1.40660 7

-7 3 5 1.50568 1.42656 1

7 -3 6 49.7673 2.47321 7

-7 3 6 52.8134 2.38160 4

-7 3 7 10.1282 1.60038 4

7 -3 7 6.82222 1.66159 7

-7 3 8 3.44094 1.57593 4

7 -3 8 2.83087 1.72270 7

-7 3 9 1.22691 1.58088 4

7 -3 9-1.31089 1.81715 7

8 -3 -6 10.5760 1.62451 7

8 3 6 14.3696 1.78479 6

8 -3 -5 4.71084 1.44360 7

-8 3 -5 4.66845 1.61077 1

8 3 5 5.81851 1.52136 6

8 -3 -4 34.0973 1.93588 7

-8 3 -4 27.5710 2.15539 1

8 3 4 34.3768 2.01185 6

8 -3 -3 3.68352 1.28702 7

-8 3 -3 2.01117 1.68450 1

8 3 3 3.09227 1.38157 6

8 -3 -2 2.09806 1.21671 7

-8 3 -2-0.12061 1.74631 1

8 3 2 1.29175 1.33112 6

8 -3 -1 1.67513 1.20942 7

-8 3 -1 0.30607 1.87036 1

8 -3 0 0.42323 1.21053 7

-8 3 0-0.51560 1.87796 1

8 3 0 0.43760 1.27021 6

8 -3 1 0.50518 1.16177 7

-8 3 1 0.84976 1.99611 1

8 3 -1 1.85450 1.30353 6

8 -3 2 0.82434 1.21186 7

-8 3 2-1.82238 1.87384 1

8 3 -2 2.79726 1.34564 6

8 -3 3 1.72642 1.25752 7

-8 3 3 0.63121 1.74859 1

8 3 -3 3.12106 1.43471 6

8 -3 4 30.5453 1.93146 7

-8 3 4 33.9133 2.24449 1

8 -3 5 4.45821 1.45283 7

-8 3 5 4.29173 1.58122 1

8 -3 6 12.2425 1.68088 7

-8 3 6 12.6297 1.62030 4

-8 3 7 3.15504 1.55798 4

8 -3 7 1.19502 1.54125 7

-8 3 8 1.74504 1.58564 4

8 -3 8 1.71414 1.69953 7

9 3 7 5.39389 1.79005 6

9 -3 -6 2.89781 1.54986 7

9 3 6 4.61286 1.69492 6

9 -3 -5 1.37427 1.51152 7

9 3 5 2.72289 1.57015 6

9 -3 -4 3.63101 1.43480 7

-9 3 -4 0.79713 1.63132 1

9 3 4 3.49007 1.53678 6

9 -3 -3 22.7844 1.77348 7

-9 3 -3 19.4849 2.18832 1

9 3 3 22.1305 1.80871 6

9 -3 -2 13.2804 1.56912 7

-9 3 -2 10.5113 2.05542 1

9 3 2 13.5570 1.61993 6

9 -3 -1 42.2261 2.04923 7

-9 3 -1 31.9516 2.69781 1

9 3 1 41.3782 2.09436 6

9 -3 0 20.3480 1.65716 7

-9 3 0 13.8423 2.46961 1

9 3 0 22.4623 1.77385 6

-9 3 1 29.2696 2.85900 1

9 -3 1 43.5079 2.12289 7

9 3 -1 41.7070 2.15457 6

9 -3 2 10.0485 1.46774 7

-9 3 2 12.9927 2.28730 1

9 3 -2 10.3955 1.59342 6

9 -3 3 20.4094 1.67980 7

-9 3 3 20.7766 2.27644 1

9 3 -3 19.3476 1.74769 6

9 -3 4 0.77150 1.38836 7

-9 3 4 2.28040 1.73850 1

9 3 -4 1.91539 1.50915 6

9 -3 5 2.59871 1.46231 7

-9 3 5 1.34487 1.60350 1

9 -3 6 4.38434 1.53264 7

-9 3 7 0.45801 1.51844 4

9 -3 7 1.32614 1.61865 7

10 -3 -6 0.35234 1.59392 7

10 3 6 1.42601 1.76712 6

10 -3 -5 2.07403 1.60319 7

10 3 5 4.42958 1.69513 6

10 -3 -4 4.24567 1.57062 7

-10 3 -4 2.54025 1.74962 1

10 3 4 4.52010 1.61874 6

10 -3 -3 14.4645 1.69312 7

-10 3 -3 12.4407 2.02959 1

10 3 3 14.0312 1.73117 6

-10 3 -2 9.62336 2.14634 1

10 -3 -2 10.8218 1.57738 7

10 3 2 8.76824 1.62915 6

-10 3 -1 2.65962 2.15612 1

10 -3 -1 6.02709 1.45612 7

10 3 1 6.26650 1.55815 6

10 -3 0 1.43637 1.37090 7

10 3 0 1.81584 1.49026 6

-10 3 1 3.07716 2.39919 1

10 -3 1 5.99743 1.47241 7

10 3 -1 6.08948 1.55885 6

-10 3 2 6.47668 2.28183 1

10 -3 2 10.8977 1.61892 7

10 3 -2 11.9391 1.65731 6

10 -3 3 13.0206 1.59449 7

-10 3 3 13.5179 2.18981 1

10 3 -3 14.5780 1.75153 6

10 -3 4 3.49067 1.46211 7

-10 3 4 1.40137 1.85021 1

10 3 -4 3.55747 1.58689 6

10 -3 5 3.04863 1.49194 7

-10 3 5 4.74419 1.75742 1

10 -3 6 0.03253 1.56665 7

11 -3 -4 3.97018 1.66785 7

-11 3 -4 2.84909 1.85833 1

-11 3 -3 5.30831 1.99958 1

11 -3 -3 6.32560 1.62164 7

11 3 3 7.36595 1.69879 6

11 -3 -2 23.6021 1.91832 7

-11 3 -2 16.5876 2.39303 1

11 3 2 23.5061 1.99041 6

-11 3 -1 2.90323 2.28798 1

11 -3 -1 5.03927 1.54401 7

11 3 1 2.68534 1.55148 6

-11 3 0-1.78230 2.39057 1

11 -3 0 3.70391 1.50326 7

11 3 0 2.99268 1.57696 6

-11 3 1-0.48480 2.57752 1

11 -3 1 4.60895 1.51734 7

11 3 -1 4.39540 1.61279 6

11 -3 2 20.0483 1.79716 7

-11 3 2 18.7788 2.63372 1

11 3 -2 20.0021 1.88053 6

-11 3 3 3.77452 2.09970 1

11 -3 3 6.35759 1.61900 7

11 3 -3 6.46388 1.66148 6

-11 3 4 3.59692 1.95385 1

0 -4 0 298.447 7.15728 5

0 4 0 236.272 7.36923 1

0 4 -1-1.18593 1.60394 1

0 -4 -1 9.16806 1.51653 5

0 4 1 8.68812 1.78980 1

0 -4 1 0.37204 1.32536 5

0 4 -2 13.4441 1.83268 1

0 -4 -2 14.9622 1.52120 5

0 4 2 13.4636 1.77609 1

0 -4 -3 0.36597 1.22658 5

0 -4 -4 9.17431 1.38578 5

0 4 5 0.47118 1.33456 3

0 4 6 2.50915 1.37860 3

0 4 7 2.64369 1.44541 3

0 4 8 3.54304 1.47681 3

0 4 9 4.90917 1.62501 3

1 4 9 4.29073 1.62421 3

1 4 8 6.72859 1.58115 3

1 4 7 6.80215 1.48718 3

1 4 6 0.96696 1.36489 3

1 4 5 1.26579 1.33334 3

-1 4 -5 1.94601 1.55721 1

-1 4 -4 5.41358 1.68079 1

1 -4 -3 24.8366 1.75535 5

-1 4 -3 24.3300 2.06977 1

-1 4 -2 7.03882 1.71846 1

1 -4 -2 6.97248 1.38619 5

-1 4 -1 1.09248 1.57533 1

1 -4 -1-1.11167 1.34387 5

1 -4 0-0.15418 1.24383 5

-1 4 0 0.97750 1.60889 1

-1 4 1 0.85001 1.53156 1

-1 -4 -1 1.18958 1.24970 5

1 -4 1 0.68315 1.27329 5

-1 4 2 7.86829 1.63607 1

-1 -4 -2 7.70553 1.41436 5

1 -4 2 7.20875 1.45627 5

-1 -4 -3 28.6322 1.75149 5

-1 4 3 22.7469 1.90460 1

-1 4 4 8.74330 1.56610 1

-1 -4 -4 8.21280 1.38176 5

-1 4 5 2.05330 1.35500 3

-1 4 5 2.83718 1.42127 1

-1 4 6 2.22699 1.40108 3

-1 4 7 10.4545 1.53802 3

-1 4 8 6.08082 1.50626 3

-1 4 9 5.44213 1.59919 3

2 4 8 4.96997 1.52916 3

2 4 7 31.6249 1.95580 3

2 4 6 19.3266 1.69057 3

2 4 5 5.69005 1.42390 3

-2 4 -5 3.37309 1.53270 1

-2 4 -4 36.3482 2.26425 1

2 -4 -3 13.3984 1.48303 5

-2 4 -3 15.7988 1.87307 1

-2 4 -2 12.7210 1.84097 1

2 -4 -2 3.84833 1.30074 5

-2 4 -1 3.61149 1.62344 1

2 -4 -1 1.99291 1.25161 5

-2 4 0-0.24961 1.51827 1

2 -4 0 1.25218 1.28326 5

-2 -4 0 2.58922 1.28921 5

-2 4 1 3.62399 1.57087 1

2 -4 1 1.86923 1.24928 5

-2 -4 -1 1.02676 1.16638 5

-2 4 2 4.12827 1.56465 1

-2 -4 -2 3.89899 1.30104 5

-2 4 3 15.9291 1.74525 1

-2 -4 -3 15.2719 1.56807 5

2 -4 3 14.6744 1.60112 5

-2 -4 -4 40.2912 2.07357 5

-2 4 4 36.8715 2.15383 1

-2 4 5 6.25361 1.45154 1

-2 4 5 6.76752 1.48174 3

-2 -4 -5 4.51494 1.34098 5

-2 4 6 17.9868 1.64600 3

2 -4 6 15.5606 1.76505 7

-2 4 7 31.5255 1.93424 3

-2 4 8 5.09814 1.50236 3

-2 4 9 6.26853 1.58580 3

3 4 8 3.45114 1.51962 3

3 4 7 4.60260 1.45244 3

3 4 6 7.57284 1.47585 3

-3 4 -5 8.73515 1.65023 1

3 4 5 8.84249 1.46940 3

-3 4 -4 6.17398 1.63240 1

-3 4 -3 9.50163 1.74049 1

-3 4 -2 13.8620 1.83835 1

3 -4 -2 15.7369 1.49707 5

3 -4 -1 14.6201 1.48366 5

-3 4 -1 11.2690 1.81761 1

3 -4 0-0.32446 1.23200 5

-3 4 0 1.47479 1.54854 1

-3 4 1 11.3645 1.77732 1

3 -4 1 14.4166 1.51245 5

-3 -4 -1 11.6275 1.42884 5

-3 4 2 16.1568 1.79478 1

3 -4 2 16.0257 1.56858 5

-3 -4 -2 14.9482 1.54398 5

-3 4 3 8.64311 1.61396 1

-3 -4 -3 8.61927 1.49042 5

3 -4 3 9.29484 1.43567 5

-3 4 4 3.85186 1.44721 1

3 -4 4 4.79461 1.39661 7

-3 -4 -4 5.55252 1.40364 5

-3 4 5 8.07907 1.44885 1

3 -4 5 9.07870 1.55051 7

-3 4 5 10.4736 1.50263 3

-3 -4 -5 8.97051 1.45360 5

3 -4 6 8.32206 1.68568 7

-3 4 6 9.90233 1.53225 3

3 -4 7 1.65630 1.69673 7

-3 4 7 5.74640 1.46422 3

3 -4 8 1.23485 1.81683 7

-3 4 8 4.67194 1.51875 3

4 4 8 27.2533 1.91546 3

4 4 7 0.82056 1.43383 3

4 4 6-0.09453 1.38350 3

4 4 5-0.98694 1.39093 3

-4 4 -4 0.96854 1.54839 1

4 4 3 22.3895 1.70363 6

-4 4 -3 20.0450 1.98842 1

-4 4 -2 12.1782 1.91532 1

-4 4 -1 15.6143 1.98057 1

4 -4 -1 21.2142 1.60763 5

-4 4 0 0.76728 1.66326 1

4 -4 0 0.31850 1.18594 5

-4 4 1 20.0632 2.04346 1

4 -4 1 17.0216 1.55921 5

-4 -4 -1 16.5271 1.61682 5

-4 4 2 11.4391 1.81057 1

4 -4 2 13.3196 1.50697 5

-4 -4 -2 11.1959 1.47872 5

4 -4 3 21.2687 1.65330 7

-4 4 3 18.2596 1.89753 1

-4 -4 -3 24.5393 1.81413 5

4 -4 4 0.78112 1.32597 7

-4 4 4 2.25432 1.48415 1

-4 -4 -4 1.97687 1.33913 5

4 -4 5 2.37403 1.51750 7

-4 4 5-0.04760 1.41410 3

-4 4 5 3.00079 1.37897 1

-4 4 6 0.91609 1.34532 4

4 -4 6-0.66835 1.65119 7

-4 4 6-0.28486 1.37831 3

-4 4 7 0.93219 1.42038 4

4 -4 7-0.10843 1.71864 7

-4 4 7 2.24959 1.42458 3

4 -4 8 24.7263 2.37199 7

-4 4 8 24.1094 1.83568 3

-4 4 8 22.5391 1.88516 4

-5 4 -4 8.60321 1.79518 1

-5 4 -3 8.94393 1.80948 1

5 4 3 10.1339 1.57905 6

-5 4 -2 10.8931 1.92252 1

5 4 2 14.0821 1.61174 6

-5 4 -1 2.63219 1.82700 1

-5 4 0 0.65072 1.74699 1

5 -4 0 0.63101 1.17488 5

-5 4 1 2.79927 1.80243 1

5 -4 2 13.5033 1.44752 7

-5 4 2 14.0128 1.98100 1

-5 -4 -2 14.0977 1.53486 5

5 -4 3 9.11051 1.43513 7

-5 4 3 9.60015 1.78912 1

5 -4 4 8.52665 1.54383 7

-5 4 4 6.58741 1.60465 1

5 -4 5 40.7993 2.28029 7

-5 4 5 34.5114 2.10388 1

-5 4 6 2.43054 1.44319 4

5 -4 6 0.24118 1.64681 7

-5 4 6 1.29561 1.39781 3

-5 4 7 5.27729 1.55226 4

5 -4 7 3.42391 1.83861 7

-5 4 8 5.48765 1.60341 4

-6 4 -4 2.12126 1.62702 1

6 4 4 5.81228 1.55239 6

-6 4 -3 3.46446 1.77650 1

6 4 3 2.17200 1.48582 6

-6 4 -2 10.7145 1.95143 1

6 4 2 13.4650 1.68197 6

-6 4 -1 11.7577 2.01042 1

6 4 1 12.1580 1.62945 6

-6 4 0 1.86361 1.82949 1

6 -4 1 10.1391 1.43489 7

-6 4 1 12.4528 2.09433 1

6 -4 2 8.88030 1.44293 7

-6 4 2 14.2511 2.08945 1

-6 4 3 1.38691 1.69154 1

6 -4 3 1.57907 1.36186 7

6 -4 4 5.83071 1.57278 7

-6 4 4 16.5054 1.92749 1

6 -4 5 15.4487 1.78962 7

-6 4 5 13.2687 1.74538 1

6 -4 6 5.08105 1.76236 7

-6 4 7 2.69581 1.56074 4

-7 4 -4 4.24991 1.71748 1

7 4 4-0.11736 1.50624 6

-7 4 -3 3.42608 1.81736 1

7 4 3 4.41887 1.56664 6

7 4 2 17.3989 1.76033 6

-7 4 -2 15.7535 2.14003 1

7 4 1 53.8284 2.47420 6

-7 4 -1 50.5116 2.96734 1

-7 4 0 6.94471 2.08646 1

7 -4 0 2.79107 1.40611 7

7 4 0 9.23829 1.67522 6

7 -4 1 57.6112 2.41359 7

-7 4 1 51.1159 3.03198 1

7 -4 2 16.8774 1.64400 7

-7 4 2 21.6673 2.30931 1

-7 4 3 4.57161 1.84296 1

7 -4 3 2.53296 1.47767 7

-7 4 4 3.66440 1.70131 1

7 -4 4 2.79234 1.55711 7

7 -4 5 3.10920 1.63199 7

-8 4 -4 3.43941 1.75488 1

8 4 4 4.07791 1.64541 6

8 4 3 4.01446 1.60187 6

-8 4 -3 3.84049 1.90596 1

8 4 2 3.94369 1.58026 6

-8 4 -2 5.24759 1.97713 1

8 -4 -1 2.72288 1.40230 7

8 4 1 1.59461 1.55041 6

-8 4 -1 4.02095 2.08457 1

8 -4 0 6.92139 1.48217 7

8 4 0 5.63196 1.64724 6

-8 4 0 9.33220 2.22024 1

8 -4 1 1.04561 1.45144 7

8 4 -1 1.73607 1.58382 6

-8 4 1 2.71041 2.13023 1

8 -4 2 3.31101 1.45004 7

-8 4 2 8.54456 2.17113 1

-8 4 3 2.28092 1.94342 1

8 -4 3 1.24146 1.51358 7

-8 4 4 5.26836 1.82959 1

8 -4 4 1.44868 1.53759 7

9 -4 -1 5.90176 1.52010 7

9 -4 0 0.74718 1.44516 7

9 -4 1 6.10817 1.56736 7

0 -5 0-0.46493 1.57157 5

0 -5 -1 0.48111 1.55468 5

0 -5 1-0.97178 1.59625 5

0 -5 -2 0.44475 1.55590 5

0 -5 2-0.84442 1.61499 5

0 -5 -3 4.09674 1.61218 5

0 -5 3 1.54045 1.66812 5

1 -5 -3 11.6829 1.81206 5

-1 -5 3 5.08409 1.76454 5

1 -5 -2 2.57285 1.58179 5

-1 -5 2 0.82064 1.62131 5

1 -5 -1 2.97227 1.57911 5

-1 -5 1 2.14644 1.58133 5

1 -5 0 2.27644 1.62053 5

-1 -5 0 0.03239 1.59256 5

-1 -5 -1 1.49054 1.62289 5

1 -5 1 2.56316 1.70143 5

-1 -5 -2-0.28651 1.49869 5

1 -5 2 0.09826 1.63520 5

-1 -5 -3 5.75781 1.65878 5

1 -5 3 6.20753 1.71189 5

1 -5 4 0.88962 1.72346 5

-2 5 -3 0.67738 2.00688 1

2 -5 -2 25.5575 1.98811 5

-2 -5 2 24.1447 2.08476 5

-2 5 -2 25.4498 2.56820 1

2 -5 -1 34.1632 2.15138 5

-2 -5 1 31.4479 2.11991 5

-2 5 -1 23.4692 2.57463 1

-2 5 0 4.46837 2.14724 1

2 -5 0 3.59979 1.65420 5

-2 -5 0 3.48713 1.64602 5

2 -5 1 35.3984 2.22538 5

-2 -5 -1 32.6249 2.20455 5

-2 5 1 26.2081 2.56904 1

-2 -5 -2 28.8913 2.07898 5

2 -5 2 29.4521 2.22320 5

-2 5 2 27.1982 2.55820 1

-2 5 3 1.88992 1.93451 1

2 -5 3 1.15703 1.60660 5

3 -5 -1 1.76979 1.54531 5

-3 -5 1 2.24740 1.58949 5

-3 5 -1 4.28234 2.12393 1

3 -5 0 11.4574 1.75878 5

-3 -5 0 10.6661 1.70216 5

-3 5 0 11.1724 2.22198 1

-3 5 1 1.76357 2.10388 1

3 -5 1 1.81971 1.57034 5

-3 -5 -1 3.36550 1.60055 5

3 -5 2 8.17522 1.75090 5

-3 -5 -2 9.54988 1.75413 5

4 -5 0 1.06393 1.49399 5

0 0 0 0.00 0.00 0

;

_shelx_hkl_checksum 21209
